# Efficient Low-Scaling Calculation of THC-SOS-LR-CC2 and THC-SOS-ADC(2) Excitation Energies Through Density-Based Integral-Direct Tensor Hypercontraction

**DOI:** 10.1021/acs.jctc.5c00230

**Published:** 2025-05-12

**Authors:** Filippo Sacchetta, Felix H. Bangerter, Henryk Laqua, Christian Ochsenfeld

**Affiliations:** † Chair of Theoretical Chemistry, Department of Chemistry, 9183 University of Munich (LMU), D-81377 Munich, Germany; ‡ Max Planck Institute for Solid State Research, D-70569 Stuttgart, Germany

## Abstract

In recent years, rapid improvements in computer hardware, as well as theoretical and algorithmic advances have enabled the calculation of ever larger systems in computational chemistry. In this avenue, we present efficient implementations of the scaled opposite-spin (SOS) second-order approximate coupled cluster (CC2) method and the closely related second-order algebraic diagrammatic construction (ADC(2)) method. Our implementations applies the least-squares tensor hypercontraction (THC) approximation, for which a new density-based integral-direct reformulation of the grid-projection of the electron integral tensor is presented. Together with screening based on local Cholesky orbitals stemming from the decomposition of the one-particle densities (CDD) in the Laplace integration and optimized block-sparse linear algebra, effectively $O\left(N^{2}\right)$ scaling variants of linear-response (LR) SOS-CC2 and SOS-ADC(2) are obtained. The derived CDD-THC-SOS-LR-CC2/ADC(2) methods are shown to be capable of targeting excitation energies for systems with up to ∼1000 atoms and ∼10,000 basis functions on a single compute node.

## Graphical abstract

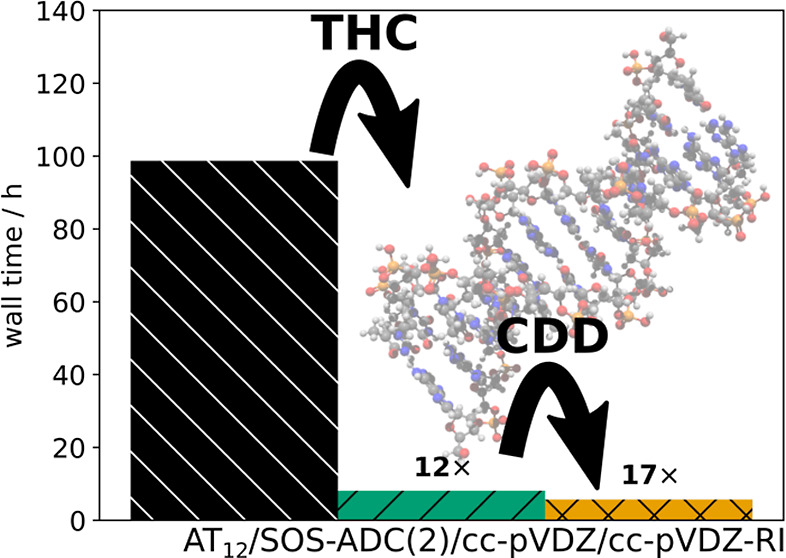

## 1 Introduction

An accurate description of electronic excited states of chemical systems is crucial for the useful interplay of theory and experiment,^1^ where spectroscopy measures transitions between different quantum states, covering electronic, vibrational, or rotational excitations. Among the spectroscopic methods probing transitions between the ground and electronically excited states, UV–vis spectroscopy, which involves the absorption and emission of photons in the ultraviolet (UV) and visible (vis) regions, and X-ray absorption spectroscopy (XAS), which involves the excitation of core electrons, are two of the most widely used methods.^2–4^ Thus, in order to relate the experimentally observed absorption/emission bands to electronic transitions, accurate methods to compute vertical excitation energies are required.

While exact excitation energies can—in the limit of the Born–Oppenheimer approximation and a finite basis set—be obtained through the full configuration interaction (FCI) method,^5,6^ its exponentially scaling cost renders it inapplicable for all but the smallest systems. Therefore, considerable effort has been put into the formulation of approximate methods, also in combination with reduced scaling techniques to reach ever larger system sizes, preferably without sacrificing accuracy. Here, considerable theoretical as well as algorithmic advances have been made in the past decades.^6–8^ The former include reformulations of many of the commonly encountered methods in quantum chemistry under the paradigm of response theory, i.e., the quasidegenerate second-order perturbation corrected configuration interaction singles (CIS­(D_∞_)),^9^ time-dependent density functional theory (TDDFT),^10–12^ the family of complete active space self-consistent field (CASSCF)^13–15^ methods, algebraic diagrammatic construction (ADC)^7,16–19^ methods, as well as linear-response coupled cluster (LR-CC)^20–24^ theory. Here also approximate CC models were introduced, such as the commonly used approximate CC singles and doubles (CC2).^25^ Without further approximations, CC2 and the closely related second-order ADC (ADC(2)) method exhibit quintic scaling, which necessitates additional algorithmic improvements in order for the methods to be applicable to larger systems. While conceptually simple, the scaled opposite-spin (SOS) approximation by Jung et al. achieves a significant reduction of the scaling prefactor by complete neglect of the expensive same-spin terms, while at the same time largely retaining the accuracy.^26–30^ Due to the required transformation of the electron repulsion integral (ERI) tensor into the molecular orbital (MO) basis, the scaling remains as $O\left(N^{5}\right)$ . However, a reduction of the scaling exponent can be achieved by using a factorized form of the ERI tensor, such as the resolution-of-the-identity (RI)^29,31–34^ approximation or the Cholesky decomposition^35,36^ in conjunction with the Laplace transformation^29,37,38^ to obtain a separable form of the orbital energy denominator. Recently, the groups of Ochsenfeld and Dreuw^39,40^ introduced atomic orbital (AO) based formulations of SOS-CC2 and SOS-ADC(2), which achieve subquadratic to linear scaling through a combination of the RI approximation with an attenuated Coulomb metric (ω-RI)^41^ and Cholesky decomposed densities (CDD).^41–46^ To achieve even further reduction of both the memory requirements and the number of required floating point operations (FLOP), tensor hypercontraction (THC) by Martínez and co-workers^47–50^ can be applied, which entirely circumvents the necessity to store and contract third- (or higher-) order tensors. THC variants of CC methods have previously been reported for CC2,^51^ CCSD,^52–54^ and CCSD­(T)^55^ ground state energies as well as for excitation energies based on equation-of-motion (EOM) CC2.^56^

In this work, we propose a density-matrix-based and integral-direct approach for obtaining the THC-factorized ERI tensor, which adds only a small overhead when applied to an electron correlation method. In this regard, SOS-LR-CC2 and SOS-ADC(2) are ideal methods for the application of THC, since they (1) require the AO-ERI tensor to be repeatedly transformed into different MO subspaces, which can efficiently be done by matrix–matrix multiplications, and (2) only include opposite-spin (OS) contributions, for which THC allows to reformulate the expressions to only use matrix linear algebra without the occurrence of any tensors higher than second order. By combining THC with local Cholesky pseudo-MOs from the CDD approach in the Laplace integration and block-sparse linear algebra, effectively $O\left(N^{2}\right)$ scaling formulations of SOS-LR-CC2 and SOS-ADC(2) are obtained. The proposed excited state methods are benchmarked using a set of medium- to large-size systems to assess the scaling of the error with respect to the system size. The accuracy of the ground state energies is analyzed by considering an additional set previously used by DiStasio et al.^57^ for relative energies. Finally, the efficiency of the proposed methods is demonstrated for nucleic acid double helices up to ∼1000 atoms and ∼10,000 basis functions, which reveals overall $O\left(N^{2}\right)$ scaling.

## 2 Theory

### 2.1 Notation

Throughout this work, we employ the following notation:

- μ, ν, λ, σ: atomic orbital indices belonging to the AO basis {χ_μ_} of size *N*_bf_.
- α, β, γ, δ: auxiliary function basis indices belonging to the density fitting basis {χ_α_} of size *N*_aux_ (usually *N*_aux_ ≈ 3·*N*_bf_).
- *P*, *Q*, *R*, *S*: auxiliary function basis indices belonging to the THC basis; in LS-THC these are equivalent to grid points belonging to the LS-THC grid of size *N*_grid_ (usually *N*_grid_ ≈ 3·*N*_aux_).
- *i*, *j*, *k*: occupied molecular orbital indices belonging to the MO basis {ϕ_*i*_} of size *N*_occ_.
- *a*, *b*, *c*: virtual molecular orbital indices belonging to the MO basis {ϕ_*a*_} of size *N*_virt_ (*N*_virt_ ≫ *N*_occ_).
-
i̲,j̲,k̲ : occupied local Cholesky orbitals basis $\left\{\phi_{\underline{\underset{\text{\_}}{i}}}\right\}$ of size *N*_occ_; obtained via pivoted Cholesky decomposition of the occupied one-electron density.
- *p*, *q*, *r*, *s*: general orbital indices.
- τ: root of the Laplace quadrature with *N*_τ_ integration points (usually 5 ≤ *N*_τ_ ≤ 10 is sufficiently accurate).

### 2.2 Integral-Direct Tensor Hypercontraction

#### 2.2.1 Basics of Tensor Hypercontraction

In its most general form, tensor hypercontraction (THC) is a low-dimensional representation of a multidimensional tensor, which—in the context of quantum mechanics—represents the interactions between particles in a system. For a two-body potential $\mathrm{V̂}=1/r_{12}$ , said representation of the electron–electron interactions in a real one-particle basis is the integral tensor, the elements of which are given by

1 $$\left(pq|rs\right)=\iint dr_{1}dr_{2}\varphi_{p}\left(r_{1}\right)\varphi_{q}\left(r_{1}\right)\frac{1}{r_{12}}\varphi_{r}\left(r_{2}\right)\varphi_{s}\left(r_{2}\right)$$

However, in practical quantum chemistry calculations, the manipulation and storage of such a high-dimensional tensor quickly becomes computationally intractable as the system or the basis set size grows. To alleviate this issue, THC provides the means to formally compress the fourth-order integral tensor into 5 second-order tensors as

2 $$\left(pq|rs\right)\approx \sum_{PQ}X_{p}^{P}X_{q}^{P}Z^{PQ}X_{r}^{Q}X_{s}^{Q}$$

The factorization is exact if the number of THC auxiliary functions is at least *N*_bf_(*N*_bf_ + 1)/2,^58^ but only reduces computational demands and storage requirements if it is significantly smaller. If sufficient accuracy is reached with significantly less than *N*_bf_^2^ THC auxiliary functions, the THC factorization enables efficient storage and manipulation of the ERI tensor, making complex calculations feasible for larger systems.

In the least-squares variant of THC (LS-THC),^48^ the THC auxiliary indices are taken to be grid points of a molecular grid similar to the ones commonly used in density function theory. For LS-THC the time-determining step of factorizing the ERI tensor into the THC format is the quintic scaling grid-projection of the ERI tensor, which in the AO basis is given by

3 $$E^{PQ}=\sum_{\mu \nu \lambda \sigma }X_{\mu }^{P}X_{\nu }^{P}\left(\mu \nu |\lambda \sigma \right)X_{\lambda }^{Q}X_{\sigma }^{Q}$$

where the so-called collocation matrices **X** are simply the AO basis functions χ_μ_ evaluated at the THC grid nodes **r**_*P*_ scaled by the node’s weight *w*_*P*_, given as

4 $$X_{\mu }^{P}=\sqrt[{4}]{w_{P}}\chi_{\mu }\left(r_{P}\right)$$

In the general MO formulation, the above equation becomes

5 $$E^{PQ}=\sum_{pqrs}X_{p}^{P}X_{q}^{P}\left(pq|rs\right)X_{r}^{Q}X_{s}^{Q}$$

which requires a transformation of the AO ERI tensor into the MO basis. From **E**, the final **Z** tensor in eq 2 is obtained as

6 $$Z^{PQ}=\sum_{P^{\prime }Q^{\prime }}{\left[S^{-1}\right]}^{PP^{\prime }}E^{P^{\prime }Q^{\prime }}{\left[S^{-1}\right]}^{QQ^{\prime }}$$

where **S** ^–1^ is the inverse of the THC grid metric, i.e., the inverse of

7 $$S^{PP^{\prime }}=\sum_{pq}X_{p}^{P}X_{p}^{P^{\prime }}X_{q}^{P}X_{q}^{P^{\prime }}$$

Note that eq 6 can be solved either by direct inversion of the grid metric,^48^ which generally requires pseudo-inversion due to its rank-deficiency, or by solving the associated system of linear equations.^59,60^

#### 2.2.2 Density-Based Integral-Direct Tensor Hypercontraction

By undoing the AO-to-MO transformations in eq 5, i.e.,

8 $$E^{PQ}=\sum_{pqrs}\sum_{\mu \nu \lambda \sigma }\sum_{\mu^{\prime }\nu^{\prime }\lambda^{\prime }\sigma^{\prime }}X_{\mu }^{P}C_{\mu p}\cdot X_{\nu }^{P}C_{\nu q}\cdot C_{\mu^{\prime }p}C_{\nu^{\prime }q}\left(\mu^{\prime }\nu^{\prime }|\lambda^{\prime }\sigma^{\prime }\right)C_{\lambda^{\prime }r}C_{\sigma^{\prime }s}\cdot X_{\lambda }^{Q}C_{\lambda r}\cdot X_{\sigma }^{Q}C_{\sigma s}$$

and by summing up the MO indices first, the expression can be reformulated in terms of general one-particle density matrices **D** as

9 $$E^{PQ}=\sum_{\mu \nu \lambda \sigma }\sum_{\mu^{\prime }\nu^{\prime }\lambda^{\prime }\sigma^{\prime }}X_{\mu }^{P}X_{\nu }^{P}D_{\mu \mu^{\prime }}D_{\nu \nu^{\prime }}\left(\mu^{\prime }\nu^{\prime }|\lambda^{\prime }\sigma^{\prime }\right)D_{\lambda \lambda^{\prime }}D_{\sigma \sigma^{\prime }}X_{\lambda }^{Q}X_{\sigma }^{Q}$$

which—when contracting the densities with the THC **X** matrices—becomes

10 $$E^{PQ}=\sum_{\mu^{\prime }\nu^{\prime }\lambda^{\prime }\sigma^{\prime }}{\mathrm{X̃}}_{\mu^{\prime }}^{P}{\mathrm{X̃}}_{\nu^{\prime }}^{P}\left(\mu^{\prime }\nu^{\prime }|\lambda^{\prime }\sigma^{\prime }\right){\mathrm{X̃}}_{\lambda^{\prime }}^{Q}{\mathrm{X̃}}_{\sigma^{\prime }}^{Q}$$

To highlight the universality of this approach, we will use the intermediate ${\mathrm{X̃}}_{\mu^{\prime }}^{P}$ as a proxy for both the collocation matrix in the AO basis and when transformed with a general density matrix, i.e.,

11 $${\mathrm{X̃}}_{\mu^{\prime }}^{P}=\sum_{\mu }X_{\mu }^{P}D_{\mu \mu^{\prime }}$$

Therefore, the expressions for the AO-THC and the MO-THC variant only differ in an additional contraction of the THC **X** matrices with a density matrix. Consequently, the same routines can be used for the construction of intermediate **E** in both the AO and an arbitrary MO basis. The latter is particularly convenient since for many correlation methods different kinds of integrals, e.g., (oo|vo), (vo|vo), and (vv|vo) are required. Furthermore, the above expression permits an integral-direct formulation, as outlined in the following, which avoids the prohibitive storage requirements of the full ERI tensor before the transformation into the grid basis. The key idea is that the contraction of the ket side of the ERI tensor can be viewed as *N*_grid_ Coulomb matrix builds with slices of the joint collocation tensor **R**, defined as *R*_λσ_^*Q*^ = *X*_λ_^*Q*^ *X*_σ_^*Q*^, acting as the density matrix. Intermediate **E** in the AO basis from eq 3 can then exemplarily be formed according to algorithm 1.
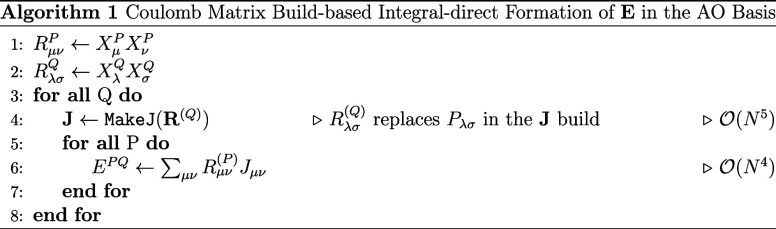


We note here, that for a memory efficient implementation the joint collocation tensor **R** should not be constructed explicitly. Instead, the required tensor slices **R** ^(*Q*)^ can be constructed on-the-fly as a vector outer product of all elements of the **X** tensor belonging to the given grid point *Q*, i.e., $R^{\left(Q\right)}=X_{:}^{Q}⊗{\left(X_{:}^{Q}\right)}^{T}$ .

Together with the idea to reformulate the MO-THC equations in a density-based manner, the Coulomb matrix-based approach permits an efficient and simultaneous formation of all intermediates required for the integrals occurring in CC2/ADC(2) (see Section 2.3.1) according to algorithm 2.
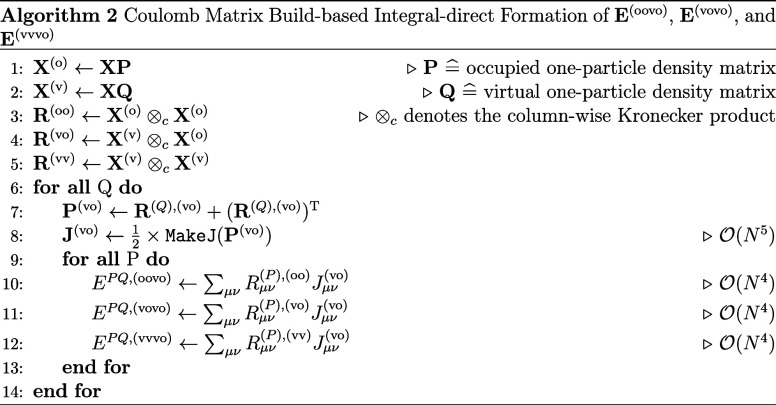


The key ingredient for an efficient implementation is to perform the expensive formation of the **J** intermediate once in the ov space, since all integral types, i.e., (oo|vo), (vo|vo), and (vv|vo), share this as a common ket. We note here, that most routines for the construction of Coulomb-type matrices assume the density to be symmetric. This is not the case for slices of the joint collocation tensor **R** ^(vo)^, which is why the transpose is added in line 7 and the resulting matrix is scaled by a factor of 1/2. Based on the resulting **J** intermediate, the final intermediates **E** for all integral types can be formed simultaneously without further integral evaluations. We also note that the frozen-core approximation can easily be included in this formulation by simply using the frozen-core density matrix for the occupied space.

To lower the formal scaling behavior, the resolution-of-the-identity approximation (RI)^31–34^ can be inserted into eq 10, which allows to perform the grid-projection of the bra and the ket side of the ERI tensor separately at reduced scaling. Inserting the RI approximation into eq 10 leads to

12 EPQ=∑αβγYβP[V−1/2]βα[V−1/2]αγYγQ=∑μ′ν′λ′σ′∑αβγXμ′PXν′P(μ′ν′|β)[V−1/2]βα[V−1/2]αγ(γ|λ′σ′)Xλ′QXσ′Q

where **V** is the two-center RI integral tensor. Intermediate **Y** is given by

13 $$Y_{\beta }^{P}=\sum_{\mu^{\prime }\nu^{\prime }}X_{\mu^{\prime }}^{P}X_{\nu^{\prime }}^{P}\left(\mu^{\prime }\nu^{\prime }|\beta \right)$$

and represents one side of the grid-projected ERI tensor. Like for the **E** intermediate, the formally quartic scaling formation of the **Y** intermediate can be done in an integral-direct fashion. The final factorization then becomes

14 $$\left(pq|rs\right)=\sum_{PQ}\sum_{\alpha }X_{p}^{P}X_{q}^{P}\Gamma_{\alpha }^{P}\Gamma_{\alpha }^{Q}X_{r}^{Q}X_{s}^{Q}$$

with **Γ**, representing one-half of the **Z** tensor, defined as

15 $$\Gamma_{\alpha }^{P}=\sum_{P^{\prime }}\sum_{\beta }{\left[S^{-1}\right]}^{PP^{\prime }}Y_{\beta }^{P^{\prime }}{\left[V^{-1/2}\right]}_{\beta \alpha }$$

Instead of employing routines for the construction of Coulomb matrices, for the **Y** intermediate existing routines for the contraction of density matrices with three-center RI integrals can be used. Among these routines, variants optimized for the contraction of multiple densities, such as the J-engine approach to SOS-RI-MP2 by Maurer et al.^61^ or the RI-J implementation by Kussmann et al.^62^ are employed since *N*_grid_ Coulomb matrix builds need to be performed for each **Y** intermediate. While these algorithms provide performance improvements over a naïve implementation, in which the Coulomb matrix kernel is simply invoked *N*_grid_ times, an optimized integral kernel for this kind of contraction is certainly favorable as outlined below.

#### 2.2.3 Efficient Integral-Direct Algorithm for the **Y** Intermediate

Instead of relying on repetitive J-engine or RI-J based evaluations of the Coulomb potential for many density-like matrices, a more efficient algorithm is proposed inspired by our previous optimal-batching scheme^63^ for evaluating correlation energies on the random phase approximation (RPA) level of theory. In the integral-direct variant shown in algorithm 3, the necessary 3-center-2-electron (3c2e) integrals (μ′ν′|α) and the vector outer products **R** are computed on-the-fly during the formation of the **Y** intermediate removing the unfavorable $O\left(N^{3}\right)$ memory complexity associated with storing the full third-order tensors. The following discussion is exemplariliy carried out for the virtual-occupied subspace but is applicable to all required **Γ** intermediates.
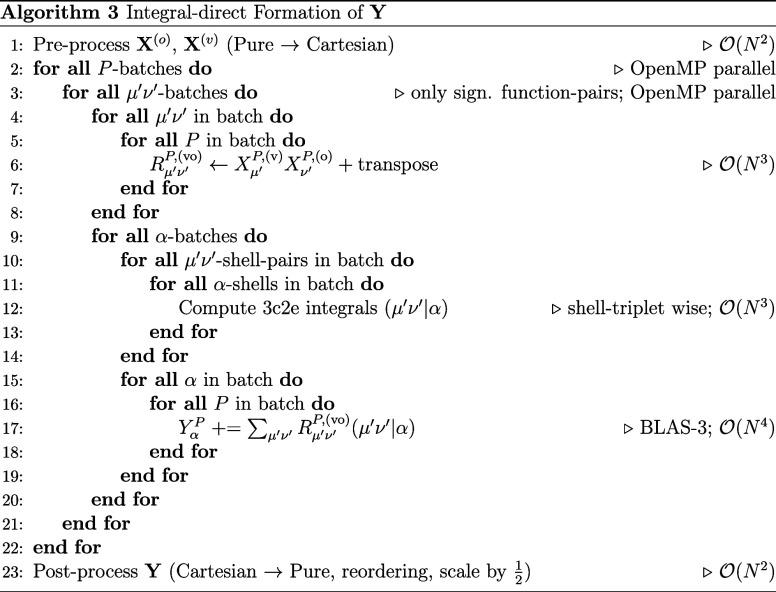


The naïve application of the optimization scheme of ref 63 would predict minimal batch-sizes for the function-pair index μ′ν′ and the auxiliary basis function index α but maximal batch-sizes for the quadrature point index *P*, since the more points *P* are included per batch, the more often any computed given 3c2e integral (μ′ν′|α) can be reused for the computation of **Y**. In practice, however, there are diminishing returns beyond 1000 *P* points per batch, so the batch size is rounded to the nearest power of 2, i.e., 1024 *P* points per batch. The batch sizes for α and μ′ν′ are chosen as 96, which is as small as possible, while still allowing for an efficient execution of the matrix–matrix multiplications within the formation of **Y** in line 17. In practice, the precise batch sizes of each batch slightly deviate from 96 to match a multiple of the number of functions/function-pairs for the respective l-quantum numbers of the shells/shell-pairs within a batch, because the 3c2e integral evaluation is most efficient if operating on full shell-triplets. This approach leads to batches with ∼10,000 elements for (μ′ν′|α) and ∼100,000 elements for $R_{\mu^{\prime }\nu^{\prime }}^{P,\left(ov\right)}$ , making the algorithm cache-friendly, i.e., all necessary quantities can be stored in temporary static random access memory (SRAM) storage (cache) close to the processing units, even for large systems. Moreover, we always aim for multiples of 16 for the innermost loops and order each participating tensor such that the leading index matches the innermost loop. Both design decisions improve the efficiency of memory accesses (cache-lines) and are very favorable for single instruction multiple data (SIMD) vector execution.

In particular, the 3c2e integrals are stored with the auxiliary-shell index as the leading index, so that the 3c2e integral evaluation can make optimal use of SIMD vector routines parallelizing over shell-triplets, i.e., each SIMD-thread handles a separate shell-triplet. For the most efficient use of SIMD vector routines, the auxiliary basis set is considered fully uncontracted, since varying numbers of primitive Gaussian basis functions per shell would otherwise interfere with vectorization, which requires an identical workload for each thread to be efficient. In practice, this is of little concern, since the auxiliary basis sets used for RI-fitting of electron correlation energies (e.g., the Dunning RI basis sets^64–66^ employed in Section 4) are usually completely—or at least mostly—uncontracted already. In addition, the transformation from Cartesian to pure (spherical harmonics) basis functions is not performed at the 3-center integral level, instead the whole **Y**-build is carried out in the Cartesian basis so that only input and output need to be transformed, avoiding any transformations of third-order tensors.

The 3c2e integrals are evaluated using symbolically optimized Obara–Saika^67,68^ recursion relations, similar to the integral kernels used for the 3-center-1-electron (3c1e) integrals within our semi-numerical exchange method (sn-LinK),^69–71^ but adjusted for 2-electron integrals, i.e., by including recurrence relations for both the AO shell-pair and the auxiliary shell. That is, for any given l-quantum number combination the recursion relations are fully expanded symbolically for each final primitive Cartesian integral within the shell-triplet until each integral is solely expressed in terms of primitive Boys integrals. Subsequently the entire set of equations is symbolically optimized by removing redundant subexpressions within the shell-triplet using common-subexpression-elimination (CSE) as provided by the SymPy^72^ package.

Overall, algorithm 3 formally scales as $O\left(N^{4}\right)$ and the matrix–matrix multiplication for the formation of **Y** in line 17 is by far the slowest step. This formal scaling is easily reduced to asymptotically $O\left(N^{3}\right)$ by exploiting the sparsity of the function-pairs μ′ν′, i.e., only function-pairs belonging to significantly overlapping shell-pairs according to the Schwarz integral are considered. In addition, the batch-wise nature of the algorithm is also straightforward to OpenMP parallelize over multiple CPU cores using both the *P*-batch as well as the μ′ν′ batch index for parallelization. While *P*-batch execution is already embarrassingly parallel, parallelization over μ′ν′ requires special treatment of the race-condition associated with accumulation over that index. In practice, the workload is organized such that each thread accumulates as many μ′ν′-batches as possible in a thread-private buffer, so the serial (OpenMP critical section; mutually exclusive between threads) accumulation to the global **Y** needs to be performed as rarely as possible.

### 2.3 THC-CC2 and THC-ADC(2) for Ground and Excited States

#### 2.3.1 Basics of CC2 and ADC(2)

The SOS-CC2 ground state energy in the THC approximation is defined as

16 ESOS‐CC2=⟨HF|Ĥ+cos[Ĥ,T2os]|HF⟩=EHF+cos∑aibjtaitbj[∑β∑RSXaR,(vo)XiR,(vo)ΓβR,(vo)ΓβS,(vo)XbS,(vo)XjS,(vo)]−cos∑aibjt̂aibjos[∑β∑RSXaR,(vo)XiR,(vo)ΓβR,(vo)ΓβS,(vo)XbS,(vo)XjS,(vo)]

where Ĥ is the T1-similarity-transformed Hamiltonian and *T*_2_^os^ is the two-electron excitation operator acting on two electrons with different spins.^27,29^ The cluster amplitudes are determined by solving the CC equations, defined by

17 0=Ωμ1=⟨μ1|Ĥ+cos[Ĥ,T2os]|HF⟩=(ϵa−ϵi)tai+ΩaiG+ΩaiH+ΩaiI+ΩaiJ=(ϵa−ϵi)tai+cos∑cbjt̂cibjos[∑β∑RSXjR,(vo)XbR,(vo)ΓβR,(vo)ΓβS,(vv)X̂aS,(vv)XcS,(vv)]−cos∑kbjt̂akbjos[∑β∑RSXjR,(vo)XbR,(vo)ΓβR,(vo)ΓβS,(oo)XkS,(oo)X̂iS,(oo)]+cos∑bjt̂aibjosF̂jb+F̂ai

18 0=Ωμ2=⟨μ2os|Ĥ+[F,T2os]|HF⟩=ΩaibjE+ΩaibjF=(ϵa−ϵi+ϵb−ϵj)t̂aibjos+[∑α∑PQX̂aP,(vo)X̂iP,(vo)ΓαP,(vo)ΓαQ,(vo)X̂bQ,(vo)X̂jQ,(vo)]

The Fock matrices F̂ are computed using the integral-direct RI-J^73^ and sn-LinK^69–71^ kernels to form the Coulomb and exchange terms with high efficiency. The explicit expressions are provided in the literature.^29,74^ The OS scaling factor is set to *c*_os_ = 1.3, as for SOS-MP2.^27,29^ The solution of eqs 16–18 scales as $O\left(N^{3}\right)$ for the MO-based THC-SOS-CC2 formulation.^56^ However, the MO energies in the denominator of the double amplitudes can be decoupled using Laplace integration

19 $$\frac{1}{\epsilon_{aibj}}=\int_{0}^{\infty }e^{-\epsilon_{aibj}}dt\approx \sum_{\tau }^{n}w_{\tau }e^{-\epsilon_{aibj}t_{\tau }}$$

to rewrite eq 18 as

20 t̂aibjos=−∑τwτ∑α∑PQX̂aP,(vo)X̂iP,(vo)ΓαP,(vo)ΓαQ,(vo)X̂bQ,(vo)X̂jQ,(vo)e−ϵaitτe−ϵbjtτ=−∑τ∑α∑PQX̂a,τP,(vo)X̂i,τP,(vo)ΓαP,(vo)ΓαQ,(vo)X̂b,τQ,(vo)X̂j,τQ,(vo)

The THC X̂ matrices in eqs 16–20 are formed using the transformation matrices

21 $$\Lambda_{\mu a}^{p}=C_{\mu a}-\sum_{i}C_{\mu i}t_{ai},\Lambda_{\mu i}^{h}=C_{\mu i}+\sum_{a}C_{\mu a}t_{ai}$$

according to

22 $${\mathrm{X̂}}_{a,\tau }^{P}=w_{\tau }^{1/4}{\mathrm{X̂}}_{a}^{P}e^{-\epsilon_{a}t_{\tau }},{\mathrm{X̂}}_{a}^{P}=\sum_{\mu }\Lambda_{\mu a}^{p}X_{\mu }^{P}$$

23 $${\mathrm{X̂}}_{i,\tau }^{P}=w_{\tau }^{1/4}{\mathrm{X̂}}_{i}^{P}e^{\epsilon_{i}t_{\tau }},{\mathrm{X̂}}_{i}^{P}=\sum_{\nu }\Lambda_{\nu i}^{h}X_{\nu }^{P}$$

The single amplitudes are obtained by inserting eq 20 into eq 17

24 $$-\left(\epsilon_{a}-\epsilon_{i}\right)t_{ai}=\Omega_{ai}^{G}+\Omega_{ai}^{H}+\Omega_{ai}^{I}+\Omega_{ai}^{J}$$

25 $$\Omega_{ai}^{G}=-c_{os}\sum_{\tau }\sum_{PS}{\mathrm{X̂}}_{i,\tau }^{P,\left(vo\right)}{\mathrm{M̂}}_{\tau }^{PS,\left(ovvv\right)}{\mathrm{X̂}}_{a}^{S,\left(vv\right)}$$

26 $$\Omega_{ai}^{H}=c_{os}\sum_{\tau }\sum_{PS}{\mathrm{X̂}}_{a,\tau }^{P,\left(vo\right)}{\mathrm{N̂}}_{\tau }^{PS,\left(vooo\right)}{\mathrm{X̂}}_{i}^{S,\left(oo\right)}$$

27 $$\Omega_{ai}^{I}=-c_{os}\sum_{\tau }\sum_{P}{\mathrm{X̂}}_{a,\tau }^{P,\left(vo\right)}{\mathrm{X̂}}_{i,\tau }^{P,\left(vo\right)}{\mathrm{n̂}}_{\tau }^{P,\left(vo\right)}$$

28 $$\Omega_{ai}^{J}={\mathrm{F̂}}_{ai}$$

The working equations of the intermediates in eqs 25–27 are provided in the Supporting Information. The evaluation of the MO-THC-SOS-CC2 correction to the ground state energy is then achieved with negligible computational cost as

29 $$E^{SOS‐CC2}=c_{os}\sum_{aibj}\sum_{\beta }\sum_{RS}t_{ai}t_{bj}X_{a}^{R,\left(vo\right)}X_{i}^{R,\left(vo\right)}\Gamma_{\beta }^{R,\left(vo\right)}\Gamma_{\beta }^{S,\left(vo\right)}X_{b}^{S,\left(vo\right)}X_{j}^{S,\left(vo\right)}-c_{os}\sum_{\alpha \beta }{\mathrm{D̂}}_{\tau }^{\alpha \beta }{\mathrm{D̂}}_{\tau }^{\alpha \beta }$$

which can also be used to get the related MO-THC-SOS-MP2 energy correction by simply setting **t**_μ_1__ to zero.

Beyond ground state energies, the SOS-LR-CC2 excitation energies are obtained as eigenvalues of the Jacobian matrix, which is defined as the derivative of the vector functions (eqs 17 and 18) with respect to the cluster amplitudes^25^ and is given by

30 $$A^{SOS‐CC2}=\left(\begin{array}{ll} A_{\mu_{1}\nu_{1}} & A_{\mu_{1}\nu_{2}}\\ A_{\mu_{2}\nu_{1}} & A_{\mu_{2}\nu_{2}} \end{array}\right)=\left(\begin{array}{ll} \left\langle \mu_{1}\left|\left[\left(Ĥ+c_{os}\left[Ĥ,T_{2}^{os}\right]\right),\tau_{\nu_{1}}\right]\right|HF\right\rangle & \left\langle \mu_{1}\left|c_{os}\left[Ĥ,\tau_{\nu_{2}}^{os}\right]\right|HF\right\rangle \\ \left\langle \mu_{2}^{os}\left|\left[Ĥ,\tau_{\nu_{1}}\right]\right|HF\right\rangle & \delta_{\mu_{2}\nu_{2}}\epsilon_{\mu_{2}} \end{array}\right)$$

where the diagonal double–double block (*A*_μ_2_ν_2__) is equal to ϵ_*aibj*_ = ϵ_*a*_ – ϵ_*i*_ + ϵ_*b*_ – ϵ_*j*_. From eq 30, secular matrices of simpler excited states methods are derived. The SOS-CIS­(D_∞_) approach introduced by Head-Gordon et al.^9^ can be derived from SOS-LR-CC2 theory by setting the singles part of the ground state cluster amplitudes **t**_1_ to zero, resulting in a vanishing contribution of the T1-similarity transformed Hamiltonian. The SOS-CIS­(D_∞_) Jacobian matrix is given by

31 $$A^{SOS‐CIS\left(D_{\infty }\right)}=\left(\begin{array}{ll} \left\langle \mu_{1}\left|\left[\left(H+c_{os}\left[H,T_{2}^{os}\right]\right),\tau_{\nu_{1}}\right]\right|HF\right\rangle & \left\langle \mu_{1}\left|c_{os}\left[H,\tau_{\nu_{2}}^{os}\right]\right|HF\right\rangle \\ \left\langle \mu_{2}^{os}\left|\left[H,\tau_{\nu_{1}}\right]\right|HF\right\rangle & \delta_{\mu_{2}\nu_{2}}\epsilon_{\mu_{2}} \end{array}\right)$$

where *T*_2_^os^ denotes the MP2 double amplitudes. In addition, the secular matrix for the SOS-ADC(2) method is related to eq 31 by the symmetrization^29,30,75^

32 $$A^{SOS‐ADC\left(2\right)}=\frac{1}{2}\left[A^{SOS‐CIS\left(D_{\infty }\right)}+{\left(A^{SOS‐CIS\left(D_{\infty }\right)}\right)}^{T}\right]$$

As discussed by Krauter et al.,^30^ it is important to derive the SOS-ADC(2) secular matrix from the SOS-CC2 Jabobian in order to reduce the dimension of the $A_{\mu_{2}\nu_{2}}$ block. In this work, the full CISD­(D_∞_) matrix is not symmetrized but only the $A_{\mu_{1}\nu_{1}}$ block, i.e., the non-Hermiticity of the coupling blocks is retained and only the $A_{\mu_{1}\nu_{2}}$ block is scaled by the factor *c*_os_.^29^ Using the diagonal form of the double–double block, the doubles part of the excitation vector is obtained as

33 $$R_{\mu_{2}}^{m}=-\sum_{\nu_{1}}\frac{A_{\mu_{2}\nu_{1}}R_{\nu_{1}}}{\epsilon_{\gamma_{2}}-{\mathrm{\omega ̅}}^{m}}$$

and the eigenvalue problem of SOS-LR-CC2 and SOS-ADC(2) is solved in the single excitations manifold^29,74^ given by

34 $$\sum_{\nu_{1}}\left[A_{\mu_{1}\nu_{1}}-\sum_{\gamma_{2}}\frac{A_{\mu_{1}\gamma_{2}}A_{\gamma_{2}\nu_{1}}}{\epsilon_{\gamma_{2}}-\mathrm{\omega ̅}}\right]R_{\nu_{1}}=\sum_{\nu_{1}}A_{\mu_{1}\nu_{1}}^{eff}\left(\mathrm{\omega ̅}\right)R_{\nu_{1}}=\sigma_{\mu_{1}}\left(\mathrm{\omega ̅},R_{\nu_{1}}\right)=\mathrm{\omega ̅}R_{\mu_{1}}$$

where the effective **A** matrix is Hermitian for ADC(2) and σ denotes the matrix-vector product.

In order to solve the nonlinear equation part of eq 34, the excitation energies ω̅ and eigenvectors *R*_μ_1__ have to be found iteratively until self-consistency is reached. If the initial guess of the eigenvector and eigenvalue is close enough to the final results, a common choice for the solution of the nonlinear problem is an algorithm based on the direct inversion in the iterative subspace (DIIS) technique,^36,74,76^ which was shown to be stable and rapidly convergent.^29,36^ In addition, it has the advantage of being a single root algorithm, thus allowing to aim for high-lying excited states without converging all lower-lying states. On the contrary, if the initial guess is far from the converged CC2 or ADC(2) result, the eigenvalue and the eigenvector must be pre-optimized using an alternative algorithm. For this purpose, we use a modification of the Davidson algorithm,^74,77^ leveraging the fact that a set of converged eigenvalues and -vectors will fulfill the linear generalized eigenproblem

35 $$\sum_{\mu_{1}\nu_{1}}R_{\mu_{1}}A_{\mu_{1}\nu_{1}}^{eff}\left(\mathrm{\omega ̅}\right)R_{\nu_{1}}=\sum_{\mu_{1}}R_{\mu_{1}}\sigma_{\mu_{1}}\left(\mathrm{\omega ̅},R_{\nu_{1}}\right)=\sum_{\mu_{1}}\mathrm{\omega ̅}R_{\mu_{1}}R_{\mu_{1}}$$

Both the DIIS and the Davidson procedure for CC2 and ADC(2) are described in literature^29,36,74,77^ and will not be discussed here further. The time-determining step for the solution of the eigenvalue problem is the formation of the matrix-vector product

36 $$\sigma_{ai}^{SOS‐CC2}=\sum_{b}{\mathrm{F̂}}_{ab}R_{bi}-\sum_{j}R_{aj}{\mathrm{F̂}}_{ji}+{\mathrm{F̅}}_{ai}-c_{os}\sum_{b}E_{ab}R_{bi}-c_{os}\sum_{j}R_{aj}E_{ji}+\sigma_{ai}^{G,\left(1\right)}\left(R_{\mu_{2}}\right)+\sigma_{ai}^{H,\left(1\right)}\left(R_{\mu_{2}}\right)+\sigma_{ai}^{I,\left(1\right)}\left(R_{\mu_{2}}\right)$$

37 $$\sigma_{ai}^{SOS-ADC\left(2\right)}=\left(\epsilon_{a}-\epsilon_{i}\right)R_{ai}+{\mathrm{F̅}}_{ai}-c_{os}\sum_{b}E_{ab}^{\prime }R_{bi}-c_{os}\sum_{j}R_{aj}E_{ji}^{\prime }+\sigma_{ai}^{G,\left(2\right)}\left(R_{\mu_{2}}\right)+\sigma_{ai}^{H,\left(2\right)}\left(R_{\mu_{2}}\right)+\sigma_{ai}^{I,\left(2\right)}$$

Making use of the Laplace transformation of the energy denominator, the double part of the singlet excitation vector can be rewritten as

38 Rijab(ω̅)=−∑τeω̅tτ∑PQ∑α{[X̅a,τP,(vo)X̂i,τP,(vo)+X̂a,τP,(vo)X̅i,τP,(vo)]ΓαP,(vo)ΓαQ,(vo)X̂b,τQ,(vo)X̂j,τQ,(vo)+[X̅b,τP,(vo)X̂j,τP,(vo)+X̂b,τP,(vo)X̅j,τP,(vo)]ΓαP,(vo)ΓαQ,(vo)X̂a,τQ,(vo)X̂i,τQ,(vo)}

for which the state-specific THC X̅ matrices using the transformation matrices Λ̅

39 $${\mathrm{\Lambda ̅}}_{\mu a}^{p}=\sum_{i}C_{\mu i}R_{ai},{\mathrm{\Lambda ̅}}_{\mu i}^{h}=\sum_{a}C_{\mu a}R_{ai}$$

are given according to

40 $${\mathrm{X̅}}_{a,\tau }^{P}=w_{\tau }^{1/4}{\mathrm{X̅}}_{a}^{P}e^{-\epsilon_{a}t_{\tau }},{\mathrm{X̅}}_{a}^{P}=\sum_{\mu }X_{\mu }^{P}{\mathrm{\Lambda ̅}}_{\mu a}^{p}$$

41 $${\mathrm{X̅}}_{i,\tau }^{P}=w_{\tau }^{1/4}{\mathrm{X̅}}_{i}^{P}e^{\epsilon_{i}t_{\tau }},{\mathrm{X̅}}_{i}^{P}=\sum_{\mu }X_{\mu }^{P}{\mathrm{\Lambda ̅}}_{\mu i}^{h}$$

Inserting eq 38 into eq 36, reduced scaling expressions for the most time-consuming contributions can be formulated as

42 σaiG,(1)=+cos∑jbcRijcb(ω̅)[∑β∑RSXjR,(vo)XbR,(vo)ΓβR,(vo)ΓβS,(vv)X̂aS,(vv)XcS,(vv)]=−cos∑τeω̅tτ∑PS[X̂i,τP,(vo)M̅τPS,(ovvv)+X̅i,τP,(vo)M̂τPS,(ovvv)]X̂aS,(vv)=∑SY̅iS,(vv)X̂aS,(vv)

43 σaiH,(1)=−cos∑jbkRkjab(ω̅)[∑β∑RSXjR,(vo)XbR,(vo)ΓβR,(vo)ΓβS,(oo)XkS,(oo)XiS,(oo)]=+cos∑τeω̅tτ∑PS[X̅a,τP,(vo)N̂τPS,(vooo)+X̂a,τP,(vo)N̅τPS,(vooo)]X̂iS,(oo)=∑SY̅aS,(oo)X̂iS,(oo)

where the contained intermediates are given by

44 M̅τPS,(ovvv)=M̅τ,(1)PS,(ovvv)+M̅τ,(2)PS,(ovvv)=B̂τPS,(ovvv)D̅τPS,(ovvv)+B̅τPS,(ovvv)D̂τPS,(ovvv)

45 N̅τPS,(vooo)=N̅τ,(1)PS,(vooo)+N̅τ,(2)PS,(vooo)=ÂτPS,(vooo)D̅τPS,(vooo)+A̅τPS,(vooo)D̂τPS,(vooo)

46 $${\mathrm{Y̅}}_{i}^{S,\left(vv\right)}=-c_{os}\sum_{\tau }e^{\mathrm{\omega ̅}t_{\tau }}\sum_{P}\left[{\mathrm{X̂}}_{i,\tau }^{P,\left(vo\right)}{\mathrm{M̅}}_{\tau }^{PS,\left(ovvv\right)}+{\mathrm{X̅}}_{i,\tau }^{P,\left(vo\right)}{\mathrm{M̂}}_{\tau }^{PS,\left(ovvv\right)}\right]$$

47 $${\mathrm{Y̅}}_{a}^{S,\left(oo\right)}=+c_{os}\sum_{\tau }e^{\mathrm{\omega ̅}t_{\tau }}\sum_{P}\left[{\mathrm{X̅}}_{a,\tau }^{P,\left(vo\right)}{\mathrm{N̂}}_{\tau }^{PS,\left(vooo\right)}+{\mathrm{X̂}}_{a,\tau }^{P,\left(vo\right)}{\mathrm{N̅}}_{\tau }^{PS,\left(vooo\right)}\right]$$

The contribution σ^I,(1)^ is computed as

48 σaiI,(1)=+cos∑bjtaibjosF̅jb+∑bjcosRijab(ω̅)F̂jb=−cos∑τeω̅tτ∑P[X̅a,τP,(vo)X̂i,τP,(vo)+X̂a,τP,(vo)X̅i,τP,(vo)]ÎτP,(vo)−cos∑τ∑PX̂a,τP,(vo)X̂i,τP,(vo)Îτ,(1)P,(vo)

with

49 $${\mathrm{I̅}}_{\tau ,\left(1\right)}^{P,\left(vo\right)}=\sum_{bj}{\mathrm{X̂}}_{b,\tau }^{P,\left(vo\right)}{\mathrm{X̂}}_{j,\tau }^{P,\left(vo\right)}{\mathrm{F̅}}_{jb}$$

As for the ground state, the Fock-like terms

50 F̅ai=∑ck[2(ai|^kc)−(ac|^ki)]RckF̅ld=∑ck[2(ld|^kc)−(lc|^kd)]Rck

are not reformulated using the THC factorization. Here it is noted that by applying the THC decomposition to eq 37, a similar expression for the SOS-ADC(2) term is obtained. In fact, the contributions **σ** ^G,(2)^ and **σ** ^H,(2)^ are obtained from eqs 42 and 43, respectively, by simply setting **t**_μ_1__ amplitudes to zero. The contribution **σ** ^I,(2)^ is computed according to

51 σaiI,(2)=+cos2∑bjtaibjosF̅jb+cos2∑bj[2(ia|^jb)−(ib|^ja)][∑cktckbjosRck]=−cos2∑τ∑PXa,τP,(vo)Xi,τP,(vo)I̅τ,(1)P,(vo)−cos2∑bj[2(ia|jb)−(ib|ja)]I̅bj

where

52 $${\mathrm{I̅}}_{bj}=\sum_{P}\sum_{\tau }X_{b,\tau }^{P,\left(vo\right)}X_{j,\tau }^{P,\left(vo\right)}{\mathrm{I̅}}_{\tau ,\left(2\right)}^{P,\left(vo\right)}$$

53 $${\mathrm{I̅}}_{\tau ,\left(2\right)}^{P,\left(vo\right)}=\sum_{ck}X_{c,\tau }^{P,\left(vo\right)}X_{k,\tau }^{P,\left(vo\right)}R_{ck}$$

and ${\mathrm{I̅}}_{\tau ,\left(1\right)}^{P,\left(vo\right)}$ is computed as in eq 49 by setting ${\mathrm{X̂}}_{a}^{P}=X_{a}^{P}$ and ${\mathrm{X̂}}_{i}^{P}=X_{i}^{P}$ . The explicit expressions for **E** and $E^{\prime }=\frac{1}{2}\left(E+E^{†}\right)$ in eqs 36 and 37, as well as the working equations for the MO-based matrix-vector product are provided in the Supporting Information.

#### 2.3.2 Reformulation of the Ground State Equations

As demonstrated in our previous work on RI-SOS-CC2,^39^ the equations for the ground state energy can be reformulated in a local basis and hence the intermediates can be expressed in terms of the occupied and virtual one-electron densities

54 $$P_{\mu \nu }=\sum_{i}C_{\mu i}C_{\nu i},Q_{\mu \nu }=\sum_{a}C_{\mu a}C_{\nu a}$$

in order to benefit from the locality of the electronic structure. Despite decreasing the scaling with respect to the system size for the evaluation of most intermediates, the use of the AO basis increases the scaling with respect to the basis set size for a given system. To counteract this, the Cholesky decomposition of the occupied ground state density matrix with complete pivoting^78,79^

55 $$P_{\mu \nu }=\sum_{\underset{\text{\_}}{i}}L_{\mu \underset{\text{\_}}{i}}L_{\nu \mathrm{i̲}}$$

is applied. In addition, the idempotency relation of the occupied and virtual pseudodensity matrices **P** ^τ^ and **Q** ^τ^

56 $$P_{\mu \nu }^{\tau }=w_{\tau }^{1/4}\sum_{i}C_{\mu i}C_{\nu i}e^{\epsilon_{i}t_{\tau }}$$

57 $$Q_{\mu \nu }^{\tau }=w_{\tau }^{1/4}\sum_{a}C_{\mu a}C_{\nu a}e^{-\epsilon_{a}t_{\tau }}$$

58 $$P_{\mu \nu }^{\tau }=\sum_{\sigma \lambda }P_{\mu \sigma }^{\tau }S_{\sigma \lambda }P_{\lambda \nu }=\sum_{\sigma \lambda }\sum_{\mathrm{i̲}}P_{\mu \sigma }^{\tau }S_{\sigma \lambda }L_{\lambda \underset{\text{\_}}{i}}L_{\nu \mathrm{i̲}}$$

59 $$Q_{\mu \nu }^{\tau }=\sum_{\sigma \lambda }Q_{\mu \sigma }^{\tau }S_{\sigma \lambda }Q_{\lambda \nu }$$

is used.

For the solution of the SOS-CC2 equations, another set of asymmetric one-electron density matrices is generated from the T1-transformed coefficients:

60 $${\mathrm{Q̂}}_{\mu \nu }=\sum_{d}C_{\mu d}\Lambda_{\nu d}^{p}=Q_{\mu \nu }-\sum_{\mu^{\prime }\sigma \lambda \nu^{\prime }}Q_{\mu \mu^{\prime }}S_{\mu^{\prime }\sigma }t_{\sigma \lambda }S_{\lambda \nu^{\prime }}P_{\nu^{\prime }\nu }$$

61 $${\mathrm{Q̂}}_{\mu \nu }^{\tau }=\sum_{d}w_{\tau }^{1/4}C_{\mu d}e^{-\epsilon_{d}t_{\tau }}\Lambda_{\nu d}^{p}=Q_{\mu \nu }^{\tau }-\sum_{\mu^{\prime }\sigma \lambda \nu^{\prime }}Q_{\mu \mu^{\prime }}^{\tau }S_{\mu^{\prime }\sigma }t_{\sigma \lambda }S_{\lambda \nu^{\prime }}P_{\nu^{\prime }\nu }$$

62 P̂μν=∑lΛμlhCνl=Pμν+∑μ′σλν′Qμμ′Sμ′σtσλSλν′Pν′ν=∑l_(Lμl̲+∑μ′σλν′Qμμ′Sμ′σtσλSλν′Lν′l̲)Lνl̲=∑l_P̂μl̲Lνl̲

63 P̂μντ=∑iwτ1/4Λμihe(ϵi)tτCνi=∑i_[∑σ′λ′∑j_(Lμj̲+∑ν′σλν″Qμν′Sν′σtσλSλν″Lν″j̲)Lσ′j̲Sσ′λ′Pλ′i̲τ]Lνi̲

To obtain an expression for the single amplitudes in the AO basis, eq 17 in the MO basis is back-transformed to the AO basis according to

64 $$\Omega_{\mu \nu }=\sum_{ai}C_{\mu a}\left(\Omega_{ai}^{G}+\Omega_{ai}^{H}+\Omega_{ai}^{I}+\Omega_{ai}^{J}\right)C_{\nu i}=\Omega_{\mu \nu }^{G}+\Omega_{\mu \nu }^{H}+\Omega_{\mu \nu }^{I}+\Omega_{\mu \nu }^{J}$$

for which the one-electron density matrices in eqs 54–63 permit to rewrite Ωμ__1__ as

65 ΩμνG=∑i_Ωμi̲GLνi̲=∑i_[−cos∑τ∑PS∑μ′X̂i̲,τP,(vo)M̂τPS,(ovvv)Xμ′S,(vv)Q̂μμ′]Lνi̲=∑i_[−cos∑SQ̂μμ′Xμ′S,(vv)Ŷi̲S,(vv)]Lνi̲

66 ΩμνH=∑i_Ωμi̲HLνi̲=∑i_[cos∑τ∑PSX̂μ,τP,(vo)N̂τPS,(vooo)X̂i̲S,(oo)]Lνi̲=∑i_[cos∑SŶμQ,(oo)X̂i̲S,(oo)]Lνi̲

67 $$\Omega_{\mu \nu }^{I}=\sum_{\underset{\text{\_}}{i}}\Omega_{\mu \mathrm{i̲}}^{I}L_{\nu \mathrm{i̲}}=\sum_{\underset{\text{\_}}{i}}\left[-c_{os}\sum_{\tau }\sum_{P}{\mathrm{X̂}}_{\mu ,\tau }^{P,\left(vo\right)}{\mathrm{X̂}}_{\mathrm{i̲},\tau }^{P,\left(vo\right)}{\mathrm{n̂}}_{\tau }^{P,\left(vo\right)}\right]L_{\nu \mathrm{i̲}}$$

68 $$\Omega_{\mu \nu }^{J}=\sum_{\underset{\text{\_}}{i}}\Omega_{\mu \mathrm{i̲}}^{J}L_{\nu \mathrm{i̲}}=\sum_{\underset{\text{\_}}{i}}\left[{\mathrm{Q̂}}_{\mu \mu^{\prime }}{\mathrm{F̂}}_{\mu^{\prime }\nu^{\prime }}{\mathrm{P̂}}_{\nu^{\prime }\mathrm{i̲}}\right]L_{\nu \mathrm{i̲}}$$

Since the **X** matrices are sparse due to the locality of the basis functions, the contraction with the T1-transformed one-electron densities can be performed in linear time as^39,60,70^

69 $${\mathrm{X̂}}_{\mathrm{i̲}}^{P}=\sum_{\mu^{\prime }}X_{\mu^{\prime }}^{P}{\mathrm{P̂}}_{\mu^{\prime }\mathrm{i̲}}$$

70 $${\mathrm{X̂}}_{\mu ,\tau }^{P}=\sum_{\mu^{\prime }}{\mathrm{Q̂}}_{\mu \mu^{\prime }}^{\tau }X_{\mu^{\prime }}^{P}$$

71 $${\mathrm{X̂}}_{\mathrm{i̲},\tau }^{P}=\sum_{\mu^{\prime }}X_{\mu^{\prime }}^{P}{\mathrm{P̂}}_{\mu^{\prime }\mathrm{i̲}}^{\tau }$$

The working equations in the Cholesky basis are provided in Table 1 together with the asymptotic computational scaling of each step.

**Table 1:** Working Equations of the Intermediates for the Solution of the CDD-THC-SOS-CC2/MP2 Equations^a^

	Intermediates	Asymptotic Scaling
(a)	$${Â}_{\tau }^{QR,\left(vovo\right)}={\mathrm{X̂}}_{\mathrm{i̲},\tau }^{Q,\left(vo\right)}X_{\mathrm{i̲}}^{R,\left(vo\right)}$$	O(N)
(b)	$${\mathrm{B̂}}_{\tau }^{QR,\left(vovo\right)}={\mathrm{X̂}}_{\mu ,\tau }^{Q,\left(vo\right)}X_{\mu }^{R,\left(vo\right)}$$	O(N)
(c)	$${Ĉ}_{\tau }^{QR,\left(vovo\right)}={Â}_{\tau }^{QR,\left(vovo\right)}{\mathrm{B̂}}_{\tau }^{QR,\left(vovo\right)}$$	O(N)
(d)	${\mathrm{D̂}}_{\tau }^{\alpha \beta }=\Gamma_{\alpha }^{Q,\left(vo\right)}{Ĉ}_{\tau }^{QR,\left(vovo\right)}\Gamma_{\beta }^{R,\left(vo\right)}$	$O\left(N^{3}\right)$
(e)	${\mathrm{D̂}}_{\tau }^{PS,\left(ovvv\right)}=\Gamma_{\alpha }^{P,\left(vo\right)}{\mathrm{D̂}}_{\tau }^{\alpha \beta }\Gamma_{\beta }^{S,\left(vv\right)}$	$O\left(N^{3}\right)$
(f)	${\mathrm{D̂}}_{\tau }^{PS,\left(vooo\right)}=\Gamma_{\alpha }^{P,\left(vo\right)}{\mathrm{D̂}}_{\tau }^{\alpha \beta }\Gamma_{\beta }^{S,\left(oo\right)}$	$O\left(N^{3}\right)$
(e′)	${\mathrm{D̂}}_{\tau }^{PS,\left(ovvv\right)}=Z^{PQ,\left(vovo\right)}{Ĉ}_{\tau }^{QR,\left(vovo\right)}Z^{RS,\left(ovvv\right)}$	$O\left(N^{2}\right)$
(f′)	${\mathrm{D̂}}_{\tau }^{PS,\left(vooo\right)}=Z^{PQ,\left(vovo\right)}{Ĉ}_{\tau }^{QR,\left(vovo\right)}Z^{RS,\left(vooo\right)}$	$O\left(N^{2}\right)$
(g)	$${Â}_{\tau }^{PS,\left(vooo\right)}={\mathrm{X̂}}_{\mathrm{i̲},\tau }^{P,\left(vo\right)}X_{\mathrm{i̲}}^{S,\left(oo\right)}$$	O(N)
(h)	$${\mathrm{B̂}}_{\tau }^{PS,\left(ovvv\right)}={\mathrm{X̂}}_{\mu ,\tau }^{P,\left(vo\right)}X_{\mu }^{S,\left(vv\right)}$$	O(N)
(i)	$${\mathrm{M̂}}_{\tau }^{PS,\left(ovvv\right)}={\mathrm{D̂}}_{\tau }^{PS,\left(ovvv\right)}{\mathrm{B̂}}_{\tau }^{PS,\left(ovvv\right)}$$	O(N)
(j)	$${\mathrm{N̂}}_{\tau }^{PS,\left(vooo\right)}={\mathrm{D̂}}_{\tau }^{PS,\left(vooo\right)}{Â}_{\tau }^{PS,\left(vooo\right)}$$	O(N)
(k)	$${Ŷ}_{\mu }^{S,\left(oo\right)}={\mathrm{X̂}}_{\mu ,\tau }^{P,\left(vo\right)}{\mathrm{N̂}}_{\tau }^{PS,\left(vooo\right)}$$	O(N)
(l)	$${Ŷ}_{\mathrm{i̲}}^{S,\left(vv\right)}={\mathrm{X̂}}_{\mathrm{i̲},\tau }^{P,\left(vo\right)}{\mathrm{M̂}}_{\tau }^{PS,\left(ovvv\right)}$$	O(N)
(m)	${Î}_{\mathrm{j̲},\tau }^{P,\left(ov\right)}={\mathrm{X̂}}_{\mu ,\tau }^{P,\left(vo\right)}{\mathrm{F̂}}_{\mathrm{j̲}\mu }$	$O\left(N^{2}\right)$
(n)	${\mathrm{n̂}}_{\tau }^{P,\left(ov\right)}={\mathrm{X̂}}_{\mathrm{j̲},\tau }^{P,\left(vo\right)}{Î}_{\mathrm{j̲},\tau }^{P,\left(ov\right)}$	$O\left(N^{2}\right)$

a Throughout this table Einstein’s summation convention is used.

The order of contractions follows the general ideas presented in ref 80. Naturally, to minimize the number of FLOP, common intermediates, such as Â , B̂ , and D̂ should be reused as much as possible. For this, first all possible **X** tensors are contracted over the orbital index. For the subsequent formation of the D̂ intermediates two possible routes have to be considered. In order to minimize the number of FLOP, the **Γ**-factorized form of the THC **Z** tensor should be used and contracted in a total of four matrix–matrix multiplications in steps (d) and (e). However, the fact that the one-electron density becomes sparse for sufficiently large systems with a significant HOMO–LUMO gap can be used here, to perform the step in $O\left(N^{2}\right)$ operations. The first contraction of **Z** with intermediate Ĉ in steps (d′) and (f′) scales quadratically, since Ĉ is sparse, whereas **Z** is not. For the multiplication with the second **Z** matrix the fact that the resulting D̂ matrix is Schur multiplied with either the Â or the B̂ matrix in steps (i) and (j) can be leveraged. Since intermediate Â is sparse, the Schur product with intermediate D̂ will only contain elements which are significant in Â . Therefore, only the elements in D̂ , which are significant in Â need to be computed for step (j) and likewise for step (i) with intermediate B̂ . In total, this enables the formation of the expensive D̂ intermediates in $O\left(N^{2}\right)$ time. All final contractions in steps (k)–(n) are at most quadratically scaling, which results in overall quadratic scaling for the entire CDD-THC-SOS-CC2 method if **Z** is used. Notice that the same strategy can be leveraged when using **Γ** resulting in a further reduction of the computational effort. However, the formal computational complexity is not decreased because only the final matrix–matrix multiplication of steps (e) and (f) would scale quadratically. Therefore, the overall scaling behavior for the entire CDD-THC-SOS-CC2 method is cubic if **Γ** is used.

#### 2.3.3 Reformulation of the Excited State Equations

The strategy outlined in the previous section can likewise be applied to the matrix-vector products of SOS-LR-CC2 and SOS-ADC(2). For simplicity, we will only discuss the reformulation of eq 36 according to

72 $$\sigma_{\mu \nu }^{SOS-CC2,\left(1\right)}=\sum_{ai}C_{\mu a}C_{\nu i}\left(\sum_{b}{\mathrm{F̂}}_{ab}R_{bi}\right)-\sum_{ai}C_{\mu a}C_{\nu i}\left(\sum_{j}R_{aj}{\mathrm{F̂}}_{ji}\right)+\sum_{ai}C_{\mu a}C_{\nu i}{\mathrm{F̅}}_{ai}-c_{os}\sum_{ai}C_{\mu a}C_{\nu i}\left(\sum_{b}E_{ab}R_{bi}\right)-c_{os}\sum_{ai}C_{\mu a}C_{\nu i}\left(\sum_{j}R_{aj}E_{ji}\right)+\sum_{ai}C_{\mu a}C_{\nu i}\left(\sigma_{ai}^{G,\left(1\right)}+\sigma_{ai}^{H,\left(1\right)}+\sigma_{ai}^{I,\left(1\right)}\right)$$

The fourth and fifth term on the right-hand side are rewritten as

73 $$-c_{os}\sum_{ai}C_{\mu a}C_{\nu i}\left(\sum_{b}E_{ab}R_{bi}\right)=-c_{os}\sum_{\mu^{\prime }\sigma \sigma^{\prime }\nu^{\prime }}{Ê}_{\mu^{\prime }\nu^{\prime }}^{\prime }Q_{\nu^{\prime }\sigma^{\prime }}\left[\sum_{\lambda^{\prime }\lambda }S_{\sigma^{\prime }\lambda^{\prime }}R_{\lambda^{\prime }\lambda }S_{\lambda \sigma }\right]P_{\sigma \nu }$$

74 $$-c_{os}\sum_{ai}C_{\mu a}C_{\nu i}\left(\sum_{j}R_{aj}E_{ji}\right)=-c_{os}\sum_{\mu^{\prime }\sigma \sigma^{\prime }\nu^{\prime }}Q_{\mu \mu^{\prime }}\left[\sum_{\lambda \lambda^{\prime }}S_{\mu^{\prime }\lambda^{\prime }}R_{\lambda \lambda^{\prime }}S_{\lambda \sigma }\right]P_{\sigma \sigma^{\prime }}{Ê}_{\sigma^{\prime }\nu^{\prime }}^{\prime \prime }$$

where the intermediates **E**, given by

75 $$E_{\mu \nu }^{\prime }=\sum_{S}\left(\sum_{\tau }\sum_{P}{\mathrm{X̂}}_{\mu ,\tau }^{P,\left(vo\right)}{\mathrm{N̂}}_{\tau }^{PS,\left(vovo\right)}\right)X_{\nu }^{S,\left(vo\right)}$$

76 $$E_{\mu \nu }^{\prime \prime }=\sum_{\mathrm{j̲}\mathrm{i̲}}L_{\mu \mathrm{j̲}}L_{\nu \mathrm{i̲}}\left[\sum_{S}\left(\sum_{\tau }\sum_{P}{\mathrm{X̂}}_{\mathrm{i̲},\tau }^{P,\left(vo\right)}{\mathrm{M̂}}_{\tau }^{PS,\left(vovo\right)}\right)X_{\mathrm{j̲}}^{S,\left(vo\right)}\right]$$

depend only on ground state quantities and hence are computed once and stored on disk. The terms **σ** ^G,(1)^, **σ** ^H,(1)^ and **σ** ^I,(1)^ are given as

77 σμνG,(1)=−cos∑μ′i̲Lνi̲{∑τeω̅tτ∑PS[X̂i̲,τP,(vo)M̅τPS,(ovvv)+X̅i̲,τP,(vo)M̂τPS,(ovvv)]Xμ′S,(vv)}Q̂μμ′=−cos∑S∑μ′i̲Lνi̲Y̅i̲S,(vv)Xμ′S,(vv)Q̂μμ′

78 σμνH,(1)=+cos∑i_Lνi̲{∑τeω̅tτ∑PS[X̅μ,τP,(vo)N̂τPS,(vooo)+X̂μ,τP,(vo)N̅τPS,(vooo)]X̂i̲S,(oo)}=+cos∑S∑i_Y̅μS,(oo)X̂i̲S,(oo)Lνi̲

79 σμνI,(1)=−cos∑i_Lνi̲{∑τeω̅tτ∑P[X̅μ,τP,(vo)X̂i̲,τP,(vo)+X̂μ,τP,(vo)X̅i̲,τP,(vo)]ÎτP,(vo)}−cos∑i_Lνi̲{∑τ∑PX̂μ,τP,(vo)X̂i̲,τP,(vo)I̅τ,(1)P,(vo)}

with

80 $${\mathrm{I̅}}_{\tau ,\left(1\right)}^{P,\left(vo\right)}=\sum_{\mu \nu \mathrm{i̲}}{\mathrm{X̂}}_{\mu ,\tau }^{P,\left(vo\right)}{\mathrm{X̂}}_{\mathrm{i̲},\tau }^{P,\left(vo\right)}{\mathrm{F̅}}_{\mu \nu }L_{\nu \mathrm{i̲}}$$

The matrices M̅ and N̅ , defined in eqs 44 and 45, are computed as shown in Table 2. The general approach for the order of contractions and the reduction of the scaling is similar to the ideas presented for Table 1. The state-specific THC X̅ matrices are given by

81 $${\mathrm{X̅}}_{\mu ,\tau }^{P,\left(vo\right)}=\sum_{\mu^{\prime }}{\mathrm{Q̅}}_{\mu \mu^{\prime }}^{\tau }X_{\mu^{\prime }}^{P,\left(vo\right)},{\mathrm{X̅}}_{\mathrm{i̲},\tau }^{P,\left(vo\right)}=\sum_{\mu }{\mathrm{P̅}}_{\mu \mathrm{i̲}}^{\tau }X_{\mu }^{P,\left(vo\right)}$$

where the densities Q̅ and P̅ are defined as

82 $${\mathrm{Q̅}}_{\mu \nu }^{\tau }=\sum_{a}w_{\tau }^{1/4}C_{\mu a}e^{-\epsilon_{a}t_{\tau }}{\mathrm{\Lambda ̅}}_{\nu a}^{p}=-\sum_{\mu^{\prime }\nu^{\prime }\mathrm{k̲}}Q_{\mu \mu^{\prime }}^{\tau }\left[\sum_{\sigma \lambda }S_{\mu^{\prime }\sigma }R_{\sigma \lambda }S_{\lambda \nu^{\prime }}\right]L_{\nu^{\prime }\mathrm{k̲}}L_{\mathrm{k̲}\nu }$$

83 $${\mathrm{P̅}}_{\mu \nu }^{\tau }=\sum_{i}w_{\tau }^{1/4}{\mathrm{\Lambda ̅}}_{\mu i}^{h}e^{\epsilon_{i}t_{\tau }}C_{\nu i}=\left\{\sum_{\mu^{\prime }\nu^{\prime }}\;\sum_{\mathrm{j̲}\sigma^{\prime }\lambda^{\prime }}Q_{\mu \mu^{\prime }}\left[S_{\mu^{\prime }\lambda }R_{\lambda \sigma }S_{\sigma \nu^{\prime }}\right]L_{\nu^{\prime }\mathrm{j̲}}L_{\sigma^{\prime }\mathrm{j̲}}S_{\sigma^{\prime }\lambda^{\prime }}P_{\lambda^{\prime }\mathrm{i̲}}^{\tau }\right\}L_{\nu \mathrm{i̲}}$$

contain the information about the electronic transition. As discussed in Section 2.3.1, the SOS-ADC(2) matrix-vector product is easily obtained by setting the single amplitudes to zero and symmetrizing the intermediates in eqs 75 and 76. Notice that, eq 51 can be reformulated as

84 σμνI,(2)=−cos∑i_Lνi̲{∑τ∑PXμ,τP,(vo)Xi̲,τP,(vo)I̅τ,(1)P,(vo)}−cos∑μ′ν′∑i_Qμν′{∑σλ[2(μ′ν′|σλ)−(μ′λ|σν′)]Lσk̲I̅λk̲}Lμ′i̲Lνi̲

85 $${\mathrm{I̅}}_{\lambda \mathrm{i̲}}=\sum_{P}\sum_{\tau }X_{\lambda ,\tau }^{P,\left(vo\right)}X_{\mathrm{i̲},\tau }^{P,\left(vo\right)}{\mathrm{I̅}}_{\tau ,\left(2\right)}^{P,\left(vo\right)}$$

86 $${\mathrm{I̅}}_{\tau ,\left(2\right)}^{P,\left(vo\right)}=\sum_{\mu \mathrm{i̲}}X_{\mu ,\tau }^{P,\left(vo\right)}X_{\mathrm{i̲},\tau }^{P,\left(vo\right)}\left[\sum_{\nu \lambda \sigma \nu^{\prime }}Q_{\mu \nu }S_{\nu \lambda }R_{\lambda \sigma }S_{\sigma \nu^{\prime }}L_{\nu^{\prime }\mathrm{i̲}}\right]$$

**Table 2:** Working Equations for the Evaluation of the σ^G^ and σ^H^ Contributions to the CDD-THC-SOS-ADC(2)/LR-CC2 Matrix-Vector Product^a^

	Intermediates	Asymptotic Scaling
(a)	$${\mathrm{A̅}}_{\tau }^{QR,\left(vovo\right)}={\mathrm{X̅}}_{\mathrm{i̲},\tau }^{Q,\left(vo\right)}X_{\mathrm{i̲}}^{R,\left(vo\right)}$$	O(N)
(b)	$${\mathrm{B̂}}_{\tau }^{QR,\left(vovo\right)}={\mathrm{X̂}}_{\mu ,\tau }^{Q,\left(vo\right)}X_{\mu }^{R,\left(vo\right)}$$	O(N)
(c)	$${\mathrm{C̅}}_{\tau }^{QR,\left(vovo\right)}={\mathrm{A̅}}^{QR,\left(vovo\right)}{\mathrm{B̂}}^{QR,\left(vovo\right)}+{Â}^{QR,\left(vovo\right)}{\mathrm{B̅}}^{QR,\left(vovo\right)}$$	O(N)
(d)	${\mathrm{D̅}}_{\tau }^{P\beta ,\left(vo\right)}=\Gamma_{\alpha }^{P,\left(vo\right)}\left(\Gamma_{\alpha }^{Q,\left(vo\right)}{\mathrm{C̅}}_{\tau }^{QR,\left(vovo\right)}\Gamma_{\beta }^{R,\left(vo\right)}\right)=\Gamma_{\alpha }^{P,\left(vo\right)}{\mathrm{D̅}}_{\tau }^{\alpha \beta }$	$O\left(N^{2}\right)$
(e)	${\mathrm{D̅}}_{\tau }^{PS,\left(ovvv\right)}=e^{\mathrm{\omega ̅}t_{\tau }}{\mathrm{D̅}}_{\tau }^{P\beta ,\left(vo\right)}\Gamma_{\beta }^{S,\left(vv\right)}$	$O\left(N^{2}\right)$
(f)	${\mathrm{D̅}}_{\tau }^{PS,\left(vooo\right)}=e^{\mathrm{\omega ̅}t_{\tau }}{\mathrm{D̅}}_{\tau }^{P\beta ,\left(vo\right)}\Gamma_{\beta }^{S,\left(oo\right)}$	$O\left(N^{2}\right)$
(e′)	${\mathrm{D̅}}_{\tau }^{PS,\left(ovvv\right)}=e^{\mathrm{\omega ̅}t_{\tau }}Z^{PQ,\left(vovo\right)}{\mathrm{C̅}}_{\tau }^{QR,\left(vovo\right)}Z^{RS,\left(ovvv\right)}$	$O\left(N^{2}\right)$
(f′)	${\mathrm{D̅}}_{\tau }^{PS,\left(vooo\right)}=e^{\mathrm{\omega ̅}t_{\tau }}Z^{PQ,\left(vovo\right)}{\mathrm{C̅}}_{\tau }^{QR,\left(vovo\right)}Z^{RS,\left(vooo\right)}$	$O\left(N^{2}\right)$
(g)	$${\mathrm{A̅}}_{\tau }^{PS,\left(vooo\right)}={\mathrm{X̅}}_{\mathrm{i̲},\tau }^{P,\left(vo\right)}X_{\mathrm{i̲}}^{S,\left(oo\right)}$$	O(N)
(h)	$${\mathrm{B̅}}_{\tau }^{PS,\left(ovvv\right)}={\mathrm{X̅}}_{\mu ,\tau }^{P,\left(vo\right)}X_{\mu }^{S,\left(vv\right)}$$	O(N)
(i)	$${\mathrm{M̅}}_{\tau }^{PS,\left(ovvv\right)}={\mathrm{M̅}}_{\tau ,\left(1\right)}^{PS,\left(ovvv\right)}+{\mathrm{M̅}}_{\tau ,\left(2\right)}^{PS,\left(ovvv\right)}$$	O(N)
(j)	$${\mathrm{M̅}}_{\tau ,\left(1\right)}^{PS,\left(ovvv\right)}={\mathrm{B̂}}_{\tau }^{PS,\left(ovvv\right)}{\mathrm{D̅}}_{\tau }^{PS,\left(ovvv\right)}$$	O(N)
(k)	$${\mathrm{M̅}}_{\tau ,\left(2\right)}^{PS,\left(ovvv\right)}={\mathrm{B̅}}_{\tau }^{PS,\left(ovvv\right)}{\mathrm{D̂}}_{\tau }^{PS,\left(ovvv\right)}$$	O(N)
(l)	$${\mathrm{N̅}}_{\tau }^{PS,\left(vooo\right)}={\mathrm{N̅}}_{\tau ,\left(1\right)}^{PS,\left(vooo\right)}+{\mathrm{N̅}}_{\tau ,\left(2\right)}^{PS,\left(vooo\right)}$$	O(N)
(m)	$${\mathrm{N̅}}_{\tau ,\left(1\right)}^{PS,\left(vooo\right)}={Â}_{\tau }^{PS,\left(vooo\right)}{\mathrm{D̅}}_{\tau }^{PS,\left(vooo\right)}$$	O(N)
(n)	$${\mathrm{N̅}}_{\tau ,\left(2\right)}^{PS,\left(vooo\right)}={\mathrm{A̅}}_{\tau }^{PS,\left(vooo\right)}{\mathrm{D̂}}_{\tau }^{PS,\left(vooo\right)}$$	O(N)
(o)	$${\mathrm{Y̅}}_{\mu }^{S,\left(oo\right)}={\mathrm{X̅}}_{\mu ,\tau }^{P,\left(vo\right)}{\mathrm{N̂}}_{\tau }^{PS,\left(vooo\right)}+{\mathrm{X̂}}_{\mu ,\tau }^{P,\left(vo\right)}{\mathrm{N̅}}_{\tau }^{PS,\left(vooo\right)}$$	O(N)
(p)	$${\mathrm{Y̅}}_{\mathrm{i̲}}^{S,\left(vv\right)}={\mathrm{X̂}}_{\mathrm{i̲},\tau }^{P,\left(vo\right)}{\mathrm{M̅}}_{\tau }^{PS,\left(ovvv\right)}+{\mathrm{X̅}}_{\mathrm{i̲},\tau }^{P,\left(vo\right)}{\mathrm{M̂}}_{\tau }^{PS,\left(ovvv\right)}$$	O(N)

a Throughout this table Einstein’s summation convention is used. The ADC(2) equations are obtained by setting **t**_μ_1__ = 0.

In order to minimize the number of FLOP, common intermediates, such as A̅ , B̅ , D̅ should be reused as much as possible. For this, first all possible **X** tensors are contracted over the orbital index. The fact that the one-electron density and transition density become sparse can be used here to form the D̅ intermediates with $O\left(N^{2}\right)$ scaling behavior. As discussed in Section 2.3.2, it is possible to carry out the computation of the D̅ intermediates using two possible routes. To obtain the theoretical quadratic scaling behavior in the asymptotic limit, the THC **Z** tensor can be used and contracted in a total of two matrix–matrix multiplications in steps (e′ and f′) of Table 2. The first contraction of **Z** with intermediate C̅ in steps (e′ and f′) scales quadratically, since C̅ is significantly sparse whereas **Z** is not. For the multiplication with the second **Z** matrix the fact that the resulting D̅ matrix is Schur multiplied with either the Â or the B̂ matrix in steps (i and j) can be leveraged. Since intermediate Â is sparse, the Schur product with intermediate D̂ will only contain elements that are significant in Â . Therefore, only the elements in D̅ , which are significant in Â need to be computed for step (j) and likewise for step (i) with intermediate B̂ . In total, this enables the formation of the expensive D̅ intermediates in $O\left(N^{2}\right)$ time. In order to minimize the number of FLOP, the **Γ**-factorized form of the THC **Z** tensor should be used and contracted in a total of four matrix–matrix multiplications in steps (d)–(f) of Table 2. Leveraging the fact that the C̅ , A̅ , and B̅ intermediates are significantly sparse (for large systems and local electronic excitations) steps (d)-(f) in Table 2 effectively scale as $O\left(N^{2}\right)$ in the asymptotic limit.

The ground state intermediates D̂ are recomputed as in Table 1 for each new matrix-vector product and used in steps (k) and (n) of Table 2. By leveraging the sparsity of B̅ in step (k) and A̅ in step (n), it is possible to compute D̂ with quadratic time complexity. All contractions in steps (i)-(p) are at most linear scaling, which results in overall asymptotic quadratic scaling for the entire CDD-THC-SOS-LR-CC2/ADC(2) methods.

## 3 Computational Details

The presented CDD-THC-SOS-LR-CC2 and CDD-THC-SOS-ADC(2) methods as well as the MO-based variants are implemented in the FermiONs++ ^81–83^ program. The program was compiled with the Intel C/C++ Compiler 2022.0.2 and linked against the Intel Math Kernel Library 2022.0.2 for the employed matrix algebra. All developed code was—as far as possible—parallelized with OpenMP.^84^ The underlying Hartree–Fock calculations have been converged to a maximum element of the error matrix in the DIIS procedure below 10^–7^. In the SCF, the Coulomb matrix is evaluated using the RI-J approach by Kussmann et al.^73^ using the cc-pVDZ-JKfit or cc-pVTZ-JKfit^85^ RI-J basis set, respectively. For the exchange matrix, the seminumerical linear scaling exchange method by Laqua et al.^70,71^ is used. For the solution of the least-squares equations in THC the pivoted Cholesky decomposition ansatz by Matthews^59^ is used. The hand-optimized grid for the cc-pVTZ basis set by Kokkila Schumacher et al.^86^ is used as a parent grid together with a pruning threshold of 10^–10^, unless otherwise noted. As demonstrated in the Supporting Information, it is pivotal to reorder the grid points after the pruning, since the pivoting in the Cholesky decomposition significantly reduces the sparsity of intermediates based on the pruned collocation matrices. The computational performance of the CDD-THC-SOS-LR-CC2 and CDD-THC-SOS-ADC(2) methods is improved using sparse linear algebra. The current implementation leverages block-sparse (BS) matrices, which divide the matrices into smaller blocks of maximum size 96 × 96. Further information about the BS matrix implementation is provided in the Supporting Information of ref 39. The thresholds controlling the storage sparsity (ϑ_*a*_) and the matrix–matrix multiply sparsity (ϑ_*m*_) are set to 10^–7^ and 10^–9^, respectively. The threshold controlling the storage sparsity of the transition density matrix is set to 10^–4^. The single cluster amplitudes and ground state energy at the SOS-CC2 level are optimized via the DIIS procedure, which terminates when the L2-norm of the single vector function is lower than 10^–5^ and the variation in the energy is lower than 10^–6^. For excited states calculations, the trial excitation vectors and energies are first optimized at the ADC(1)/CCS level via the Davidson procedure, until the L2-norm of residuals and the variation in the eigenvalues are below 10^–3^. Then, SOS-LR-CC2 or SOS-ADC(2) excitation vectors and energies are optimized using the DIIS algorithm, which terminates when the L2-norm of the residuals are lower than 10^–5^ and the variations in the eigenvalues are lower than 10^–6^. It is important to note that the ADC(1)/CCS trial vectors and eigenvalues can be pre-optimized via the Davidson procedure at the CC2 or ADC(2) level if the DIIS procedure fails to converge to the correct root. Optimized minimax grids with 7 quadrature points for the Laplace expansion are used for both ground and excited states calculations. Moreover, the frozen-core approximation is used in the THC fitting as well as in the ground state and excited states calculations. Notice that calculations are carried out using steps (d)–(f) in Tables 1 and 2. Throughout, the Dunning cc-pVXZ (X ∈ {D,T}) basis sets^64,65^ are used together with their corresponding RI basis sets.^66^ All calculations are performed using a compute node with two AMD EPYC 7452 32-Core 2.35 GHz CPUs (64 cores in total), 1 TB of RAM, and 24 TB of disk space. All runtimes are reported as wall times, not CPU times.

## 4 Results

### 4.1 Accuracy

The present work aims to increase the efficiency of the SOS-ADC(2) and SOS-LR-CC2 methods to extend their applicability to molecules with several hundreds of atoms while retaining accuracy as far as possible. To assess the accuracy of the ground state implementation, the errors produced by the MO-based as well as by the CDD-based THC-SOS-MP2/CC2 methods are investigated with respect to MO-RI-SOS-MP2/CC2. For this purpose, the THC error Δ*E*_THC_ is defined as |*E*_MO‑RI‑SOS‑MP2/CC2_ – *E*_MO‑THC‑SOS‑MP2/CC2_|, i.e., measuring the error introduced by the THC approximation alone, whereas the CDD error Δ*E*_CDD_ is defined as |*E*_MO‑THC‑SOS‑MP2/CC2_ – *E*_CDD‑THC‑SOS‑MP2/CC2_|, i.e., measuring the error introduced by CDD reformulation and the associated use of sparse matrix algebra. For the assessment of the accuracy for excitation energies, the definition of the Δ*E*_THC_ error and the Δ*E*_CDD_ error is analogous.

#### 4.1.1 THC Error

Table 3 summarizes the THC errors in terms of the mean absolute deviations (MAD), the maximum absolute errors (MAX), and the root-mean-square deviations (RMSD) for ground state calculations on three different benchmark sets: (1) the Thiel benchmark set,^87^ (2) the benchmark set used by Hohenstein et al.^56^ in their work on THC-EOM-CC2, and (3) a benchmark set comprised of 27 alanine tetrapeptide conformers.^57^ For these sets of small and medium sized molecules, the CDD errors are negligible, cf. Section S5 of the Supporting Information containing detailed results for the three different benchmark sets.

**Table 3:** Mean Absolute Errors for the Three Different Benchmark Sets in refs 56, 57 and 87 ^a^

		Δ*E*_THC_/eV	
		SOS-MP2	SOS-CC2
Thiel^87^			
[Abs. Energy]			
	MAD	<0.001	0.006
	MAX	0.001	0.021
	RMSD	<0.001	0.008
Hohenstein^56^			
[Abs. Energy]			
	MAD	0.002	0.024
	MAX	0.004	0.055
	RMSD	0.002	0.029
Alanine tetrapeptides^57^			
[Abs. Energy]			
	MAD	<0.001	0.036
	MAX	0.001	0.039
	RMSD	<0.001	0.036
[Rel. Energy]			
	MAD	<0.001	0.004
	MAX	<0.001	0.043
	RMSD	<0.001	0.012

a Errors are reported as mean absolute deviations (MAD), maximum absolute errors (MAX), and root mean square deviations (RMSD) as obtained by the MO-THC-SOS-MP2/CC2 methods relative to the MO-RI-SOS-MP2/CC2 results. All calculations are performed with the cc-pVTZ/cc-pVTZ-RI basis set combination.

Regarding the accuracy of the THC approximation in MP2, MO-THC-SOS-MP2 shows only small deviations of the absolute energies on the order of ∼10^–4^ to 10^–3^ eV compared to the reference method MO-RI-SOS-MP2. For CC2, however, the use of THC in MO-THC-SOS-CC2 produces errors that are roughly 1 order of magnitude larger with a maximum error of 0.055 eV. These errors exhibit only modest error cancellation for relative energies, e.g., the RMSD for alanine tetrapeptides^57^ reduces from 0.036 to 0.012 eV going from absolute to relative energies and the maximum error even slightly increases from 0.039 to 0.043 eV. The larger THC errors in CC2 compared to MP2 are due to the inclusion of (ov|vv)-type integrals in the THC fitting as these are necessary for the iterative solution of the CC2 equations, whereas MP2 only requires (ov|ov)-type integrals. Fitting the much larger (ov|vv) subspace with the THC grids—originally optimized for MP2 ^86^—is substantially more challenging which explains the observed increase in the THC error going from MP2 to CC2. Note that these findings regarding larger THC errors for CC2 compared to MP2 are mostly in line with previous work,^51,52^ albeit somewhat more pronounced due to our more comprehensive collection of benchmark sets and the use of the larger triple-ζ basis sets (cc-pVTZ/cc-pVTZ-RI) in contrast to the double-ζ basis sets used previously. Nevertheless, since the errors are below ∼0.1 eV, they are considered to be acceptable in most applications and are still substantially smaller than the method error of CC2 itself.

Going beyond ground state energies, the applicability of THC-SOS-CC2/ADC(2) for excitation energies is assessed by comparing MO-THC-SOS-LR-CC2/ADC(2) with the RI-based reference implementation. The absolute deviations are summarized in Table 4 for excitations to the three lowest-lying singlet (S) and triplet (T) states of the molecules in the Thiel benchmark set,^87^ as well as in the benchmark set used by Hohenstein et al.^56^ Analogous to the situation for ground state energies, the CDD errors here are also virtually zero, due to the small size of the molecules within the considered benchmark sets. Detailed data including the error for each individual molecule is provided in Section S5 of the Supporting Information.

**Table 4:** Errors for the Excitation Energies to the Three Lowest-Lying Singlet and Triplet Excited States of the Molecules From the Benchmark Sets From Refs 56 and 87 ^a^

		Δ*E*_THC_/eV	
		SOS-ADC(2)	SOS-LR-CC2
Thiel^87^			
[singlet]			
	MAD	0.010	0.019
	MAX	0.050	0.055
	RMSD	0.012	0.023
[triplet]			
	MAD	0.007	0.018
	MAX	0.026	0.052
	RMSD	0.008	0.021
Hohenstein^56^			
[singlet]			
	MAD	0.014	0.017
	MAX	0.041	0.067
	RMSD	0.007	0.023
[triplet]			
	MAD	0.009	0.014
	MAX	0.018	0.035
	RMSD	0.004	0.012

a Errors are reported as mean absolute deviations (MAD), maximum absolute errors (MAX), and root mean square deviations (RMSD) from the reference energies obtained with MO-RI-SOS-LR-CC2/ADC(2). All calculations are performed with the cc-pVTZ/cc-pVTZ-RI basis set combination.

Compared to the results for ground state energies, the application of THC to SOS-ADC(2) and SOS-LR-CC2 produces errors that are larger with mean absolute errors up to ∼0.019 eV and maximum errors up to 0.067 eV. This large maximum error is exceptional, as only the excitation to the THC-SOS-LR-CC2 first singlet excited states of acridine-red from the Hohenstein benchmark set shows a deviation as large as ∼0.067 eV. Leaving this outlier, all deviations are within ∼0.055 eV, which we would consider acceptable for most applications. As expected THC-SOS-ADC­(2) consistently delivers errors smaller than THC-SOS-LR-CC2. Moreover, triplet excitations are described more accurately compared to singlet excitations, with the largest observed deviation being 0.026 eV for THC-SOS-ADC(2) and 0.052 eV for THC-SOS-LR-CC2. Again, these errors are in line with previous work,^56^ although the exceptional cases with large errors were not seen before and are probably a consequence of the more comprehensive data sets and the use of larger triple-ζ basis sets. Unfortunately, improving the accuracy of THC beyond the limitations imposed by the predefined THC grids is not trivial and beyond the scope of this work. Instead, we rather focus on the reduced-scaling local CDD-based reformulation and its advantages compared to the nonlocal MO-based RI reference algorithms, particularly in terms of accessible molecule sizes.

#### 4.1.2 CDD Error

After having assessed the intrinsic errors associated with LS-THC, we now turn our attention toward the errors introduced by the local Cholesky-MOs and the sparse matrix algebra. Since the errors are only significant for larger molecules, the assessment provided in Tables 5 and 6 is carried out for a series of linear carboxylic acids (LCA_*n*_) and adenine-thymine base pair stacks (AT_*n*_).

**Table 5:** Absolute Δ*E*_THC_ and Δ*E*_CDD_ Errors for the Ground State Energies for a Series of LCA_*n*_ and AT_*n*_ Molecules^a^

	SOS-MP2		SOS-CC2	
	Δ*E*_THC_/meV	Δ*E*_CDD_/meV	Δ*E*_THC_/meV	Δ*E*_CDD_/meV
LCA_40_	0.702	0.076	37.706	0.076
LCA_80_	1.499	0.767	44.305	0.767
LCA_120_	2.214	1.550	51.582	1.610
AT_1_	2.511	0.007	48.738	0.002
AT_2_	9.928	0.180	117.397	0.183
AT_4_	21.910	0.117	233.018	0.167

a All calculations are performed with the (CDD-)­THC-based reformulations of the SOS-MP2 and SOS-CC2 methods and employ the cc-pVTZ/cc-pVTZ-RI basis set combination.

**Table 6:** Absolute Δ*E*_THC_ and Δ*E*_CDD_ Errors for the Excitation Energies to the Lowest-Lying Singlet Excited State for a Series of LCA_*n*_ Molecules^a^

	SOS-ADC(2)		SOS-LR-CC2	
	Δ*E*_THC_/meV	Δ*E*_CDD_/meV	Δ*E*_THC_/meV	Δ*E*_CDD_/meV
LCA_40_	13.393	0.002	70.689	0.021
LCA_80_	12.854	0.025	64.477	0.280
LCA_120_	12.731	0.028	53.472	0.230

a All calculations are performed with the (CDD-)­THC-based reformulations of the SOS-ADC(2) and SOS-LR-CC2 methods and employ the cc-pVTZ/cc-pVTZ-RI basis set combination.

For the LCA_*n*_ model systems, the CDD error increases linearly with the system size for ground state calculations using the CDD-THC-based versions of SOS-MP2 or SOS-CC2, as shown in Table 5. In passing, we note that the THC error also increases linearly with the system size, matching observations in previous work.^48^ Moreover, the CDD error is almost identical between the CDD-THC-SOS-MP2 and the CDD-THC-SOS-CC2 method, whereas the THC error is increased going from SOS-MP2 to SOS-CC2, as discussed in the previous section. Overall, it is demonstrated that the CDD error is on the same order as the THC error only for SOS-MP2 energies of the LCA_*n*_ series, while in the remaining cases its contribution to the total CDD-THC error is negligible with a maximal error of 1.6 meV.

Regarding the accuracy for excitation energies, Table 6 demonstrates almost constant CDD errors as well as THC errors for the targeted excited states in the LCA_*n*_ series beyond LCA_80_. This behavior is expected since the investigated electronic excitations are localized on the carboxylic acid group. This is unlike the AT_*n*_ series, in which the locality of the excitation is not preserved for different chain lengths. Overall, the CDD errors are still smaller than the THC errors and are not expected to pose significant challenges in practical applications. Moreover, the CDD error can always be converged to the desired accuracy using tighter thresholds.

Considering the demonstrated influence of the application of the LS-THC approximation together with the CDD approach on the accuracy in Tables 5 and 6, the focus of the remainder of this work is on the computational improvements gained thereby in terms of runtime, asymptotic scaling behavior, and memory requirements.

### 4.2 Computational Efficiency

The sparsity of the ground state one-electron density is closely related to the energy gap between the highest occupied (HOMO) and the lowest unoccupied MO (LUMO). Given a significant HOMO–LUMO gap, asymptotically linear-scaling variants of many common quantum chemistry methods have been proposed.^88,89^ In addition, for excited state calculations the transition density **R** with elements *R*_μν_ in eqs 82 and 83 must be considered, for which the sparsity is related to the locality of the excitation. In order to assess the computational complexity of the newly proposed density-based grid-projection algorithm for LS-THC and the resulting CDD-THC-SOS-LR-CC2 and CDD-THC-SOS-ADC(2) methods, a series of LCA_*n*_ molecules is selected as a best-case scenario. LCA_*n*_ are particularly suitable for the demonstration of the asymptotic time complexity, due to the strong locality of their electronic ground state and first singlet excited state (S_1_), which is mostly localized on the carboxyl group. In addition, the time complexity of the presented methods is discussed for three-dimensional systems, here representatively DNA fragments in the AT_*n*_ series.

#### 4.2.1 Integral-Direct Tensor Hypercontraction

First, the efficiency of the proposed density-based, integral-direct algorithm for the grid-projection of the three-center RI integrals, i.e., for the formation of intermediates **Y** in eq 13, is demonstrated. For this, a comparison between the previously published Cholesky MO-based natural blocking approach (*nat. block.*)^60^ and density-based implementations based on the—in FermiONs++ readily available—J-engine (*J-engine*)^61^ and RI-J (*RI-J*)^62^ methods, as well as the newly proposed method in Section 2.2.3 (*this work*) is drawn in Table 7.

**Table 7:** Wall Time in Hours Required for the Formation of the **Y** ^(oo)^, **Y** ^(vo)^, and **Y** ^(vv)^ Intermediates Based on the Previously Published Natural Blocking (nat. block.)^60^ Approach as Well as Density-Based and Integral-Direct Using the J-Engine (J-engine),^61^ the RI-J Algorithm (RI-J),^62^ and the Proposed Algorithm (this work) From Section 2.2.3 ^a^

		cc-pVDZ				cc-pVTZ			
		nat. block	J-engine	RI-J	this work	nat. block	J-engine	RI-J	this work
**Y** ^(oo)^									
	LCA_20_		<0.1	<0.1	<0.1		0.1	<0.1	<0.1
	LCA_40_		0.1	<0.1	<0.1		0.5	0.1	<0.1
	LCA_80_		1.2	0.1	<0.1		4.9	0.3	<0.1
	LCA_160_		8.7	0.3	<0.1		52.8	1.2	0.4
**Y** ^(vo)^									
	LCA_20_	<0.1	0.1	<0.1	<0.1	<0.1	1.0	0.2	<0.1
	LCA_40_	<0.1	0.7	0.1	<0.1	0.1	9.0	0.8	<0.1
	LCA_80_	<0.1	9.1	0.5	<0.1	0.2	85.2	3.2	0.3
	LCA_160_	0.1	69.8	1.9	0.2	0.7	924.0*	13.1	2.7
**Y** ^(vv)^									
	LCA_20_		0.1	0.1	<0.1		1.3	0.4	<0.1
	LCA_40_		1.3	0.2	<0.1		11.8	1.5	0.1
	LCA_80_		14.5	1.0	<0.1		113.8	5.8	0.4
	LCA_160_		118.0	3.8	0.3		1235.4*	24.1	3.2

a Since the natural blocking approach was optimized for the formation of **Y** in the virtual-occupied subspace, only timings for **Y** ^(vo)^ are reported. Extrapolated values are marked with an asterisk (*).

For the natural blocking algorithm, the recommended settings from ref 60 are used, most notably, attenuated Coulomb RI^41^ with an attenuation strength of ω = 0.1. The latter is required to achieve sparsity in the three-center integrals, which makes this approach efficient. In contrast, all density-based approaches use the regular 1/*r*_12_ metric. As previously reported, the natural blocking-based formation of the **Y** intermediate approaches linear time complexity for the largest molecule sizes and is the fastest among the approaches presented in Table 7, due to the efficiency of the screening in combination with the best-case sparsity of the LCA_*n*_ series. In contrast, the RI-J implementation exhibits quadratic scaling due to the considerable sparsity of the **R** ^(*Q*)^ slices acting as the density matrix in the Coulomb matrix build, which is leveraged by density- and shell-pair-based screening in the RI-J implementation.^62^ Both the J-engine implementation^61^ as well as the algorithm proposed in Section 2.2.3 show cubic time complexity since shell-pair sparsity is leveraged while density-based screening is not employed. It is noteworthy, that, while both variants scale cubically with the system size, the J-engine based method has a significantly higher prefactor, which leads to it being slower than all other variants. In contrast, for the proposed—likewise cubically scaling—approach from Section 2.2.3 the prefactor is low enough to render the method competitive to the natural blocking approach even for very large and untypically sparse molecules like LCAs. In turn, the proposed algorithm is especially advantageous for more realistic, less sparse systems. In order to demonstrate the effectiveness of the algorithm, the time complexity for the formation of the **Y** ^(oo)^, **Y** ^(vo)^, and **Y** ^(vv)^ intermediates for LCA_*n*_ up to LCA_160_ is shown in Figure 1 (left).

**Figure 1:**
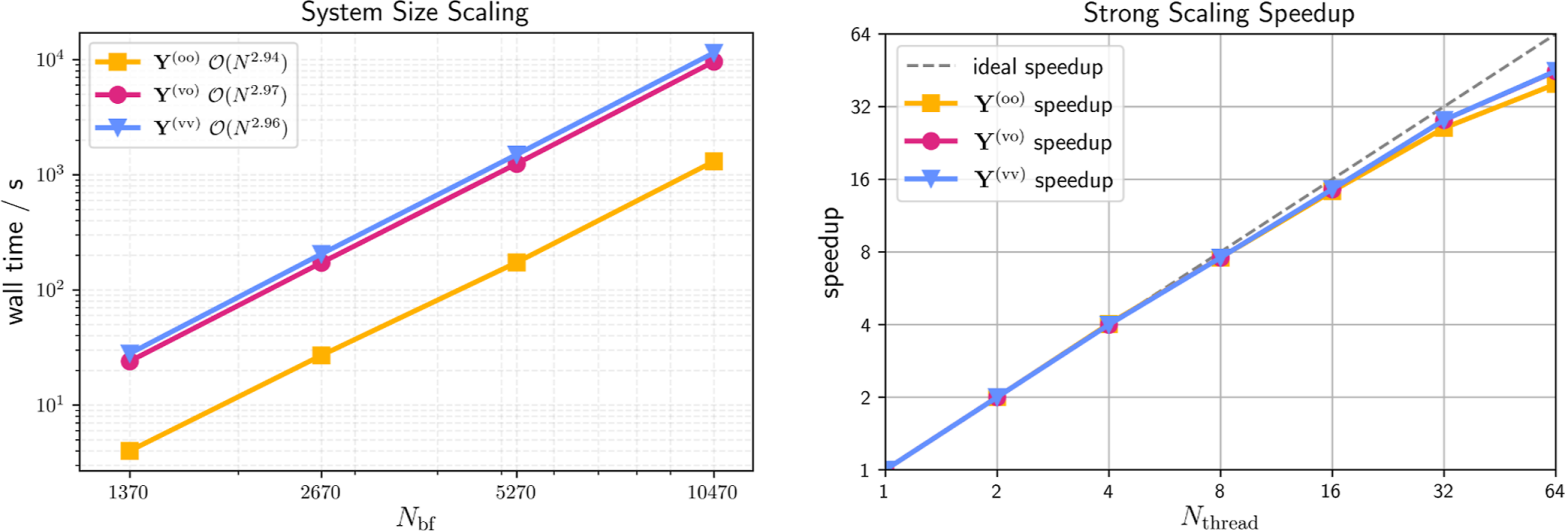
System size scaling (time complexity) presented as a log–log plot (left) and strong scaling speedup (right) of the proposed density-based integral-direct grid-projection algorithm for the cc-pVTZ/cc-pVTZ-RI basis set combination and linear carboxylic acids (LCA_*n*_, *n* ∈ {20, 40, 80, 160}) of increasing size. The strong scaling analysis is performed using LCA_40_ on a single dual-socket compute node comprised of 2 × 32 cores with core performance boost disabled and all cores operating at the base clock speed.

While the asymptotic time complexity is cubic as expected for all MO subspaces (oo, ov, vv), the grid-pruning technique from Matthews^59^ reduces the prefactor for forming the **Y** ^(oo)^ intermediate significantly due to the compactness of the occupied–occupied subspace. Additionally, the strong scaling, i.e., the time-to-solution behavior for the same molecule size but increasing numbers of threads, is assessed and shown in Figure 1 (right). Keeping the individual workload high enough on all processors is key to ideal strong scaling speedup. In this regard, good parallel efficiency and almost ideal strong scaling is observed for up to 32 threads, with only slightly diminishing returns beyond.

In summary, while the proposed grid-projection algorithm has asymptotic cubic time complexity with respect to the systems size, its prefactor is diminutive and it exhibits near-perfect strong scaling speedups. Furthermore, it is competitive to the previously published natural blocking approach while only relying on the shell-pair sparsity of the atomic orbitals, which makes it better suited for nonsparse three-dimensional systems.

#### 4.2.2 THC-SOS-LR-CC2 and THC-SOS-ADC(2)

The computational complexity of the proposed CDD-THC-SOS-LR-CC2 and CDD-THC-SOS-ADC(2) implementations is investigated by taking into account the number of floating-point operations (FLOP) and wall times required to form the singles-manifold CC vector and the matrix-vector product for ground and excited state calculations, respectively. In particular, the number of FLOP and timings for the last iteration of the DIIS procedure is assessed. Note that the FLOP count and timings of LR-SOS-CC2 and SOS-ADC(2) are similar because the steps required to form the matrix-vector products are mostly identical. Figure 2 shows the time complexity measured by the number of FLOP (top) and wall times (bottom) for the ground (left) and the excited state calculations (right) for the MO-RI-SOS-LR-CC2/ADC(2) reference implementations (black) in comparison with the proposed MO/CDD-THC-SOS-LR-CC2/ADC(2) methods (colored) for the LCA_*n*_ series.

**Figure 2:**
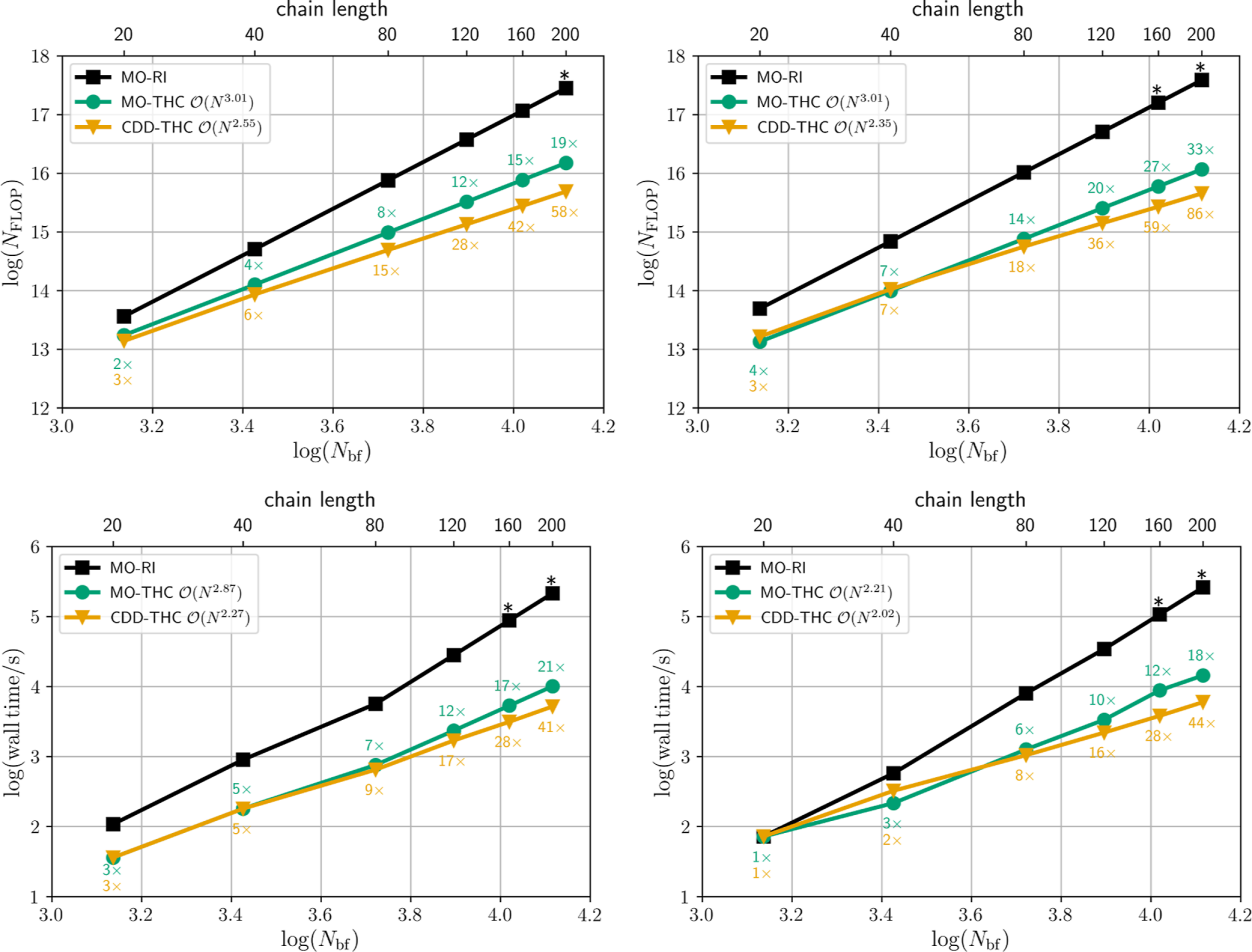
Number of FLOP (top) and wall time (bottom) required to evaluate the SOS-CC2 single amplitudes (left) and the SOS-LR-CC2/SOS-ADC(2) matrix-vector product (right) for a series of LCA_*n*_ (*n* ∈ {20, 40, 80, 120, 160, 200}) molecules using the MO-THC (green) and the CDD-THC-based implementations (orange). Labels represent the FLOP reduction and time speedup compared to the MO-RI-SOS-LR-CC2/ADC(2) implementation (black) and all calculations are performed with the cc-pVTZ/cc-pVTZ-RI basis set combination. Extrapolated values are marked with an asterisk (*).

The MO-based THC-SOS-LR-CC2 and THC-SOS-ADC(2) algorithms allow for a $O\left(N^{3}\right)$ scaling evaluation of the ground state energy and excitation energies with an effort that is ∼19 and ∼33 times smaller than the respective $O\left(N^{4}\right)$ scaling MO-RI reference variant for the largest molecule size considered. Reformulating the equations for the SOS-CC2 single amplitudes in the local Cholesky MO basis, as described in Section 2.3.2 and using sparse linear algebra to solve eq 64 further decreases the computational effort to ∼58-fold. Although the formal scaling behavior is cubic due to steps (e) and (f) in Table 1, a scaling exponent <3 is observed for the ground state CDD-THC-SOS-CC2 method by leveraging the sparsity, as described in Section 2.3.2. For CDD-THC-SOS-LR-CC2 and CDD-THC-SOS-ADC(2) $O\left(N^{2.3}\right)$ scaling is obtained with a ∼86-fold diminution of the effort to evaluate the matrix-vector product in eq 72 for the largest LCA size considered. The observed scaling exponent is slightly larger than the theoretically predicted one reported in Table 2, due to the reduced sparsity of the density matrices within the larger triple-ζ basis. For the smaller cc-pVDZ/cc-pVDZ-RI basis set combination, the observed scaling behavior is closer to the predicted quadratic scaling with an observed time complexity of $O\left(N^{2.1}\right)$ , which is shown in Section S6.1 of the Supporting Information.

A significant reduction of the computational effort is likewise achieved for three-dimensional DNA fragments with up to 12 AT pairs. On the one hand, the cubically scaling MO-based THC-SOS-CC2 and THC-SOS-LR-CC2/ADC(2) methods grant ∼16-fold and ∼26-fold reduction of the effort—relative to the original MO-RI implementation—for computing the contributions to the single amplitudes in eq 24 and the matrix-vector product in eq 37, respectively, as shown in Figure 3. On the other hand, the CDD-THC-SOS-CC2 and CDD-THC-SOS-LR-CC2/ADC(2) reformulations allow for the solution of the singles-manifold cluster equation in eq 64 and the calculation of the matrix-vector product of eq 72 with a computational effort in terms of FLOP that is ∼27 times smaller than their MO-RI variant in both cases. Consequently, the reduction of the number of FLOP translates to a decrease of the wall times up to 24 times for the ground state and up to 17 times for the excitation energy calculation. Despite the observed $O\left(N^{2.5}\right)$ behavior for the calculation of the excitation energies of the AT_*n*_ series, the scaling exponent is expected to decrease toward ∼2 in the asymptotic limit.

**Figure 3:**
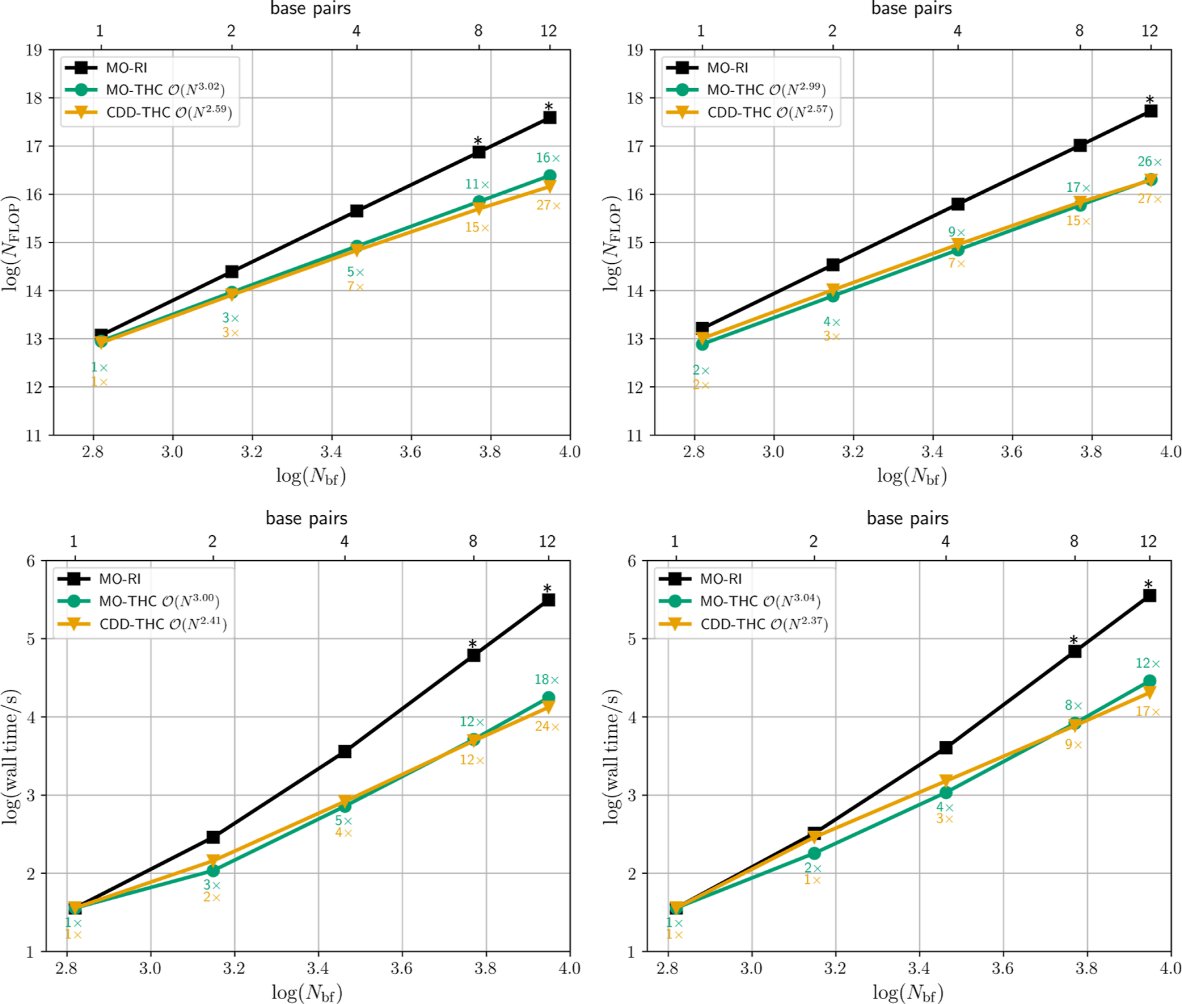
Number of FLOP (top) and wall time (bottom) required to evaluate the SOS-CC2 single amplitudes (left) and the SOS-LR-CC2/SOS-ADC(2) matrix-vector product (right) for a series of AT_*n*_ (*n* ∈ {1, 2, 4, 8, 12}) molecules using the MO-THC (green) and the CDD-THC-based implementations (orange). Labels represent the FLOP reduction and time speedup compared to the MO-RI-SOS-LR-CC2/ADC(2) implementation (black). All calculations are performed with the cc-pVDZ/cc-pVDZ-RI basis set combination. Extrapolated values are marked with an asterisk (*).

Overall, employing CDD-THC-SOS-ADC(2) makes it possible to compute the excitation energy to the two lowest-lying singlet excited states of AT_12_ for the cc-pVDZ/cc-pVDZ-RI basis set combination in ∼7.5 days, for which the most time-consuming steps are shown in Figure 4 with their individual contributions to the overall wall time. Most of the time is spent computing the ADC(2) excitation energies, which requires ∼72% of the total computation time, while only ∼18% of the total computation time is spent evaluating the THC **Γ** matrices. Within the routines to evaluate the **Γ** intermediates, 33% of the time is spent forming the **Y** intermediates using the presented integral-direct routine. Due to the differing pruned grid sizes, the calculation of the **Y** ^(*vv*)^ intermediate for the virtual–virtual subspace contributes roughly 50% of the total time for all **Y** intermediates. Note that the ADC(2) optimization procedure is converged with thresholds of 10^–6^ and 10^–5^ for the excitation vector and the energy, respectively. The guesses for the DIIS procedure are optimized at the ADC(1) level via Davidson optimization, which requires ∼10% of the total time. The SCF procedure, the calculation of the MP2 energy correction, and the evaluation of the ground state intermediates **E** from eq 76 together requires ∼1% of the total time.

**Figure 4:**
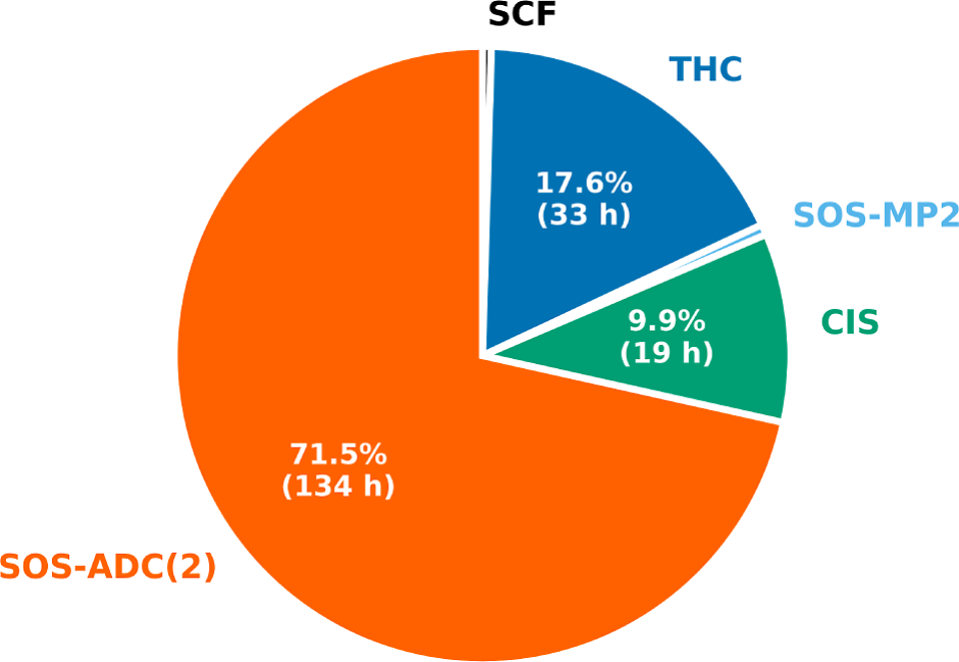
Distribution of the wall time for an illustrative CDD-THC-SOS-ADC(2) calculation of the excitation energies to the two lowest-lying singlet excited states of AT_12_ using the cc-pVDZ/cc-pVDZ-RI basis set combination.

Finally, the memory and storage savings of the THC-based algorithms should be considered which extends the applicability of SOS-ADC(2) and SOS-LR-CC2 to large basis sets and much larger systems, without the need of batching the workload until the ∼1000 atoms scale is reached. Indeed, in the THC-based reformulations only second-order tensors are stored and the space complexity is at most quadratic due to the integral-direct formation of the THC fitting tensors. Moreover, within the CDD-THC-based approach, sparse linear algebra decreases the space complexity of most intermediates, further reducing the memory and storage requirements of the resulting methods. The space complexity is discussed in detail in Section S6.2 of the Supporting Information.

## 5 Conclusion

An efficient reformulation of SOS-CC2 for ground state and SOS-LR-CC2 as well as SOS-ADC(2) for excitation energies is presented. The implementation leverages the THC-factorized representation of the ERI tensor in conjunction with local Cholesky pseudo-MOs from the CDD approach together with block-sparse linear algebra for the resulting tensor contractions. For the expensive formation of the **Y** intermediates, which are the grid-projected three-center RI integrals, an efficient near-perfect strong-scaling integral-direct and density-based method is proposed and implemented. The presented algorithm exhibits cubic time complexity with a very low prefactor, rendering it competitive to the previously published natural blocking approach. Further reduction to quadratic or even linear time complexity would require the use of a local metric, as in the natural blocking approach. In this way, an efficient way to obtain the THC tensors for any orbital space is provided, which is leveraged by THC-based reformulations of SOS-MP2, SOS-(LR-)­CC2, and SOS-ADC(2) for both ground state and excitation energy calculations.

The derived methods show reasonable accuracy for both absolute and relative ground state energies. However, due to the larger fitting space of the (ov|vv)-type integrals required in SOS-CC2, the mean absolute errors are increased from ∼10^–3^ to 10^–2^ eV compared to SOS-MP2. The LS-THC errors are more pronounced for SOS-ADC(2) and SOS-LR-CC2 excitation energies, especially for the singlet states, for which the maximum errors are as large as ∼0.055 eV. Thus, more work regarding the accuracy of the THC approximation especially for (ov|vv)-type integrals is needed in the future. One possible solution is the use of robust THC,^90^ which however entails further computational complexity and is therefore not suitable for the target applications. In addition, a more comprehensive hierarchy of THC specific integration grids would enable finer grained control over the THC error so that more accurate results could be obtained for applications where this is necessary.

In contrast, the additional use of the CDD approach together with the application of sparse linear algebra, does not incur significant further errors, justifying the applicability of the presented methods. In particular, for small molecules the error introduced by the CDD approach and by sparse algebra is virtually zero as no sparsity can be exploited. For large systems the CDD algorithm proved to yield energies close to the MO-based one with an error in the range of ∼10^–5^ to ∼10^–3^ eV, which can be rigorously controlled through the associated thresholds.

Since both SOS-LR-CC2 and SOS-ADC(2) only involve Coulomb-type integral contractions, no higher than second-order tensors have to be formed and all FLOP-intensive contractions can be performed efficiently by matrix–matrix multiplications. Application of the THC approximation yields cubic scaling MO-based algorithms—contrary to the fourth-order scaling RI-based ones—and grants significant speedups for the evaluation of both ground and excitation energies. For ground state calculations, leveraging Cholesky-MOs and sparse algebra further reduces the computational effort and scaling, hence providing additional speedups. However, the scaling behavior of the time-determining steps and the overall scaling of CDD-THC-SOS-MP2 and CDD-THC-SOS-CC2 is reduced to $O\left(N^{2}\right)$ only if the **Z** fitting tensor is used. Contrary to ground state calculations, the use of Cholesky-MOs and sparse algebra reduces the computational scaling of CDD-THC-SOS-ADC(2)/LR-CC2 to quadratic in the asymptotic limit while decreasing the computational effort by about 25-fold for DNA fragments with approximately 800 atoms. The reformulation in the Cholesky-MOs basis and the use of sparse algebra within the LS-THC framework also allow for a significant reduction of the memory demand, which has at most quadratic space complexity. The overall demonstrated computational savings, in both FLOP and wall time required for the solution of the energy expressions, as well as the presented memory savings, render the implemented CDD-THC-based algorithms promising candidates for calculations on large molecular systems.

## Supplementary Material



## Notes

Details about the sparsity-conserving grid pruning, explicit expressions for the THC-SOS-CC2 ground state energy and the THC-SOS-LR-CC2/ADC(2) triplet excited states, working equations for the MO-THC-SOS-LR-CC2 method, detailed results for the benchmark sets, and detailed results for the memory scaling of relevant intermediates (PDF)
