# Supplementary material for: Efficient Low-Scaling Calculation of THC-SOS-LR-CC2 and THC-SOS-ADC(2) Excitation Energies Through Density-Based Integral-Direct Tensor Hypercontraction
Source: J Chem Theory Comput. 2025 May 12;21(10):5083–102. doi: 10.1021/acs.jctc.5c00230 (PMC12120923; doi:10.1021/acs.jctc.5c00230)
Supplement: Supplementary file 1 [file ct5c00230_si_001.pdf]

Supporting Information: Efficient Low-scaling Calculation of THC-SOS-LR-CC2 and THC-SOS-ADC(2) Excitation Energies Through Density-based Integral-direct Tensor Hypercontraction

Filippo Sacchetta,[†,¶] Felix H. Bangerter,[†,¶] Henryk Laqua,[†] and Christian Ochsenfeld*[,†,‡]

†*Chair of Theoretical Chemistry, Department of Chemistry, University of Munich (LMU), D-81377 Munich, Germany*

‡*Max Planck Institute for Solid State Research, D-70569 Stuttgart, Germany*

¶*These authors contributed equally to this work.*

E-mail: christian.ochsenfeld@cup.uni-muenchen.de

Contents

1 Sparsity-conserving Grid Pruning **S-3**

2 Ground State Equations **S-5**

3 Excited State Equations - Triplet States **S-6**

4 Working Equations for the MO-based algorithm **S-8**

5 Detailed Results – Accuracy **S-9**

5.1 Tetraalanine Conformer Benchmark Set S-10

5.2 Thiel Benchmark Set S-14

5.2.1 Ground State S-14

5.2.2 Excited State S-16

5.3 Martínez Benchmark Set S-24

5.3.1 Ground State S-24

5.3.2 Excited State S-26

6 Detailed Results – Computational Efficiency **S-31**

6.1 Time Complexity S-31

6.1.1 THC Grid Comparison S-33

6.1.2 THC $\mathbf{Z}$ vs. $\mathbf{\Gamma}$ Tensor S-34

6.2 Space Complexity S-35

References **S-39**

1 Sparsity-conserving Grid Pruning

Throughout this work the grid pruning technique by Matthews[S1] was used, which serves two purposes: 1) a removal of unnecessary linear combinations of grid points and 2) the solution of the system of linear equations required for forming the final $\mathbf{\Gamma}$ intermediates. Instead of defining novel parent grids from which to prune from, the hand-optimized grid for the cc-pVTZ basis set by Kokkila Schumacher *et al.*[S2] is used as a parent grid. In the following, the influence of the pruning threshold ε, i.e., the rank threshold in the underlying pivoted Cholesky decomposition (PCD), on the accuracy is assessed. Figure S1 shows the behavior of the error (solid lines) of THC-SOS-RI-MP2 compared to SOS-RI-MP2 as well as the growth of the numerical rank (dashed lines), i.e., the resulting number of grid points N_{grid} after pruning, for LCAs up to 160 carbon atoms.

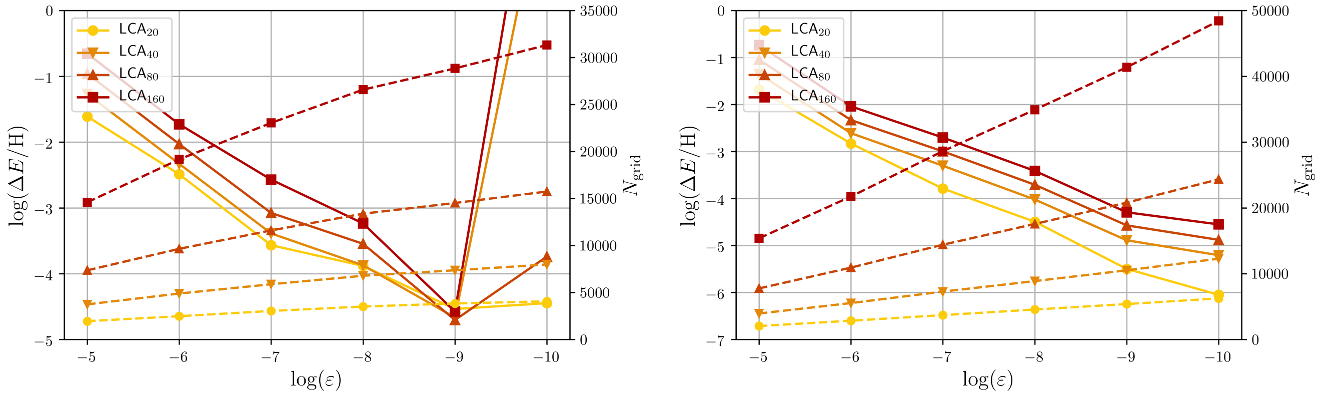

Figure S1: Behavior of the error (solid lines) of THC-SOS-RI-MP2 compared to SOS-RI-MP2 as well as the growth of the numerical rank (dashed lines) with an increasingly tight pruning threshold ε. All calculations were performed with the cc-pVDZ/cc-pVDZ-RI basis set combination and with the hand-optimized grids for the cc-pVDZ (left) and cc-pVTZ (right) basis set as parent grids.

In general a smooth linear reduction of the error and a linear growth of the number of resulting grid points is observed with increasingly tight pruning thresholds. Also, with the hand-optimized grid for the cc-pVDZ basis set serving as a parent grid, the accuracy deteriorates beyond a threshold of 10^{-9}. This has also been observed in previous work by Matthews[S1] and is most likely due to numerical instabilities in the pivoted Cholesky decomposition or the subsequent least-squares fitting. While with the tightest reasonable threshold an acceptable accuracy on the order of 10^{-5} H for absolute energies is achieved for both parent grids, the cc-pVTZ based parent grid was

chosen for all calculations in the main part of this paper because of the more well-behaved error. Additionally note that by comparing the errors between the LCAs of different chain length, it is observed that the error grows linearly with the molecule size, as reported previously.[S1,S3]

One potential drawback of the PCD-based grid pruning is the pivoting, which changes the order of the grid points. To increase sparsity in the one-particle density matrix it is custom to reorder atoms according to the reverse Cuthill–McKee (RCM)[S4] algorithm in order to minimize the bandwidth of the resulting density matrix. Likewise, this reordering also reduces the bandwidth of grid-based intermediates like the grid metric **S**, for which the order of the grid points is predominately determined by the order of the atoms since the constituting grids are atom-centered. However, the pivoting in the PCD causes a reordering of the grid points, which results in unwanted fill-in. To preserve sparsity, all resulting grid points after pruning are assigned to their closest neighboring atom and then ordered according to their parent atom in the order determined by the RCM algorithm. Figure S2 demonstrates the described situation for the THC grid metric tensor **S** for LCA_{80} and highlights the importance of the reordering of the grid points after pivoting.

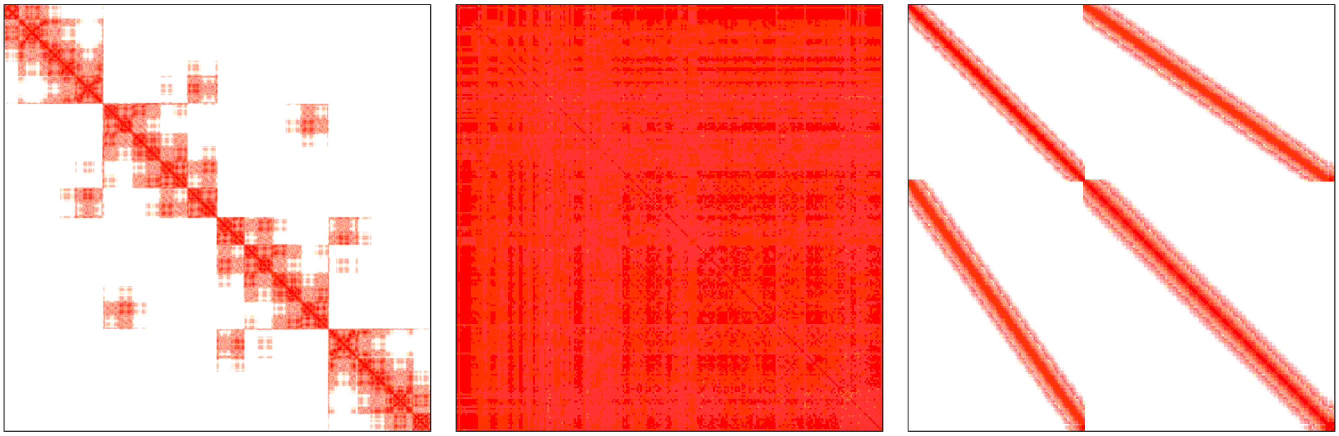

Figure S2: Block sparsity pattern of the THC grid metric **S** in the AO basis before (left) and after pruning (center), as well as after reordering of the grid points (right). Red pixels indicate significant blocks while white pixels represent blocks for which the norm is less than 10^{-10}. All sparsity patterns are obtained from LCA_{80} using the cc-pVDZ basis set and pruning from the hand-optimized cc-pVTZ based grid for the occupied-virtual subspace. Note that the images for the **S** tensor after pruning are scaled up; before pruning **S** was of size 45411×45411 and after pruning of size 22906×22906.

2 Ground State Equations

In the following the working equations of the ground state expressions for the SOS-CC2 singles amplitudes and the SOS-CC2 energy in terms of the THC-based intermediates defined in the main part of this work are given.

$$\begin{aligned}
\Omega_{ai}^{\mathrm{G}} &= -c_{\mathrm{os}} \sum_{\tau} w_\tau \sum_{cbj} \sum_{\alpha\beta} \sum_{PQRS} \hat{X}_c^{P,(\mathrm{vo})} \hat{X}_i^{P,(\mathrm{vo})} \Gamma_\alpha^{P,(\mathrm{vo})} \Gamma_\alpha^{Q,(\mathrm{vo})} \hat{X}_b^{Q,(\mathrm{vo})} \hat{X}_j^{Q,(\mathrm{vo})} e^{-\epsilon_{ci} t_\tau} e^{-\epsilon_{bj} t_\tau} \\
&\cdot \left(X_j^{R,(\mathrm{vo})} X_b^{R,(\mathrm{vo})} \Gamma_\beta^{R,(\mathrm{vo})} \Gamma_\beta^{S,(\mathrm{vv})} \hat{X}_a^{S,(\mathrm{vv})} X_c^{S,(\mathrm{vv})} \right) \\
&= -c_{\mathrm{os}} \sum_{\tau} \sum_{PS} \hat{X}_{i,\tau}^{P,(\mathrm{vo})} \Big[\sum_{\alpha\beta} \sum_{QR} \hat{B}_\tau^{PS,(\mathrm{ovvv})} \Gamma_\alpha^{P,(\mathrm{vo})} \Gamma_\alpha^{Q,(\mathrm{vo})} \hat{A}_\tau^{QR,(\mathrm{vovo})} \hat{B}_\tau^{QR,(\mathrm{vovo})} \Gamma_\beta^{R,(\mathrm{vo})} \Gamma_\beta^{S,(\mathrm{vv})} \Big] \hat{X}_a^{S,(\mathrm{vv})} \\
&= -c_{\mathrm{os}} \sum_{\tau} \sum_{PS} \hat{X}_{i,\tau}^{P,(\mathrm{vo})} M_\tau^{PS,(\mathrm{ovvv})} \hat{X}_a^{S,(\mathrm{vv})} \qquad (1)
\end{aligned}$$

$$\begin{aligned}
\Omega_{ai}^{\mathrm{H}} &= c_{\mathrm{os}} \sum_{\tau} w_\tau \sum_{kbj} \sum_{\alpha\beta} \sum_{PQRS} \hat{X}_a^{P,(\mathrm{vo})} \hat{X}_k^{P,(\mathrm{vo})} \Gamma_\alpha^{P,(\mathrm{vo})} \Gamma_\alpha^{Q,(\mathrm{vo})} \hat{X}_b^{Q,(\mathrm{vo})} \hat{X}_j^{Q,(\mathrm{vo})} e^{-\epsilon_{ak} t_\tau} e^{-\epsilon_{bj} t_\tau} \\
&\cdot \left(X_j^{R,(\mathrm{vo})} X_b^{R,(\mathrm{vo})} \Gamma_\beta^{R,(\mathrm{vo})} \Gamma_\beta^{S,(\mathrm{oo})} X_k^{S,(\mathrm{oo})} \hat{X}_i^{S,(\mathrm{oo})} \right) \\
&= c_{\mathrm{os}} \sum_{\tau} \sum_{PS} \hat{X}_{a,\tau}^{P,(\mathrm{vo})} \Big[\sum_{\alpha\beta} \sum_{QR} \hat{A}_\tau^{PS,(\mathrm{vooo})} \Gamma_\alpha^{P,(\mathrm{vo})} \Gamma_\alpha^{Q,(\mathrm{vo})} \hat{A}_\tau^{QR,(\mathrm{vovo})} \hat{B}_\tau^{QR,(\mathrm{vovo})} \Gamma_\beta^{R,(\mathrm{vo})} \Gamma_\beta^{S,(\mathrm{vv})} \Big] \hat{X}_i^{S,(\mathrm{oo})} \\
&= c_{\mathrm{os}} \sum_{\tau} \sum_{PS} \hat{X}_{a,\tau}^{P,(\mathrm{vo})} N_\tau^{PS,(\mathrm{vooo})} \hat{X}_i^{S,(\mathrm{vv})} \qquad (2)
\end{aligned}$$

$$\begin{aligned}
E^{\text{SOS-CC2}} &= c_{\mathrm{os}} \sum_{aibj} \sum_{RS} \sum_{\alpha} t_{ai} t_{bj} X_a^{R,(\mathrm{vo})} X_i^{R,(\mathrm{vo})} \Gamma_\alpha^{R,(\mathrm{vo})} \Gamma_\alpha^{S,(\mathrm{vo})} X_b^{S,(\mathrm{vo})} X_j^{S,(\mathrm{vo})} \\
&- c_{\mathrm{os}} \sum_{\tau} w_\tau \sum_{aibj} \sum_{PQRS} \sum_{\alpha\beta} \hat{X}_a^{P,(\mathrm{vo})} \hat{X}_i^{P,(\mathrm{vo})} \Gamma_\alpha^{P,(\mathrm{vo})} \Gamma_\alpha^{Q,(\mathrm{vo})} \hat{X}_b^{Q,(\mathrm{vo})} \hat{X}_j^{Q,(\mathrm{vo})} e^{-\epsilon_{ai} t_\tau} e^{-\epsilon_{bj} t_\tau} \\
&\cdot \left(\hat{X}_a^{R,(\mathrm{vo})} \hat{X}_i^{R,(\mathrm{vo})} \Gamma_\beta^{R,(\mathrm{vo})} \Gamma_\beta^{S,(\mathrm{vo})} \hat{X}_b^{S,(\mathrm{vo})} \hat{X}_j^{S,(\mathrm{vo})} \right) \\
&= c_{\mathrm{os}} \sum_{aibj} \sum_{RS} \sum_{\alpha} t_{ai} t_{bj} X_a^{R,(\mathrm{vo})} X_i^{R,(\mathrm{vo})} \Gamma_\alpha^{R,(\mathrm{vo})} \Gamma_\alpha^{S,(\mathrm{vo})} X_b^{S,(\mathrm{vo})} X_j^{S,(\mathrm{vo})} \\
&- c_{\mathrm{os}} \sum_{\tau} w_\tau \sum_{\alpha\beta} \hat{C}_\tau^{\alpha\beta,(\mathrm{ovov})} \hat{C}_\tau^{\alpha\beta,(\mathrm{ovov})} \qquad (3)
\end{aligned}$$

3 Excited State Equations - Triplet States

The matrix-vector products for triplet (T) states are given by

$$\begin{aligned}
\sigma_{ai}^{\text{SOS-CC2,(T)}} &= \hat{F}_{ab}R_{bi} - R_{aj}\hat{F}_{ji} - \sum_{ck}(ac\hat{|}ki)R_{ck} - c_{\text{os}}\sum_{b}E_{ab}R_{bi} - c_{\text{os}}\sum_{j}R_{aj}E_{ji} \\
&+ \sigma_{ai}^{\text{G,(T)}} + \sigma_{ai}^{\text{H,(T)}} + \sigma_{ai}^{\text{I,(T)}} \\
\sigma_{ai}^{\text{SOS-ADC(2),(T)}} &= (\epsilon_a - \epsilon_i)R_{ai} - \sum_{ck}(ac|ki)R_{ck} - c_{\text{os}}\sum_{b}E_{ab}R_{bi} - c_{\text{os}}\sum_{j}R_{aj}E_{ji} \\
&+ \sigma_{ai}^{\text{G,(T)}} + \sigma_{ai}^{\text{H,(T)}} + \sigma_{ai}^{\text{I,(T)}} \qquad (4)
\end{aligned}$$

The derivation of the singlet equations is discussed in the main part of this work. In order to derive the equations for the triplet states it is necessary to consider the following spin-symmetry relationships for singlet

$$R_{a_\alpha i_\alpha} = R_{a_\beta i_\beta} \qquad (5)$$

and triplet states

$$R_{a_\alpha i_\alpha} = -R_{a_\beta i_\beta} \qquad (6)$$

where α, β here are the spin of the electrons. Accordingly, the intermediates of the matrix-vector product are given by

$$
\begin{aligned}
\bar{M}_{\tau}^{PS,(\text{ovvv})} &= -\bar{M}_{\tau,(1)}^{PS,(\text{ovvv})} + \bar{M}_{\tau,(2)}^{PS,(\text{ovvv})} \\
&= -\hat{B}_{\tau}^{PS,(\text{ovvv})}\bar{D}_{\tau}^{PS,(\text{ovvv})} + \bar{B}_{\tau}^{PS,(\text{ovvv})}\hat{D}_{\tau}^{PS,(\text{ovvv})} \qquad (7)
\end{aligned}
$$

$$
\begin{aligned}
\bar{N}_{\tau}^{PS,(\text{vooo})} &= -\bar{N}_{\tau,(1)}^{PS,(\text{vooo})} + \bar{N}_{\tau,(2)}^{PS,(\text{vooo})} \\
&= -\hat{A}_{\tau}^{PS,(\text{vooo})}\bar{D}_{\tau}^{PS,(\text{vooo})} + \bar{A}_{\tau}^{PS,(\text{vooo})}\hat{D}_{\tau}^{PS,(\text{vooo})} \qquad (8)
\end{aligned}
$$

$$
\begin{aligned}
\sigma_{ai}^{\text{I,SOS-CC2,(T)}} &= +c_{\text{os}}\sum_{bj} t_{aibj}^{\text{os}}\bar{F}_{jb} + \sum_{bj} c_{\text{os}} R_{ij}^{ab}(\bar{\omega})\hat{F}_{jb} \\
&= +c_{\text{os}}\sum_{\tau}\sum_{P}\hat{X}_{a,\tau}^{P,(\text{vo})}\hat{X}_{i,\tau}^{P,(\text{vo})}\bar{I}_{\tau,(1)}^{P,(\text{vo})} \\
&\quad - c_{\text{os}}\sum_{\tau} e^{\bar{\omega}t_{\tau}}\sum_{P}\left[\bar{X}_{a,\tau}^{P,(\text{vo})}\hat{X}_{i,\tau}^{P,(\text{vo})} + \hat{X}_{a,\tau}^{P,(\text{vo})}\bar{X}_{i,\tau}^{P,(\text{vo})}\right]\hat{I}_{\tau}^{P,(\text{vo})} \qquad (9)
\end{aligned}
$$

$$
\begin{aligned}
\sigma_{ai}^{\text{I,SOS-ADC(2),(T)}} &= +\frac{c_{\text{os}}}{2}\sum_{bj} t_{aibj}^{\text{os}}\bar{F}_{jb} - \frac{c_{\text{os}}}{2}\sum_{bj}(ib|ja)\Big[\sum_{ck} t_{ckbj}^{\text{os}} R_{ck}\Big] \\
&= +\frac{c_{\text{os}}}{2}\sum_{\tau}\sum_{P} X_{a,\tau}^{P,(\text{vo})} X_{i,\tau}^{P,(\text{vo})}\bar{I}_{\tau,(1)}^{P,(\text{vo})} - \frac{c_{\text{os}}}{2}\sum_{bj}(ib|ja)\bar{I}_{bj} \qquad (10)
\end{aligned}
$$

where for triplet states

$$
\bar{F}_{ib} = -(jc\hat{|}kb)R_{ck} \qquad (11)
$$

4 Working Equations for the MO-based algorithm

Table S1: Working equations of the intermediates for the solution of eq. 24 and 29 in the main part of this work. Notice that, for SOS-MP2 calculations, $t_{ai} = 0$ and only eq. (a)-(d) need to be solved.

	Intermediate	Formal Scaling
(a)	$\hat{A}_{\tau}^{QR,(\text{vovo})} = \hat{X}_{i,\tau}^{Q,(\text{vo})} X_{i}^{R,(\text{vo})}$	$N_{\text{occ}} N_{\text{grid-ov}}^{2}$
(b)	$\hat{B}_{\tau}^{QR,(\text{vovo})} = \hat{X}_{a,\tau}^{Q,(\text{vo})} X_{a}^{R,(\text{vo})}$	$N_{\text{virt}} N_{\text{grid-ov}}^{2}$
(c)	$\hat{A}_{\tau}^{PS,(\text{vooo})} = \hat{X}_{i,\tau}^{P,(\text{vo})} X_{i}^{S,(\text{oo})}$	$N_{\text{occ}} N_{\text{grid-ov}} N_{\text{grid-oo}}$
(d)	$\hat{B}_{\tau}^{PS,(\text{ovvv})} = \hat{X}_{a,\tau}^{P,(\text{vo})} X_{a}^{S,(\text{vv})}$	$N_{\text{virt}} N_{\text{grid-ov}} N_{\text{grid-vv}}$
(e)	$\hat{Y}_{a}^{S,(\text{oo})} = \hat{X}_{a,\tau}^{P,(\text{vo})} \hat{N}_{\tau}^{PS,(\text{vooo})}$	$N_{\text{virt}} N_{\text{grid-ov}} N_{\text{grid-oo}}$
(f)	$\hat{Y}_{i}^{S,(\text{vv})} = \hat{X}_{i,\tau}^{P,(\text{vo})} \hat{M}_{\tau}^{PS,(\text{ovvv})}$	$N_{\text{occ}} N_{\text{grid-ov}} N_{\text{grid-vv}}$
(g)	$\hat{I}_{j,\tau}^{P,(\text{ov})} = \hat{X}_{b,\tau}^{P,(\text{vo})} \hat{F}_{jb}$	$N_{\text{virt}} N_{\text{occ}} N_{\text{grid-ov}}$
(h)	$\hat{n}_{\tau}^{P,(\text{ov})} = \hat{X}_{j,\tau}^{P,(\text{vo})} \hat{I}_{j,\tau}^{P,(\text{ov})}$	$N_{\text{occ}} N_{\text{grid-ov}}$

Table S2: Working equations of the intermediates for the solution of the MO-THC-SOS-LR-CC2 excitation energy calculations.

	Intermediate	Formal Scaling
(a)	$\bar{A}_{\tau}^{QR,(\text{vovo})} = \bar{X}_{i,\tau}^{Q,(\text{vo})} X_{i}^{R,(\text{vo})}$	$N_{\text{occ}} N_{\text{grid-ov}}^{2}$
(b)	$\bar{B}_{\tau}^{QR,(\text{vovo})} = \bar{X}_{a,\tau}^{Q,(\text{vo})} X_{a}^{R,(\text{vo})}$	$N_{\text{virt}} N_{\text{grid-ov}}^{2}$
(c)	$\bar{A}_{\tau}^{PS,(\text{vooo})} = \bar{X}_{i,\tau}^{P,(\text{vo})} X_{i}^{S,(\text{oo})}$	$N_{\text{occ}} N_{\text{grid-ov}} N_{\text{grid-oo}}$
(d)	$\bar{B}_{\tau}^{PS,(\text{ovvv})} = \bar{X}_{a,\tau}^{P,(\text{vo})} X_{a}^{S,(\text{vv})}$	$N_{\text{virt}} N_{\text{grid-ov}} N_{\text{grid-vv}}$
(e)	$\bar{Y}_{a}^{S,(\text{oo})} = \hat{X}_{a,\tau}^{P,(\text{vo})} \hat{N}_{\tau}^{PS,(\text{vooo})}$	$N_{\text{virt}} N_{\text{grid-ov}} N_{\text{grid-oo}}$
(f)	$\bar{Y}_{i}^{S,(\text{vv})} = \hat{X}_{i,\tau}^{P,(\text{vo})} \hat{M}_{\tau}^{PS,(\text{ovvv})}$	$N_{\text{occ}} N_{\text{grid-ov}} N_{\text{grid-vv}}$

5 Detailed Results – Accuracy

In the following, detailed results for the accuracy of the proposed MO/CDD-THC-SOS variants of the MP2, CC2, LR-CC2, and ADC(2) methods are given. Section 5.1 summarizes the ground state energies of 27 tetraalanine conformers[S5] as obtained with the THC-SOS-MP2 and the THC-SOS-CC2 method in Tables S3 and S5, respectively. The resulting relative energies and the associated method errors are given in Tables S4 and S6. Additionally, the performance of the proposed methods is assessed for the Thiel benchmark set[S6] in Section 5.2 for both ground and excitation energies in Sections 5.2.1 and 5.2.2, respectively. The ground state energies relative to the MO-RI-SOS-based reference implementation are given in Table S7 for MP2 and in Table S8 for CC2. For the comparison of the excitation energies both the three lowest-lying singlet (S) and triplet states (T) are considered in Tables S9–S12 for ADC(2) and LR-CC2. Likewise, the same analysis is shown for the benchmark set used by Hohenstein et al.[S7] in their work on THC-EOM-CC2 in Tables S13–S18 in Section 5.3.

5.1 Tetraalanine Conformer Benchmark Set

Table S3: Detailed results for the ground state energies of the 27 conformers in the tetraalanine conformer benchmark set.[S5] Errors of the MO- and CDD-based THC-SOS-MP2 methods are reported as deviations from the RI-based reference implementation. All calculations are performed with the cc-pVTZ/cc-pVTZ-RI basis set combination.

conformer	E_{RI} / H	E_{THC} / H	ΔE_{THC} / H	$E_{\text{CDD-THC}}$ / H	$\Delta E_{\text{CDD-THC}}$ / H
1	−3.667 064	−3.667 049	1.52×10^{-5}	−3.667 048	1.5×10^{-5}
2	−3.668 537	−3.668 520	1.71×10^{-5}	−3.668 520	1.7×10^{-5}
3	−3.674 690	−3.674 671	1.92×10^{-5}	−3.674 671	1.9×10^{-5}
4	−3.668 832	−3.668 819	1.38×10^{-5}	−3.668 818	1.4×10^{-5}
5	−3.668 713	−3.668 699	1.42×10^{-5}	−3.668 699	1.4×10^{-5}
6	−3.672 337	−3.672 329	7.37×10^{-6}	−3.672 329	7.4×10^{-6}
7	−3.674 497	−3.674 482	1.55×10^{-5}	−3.674 482	1.6×10^{-5}
8	−3.674 896	−3.674 873	2.25×10^{-5}	−3.674 873	2.3×10^{-5}
9	−3.674 377	−3.674 362	1.51×10^{-5}	−3.674 362	1.5×10^{-5}
10	−3.675 256	−3.675 246	1.05×10^{-5}	−3.675 246	1.1×10^{-5}
11	−3.675 327	−3.675 304	2.24×10^{-5}	−3.675 304	2.2×10^{-5}
12	−3.675 834	−3.675 814	1.99×10^{-5}	−3.675 814	2.0×10^{-5}
13	−3.670 409	−3.670 389	1.99×10^{-5}	−3.670 389	2.0×10^{-5}
14	−3.670 941	−3.670 924	1.70×10^{-5}	−3.670 924	1.7×10^{-5}
15	−3.676 361	−3.676 345	1.56×10^{-5}	−3.676 345	1.6×10^{-5}
16	−3.673 609	−3.673 596	1.29×10^{-5}	−3.673 596	1.3×10^{-5}
17	−3.675 224	−3.675 213	1.09×10^{-5}	−3.675 213	1.1×10^{-5}
18	−3.672 219	−3.672 201	1.81×10^{-5}	−3.672 201	1.8×10^{-5}
19	−3.672 817	−3.672 805	1.23×10^{-5}	−3.672 805	1.2×10^{-5}
20	−3.672 345	−3.672 322	2.34×10^{-5}	−3.672 322	2.4×10^{-5}
21	−3.673 284	−3.673 269	1.51×10^{-5}	−3.673 269	1.5×10^{-5}
22	−3.674 540	−3.674 531	9.07×10^{-6}	−3.674 530	9.2×10^{-6}
23	−3.674 351	−3.674 339	1.21×10^{-5}	−3.674 339	1.2×10^{-5}
24	−3.673 938	−3.673 932	6.20×10^{-6}	−3.673 932	6.3×10^{-6}
25	−3.672 778	−3.672 760	1.77×10^{-5}	−3.672 760	1.8×10^{-5}
26	−3.618 773	−3.618 761	1.15×10^{-5}	−3.618 761	1.2×10^{-5}
27	−3.673 077	−3.673 052	2.46×10^{-5}	−3.673 052	2.5×10^{-5}

Table S4: Detailed results for the relative energies of the 27 conformers in the tetraalanine conformer benchmark set.[S5] Errors of the MO- and CDD-based THC-SOS-MP2 methods are reported as deviations from the RI-based reference implementation. All calculations are performed with the cc-pVTZ/cc-pVTZ-RI basis set combination.

conformer	E_{RI} / H	E_{THC} / H	ΔE_{THC} / H	$E_{\text{CDD-THC}}$ / H	$\Delta E_{\text{CDD-THC}}$ / H
1	0.001 473	0.001 471	1.9×10^{-6}	0.001 471	1.9×10^{-6}
2	0.006 153	0.006 151	2.2×10^{-6}	0.006 151	2.0×10^{-6}
3	−0.005 858	−0.005 852	5.4×10^{-6}	−0.005 853	5.2×10^{-6}
4	−0.000 119	−0.000 119	4.0×10^{-7}	−0.000 119	3.7×10^{-7}
5	0.003 623	0.003 630	6.9×10^{-6}	0.003 630	7.0×10^{-6}
6	0.002 160	0.002 152	8.2×10^{-6}	0.002 152	8.2×10^{-6}
7	0.000 399	0.000 392	7.0×10^{-6}	0.000 392	7.0×10^{-6}
8	−0.000 519	−0.000 511	7.4×10^{-6}	−0.000 511	7.4×10^{-6}
9	0.000 879	0.000 884	4.6×10^{-6}	0.000 884	4.5×10^{-6}
10	0.000 070	0.000 058	1.2×10^{-5}	0.000 059	1.2×10^{-5}
11	0.000 507	0.000 510	2.5×10^{-6}	0.000 510	2.5×10^{-6}
12	−0.005 425	−0.005 425	4.7×10^{-8}	−0.005 425	2.0×10^{-7}
13	0.000 532	0.000 535	2.9×10^{-6}	0.000 535	3.0×10^{-6}
14	0.005 420	0.005 421	1.4×10^{-6}	0.005 421	1.5×10^{-6}
15	−0.002 752	−0.002 749	2.7×10^{-6}	−0.002 749	2.7×10^{-6}
16	0.001 615	0.001 617	2.0×10^{-6}	0.001 617	2.0×10^{-6}
17	−0.003 005	−0.003 012	7.2×10^{-6}	−0.003 012	7.4×10^{-6}
18	0.000 598	0.000 603	5.8×10^{-6}	0.000 604	5.9×10^{-6}
19	−0.000 472	−0.000 483	1.1×10^{-5}	−0.000 483	1.1×10^{-5}
20	0.000 939	0.000 947	8.2×10^{-6}	0.000 947	8.4×10^{-6}
21	0.001 256	0.001 262	6.1×10^{-6}	0.001 262	6.0×10^{-6}
22	−0.000 188	−0.000 191	3.1×10^{-6}	−0.000 191	3.0×10^{-6}
23	−0.000 413	−0.000 407	5.9×10^{-6}	−0.000 407	5.9×10^{-6}
24	−0.001 161	−0.001 172	1.2×10^{-5}	−0.001 172	1.2×10^{-5}
25	−0.054 005	−0.053 999	6.2×10^{-6}	−0.053 999	6.1×10^{-6}
26	0.054 304	0.054 291	1.3×10^{-5}	0.054 291	1.3×10^{-5}
27	−0.006 013	−0.006 004	9.4×10^{-6}	−0.006 004	9.3×10^{-6}

Table S5: Detailed results for the ground state energies of the 27 conformers in the tetraalanine conformer benchmark set.[S5] Errors of the MO- and CDD-based THC-SOS-CC2 methods are reported as deviations from the RI-based reference implementation. All calculations are performed with the cc-pVTZ/cc-pVTZ-RI basis set combination.

conformer	E_{RI} / H	E_{THC} / H	ΔE_{THC} / H	$E_{\mathrm{CDD\text{-}THC}}$ / H	$\Delta E_{\mathrm{CDD\text{-}THC}}$ / H
1	−3.704 518	−3.703 223	1.3×10^{-3}	−3.703 222	1.3×10^{-3}
2	−3.706 243	−3.704 903	1.3×10^{-3}	−3.704 903	1.3×10^{-3}
3	−3.713 146	−3.711 714	1.4×10^{-3}	−3.711 714	1.4×10^{-3}
4	−3.706 538	−3.705 272	1.3×10^{-3}	−3.705 272	1.3×10^{-3}
5	−3.706 265	−3.704 960	1.3×10^{-3}	−3.704 960	1.3×10^{-3}
6	−3.710 283	−3.708 942	1.3×10^{-3}	−3.708 942	1.3×10^{-3}
7	−3.712 606	−3.711 233	1.4×10^{-3}	−3.711 233	1.4×10^{-3}
8	−3.713 154	−3.711 794	1.4×10^{-3}	−3.711 794	1.4×10^{-3}
9	−3.712 665	−3.711 314	1.4×10^{-3}	−3.711 314	1.4×10^{-3}
10	−3.713 348	−3.712 029	1.3×10^{-3}	−3.712 029	1.3×10^{-3}
11	−3.713 740	−3.712 329	1.4×10^{-3}	−3.712 329	1.4×10^{-3}
12	−3.714 224	−3.712 841	1.4×10^{-3}	−3.712 841	1.4×10^{-3}
13	−3.708 323	−3.706 938	1.4×10^{-3}	−3.706 937	1.4×10^{-3}
14	−3.708 678	−3.707 366	1.3×10^{-3}	−3.707 366	1.3×10^{-3}
15	−3.714 620	−3.713 261	1.4×10^{-3}	−3.713 261	1.4×10^{-3}
16	−3.711 825	−3.710 421	1.4×10^{-3}	−3.710 421	1.4×10^{-3}
17	−3.713 875	−3.712 424	1.5×10^{-3}	−3.712 424	1.5×10^{-3}
18	−3.710 408	−3.709 053	1.4×10^{-3}	−3.709 053	1.4×10^{-3}
19	−3.711 024	−3.709 635	1.4×10^{-3}	−3.709 635	1.4×10^{-3}
20	−3.710 394	−3.709 030	1.4×10^{-3}	−3.709 030	1.4×10^{-3}
21	−3.711 490	−3.710 084	1.4×10^{-3}	−3.710 084	1.4×10^{-3}
22	−3.712 674	−3.711 332	1.3×10^{-3}	−3.711 332	1.3×10^{-3}
23	−3.712 438	−3.711 118	1.3×10^{-3}	−3.711 118	1.3×10^{-3}
24	−3.711 950	−3.710 596	1.4×10^{-3}	−3.710 596	1.4×10^{-3}
25	−3.710 823	−3.709 507	1.3×10^{-3}	−3.709 507	1.3×10^{-3}
26	−3.665 611	−3.665 864	2.5×10^{-4}	−3.665 864	2.5×10^{-4}
27	−3.711 086	−3.709 761	1.3×10^{-3}	−3.709 760	1.3×10^{-3}

Table S6: Detailed results for the relative energies of the 27 conformers in the tetraalanine conformer benchmark set.[S5] Errors of the MO- and CDD-based THC-SOS-CC2 methods are reported as deviations from the RI-based reference implementation. All calculations are performed with the cc-pVTZ/cc-pVTZ-RI basis set combination.

conformer	E_{RI} / H	E_{THC} / H	ΔE_{THC} / H	$E_{\text{CDD-THC}}$ / H	$\Delta E_{\text{CDD-THC}}$ / H
1	0.001 725	0.001 680	4.5×10^{-5}	0.001 680	4.5×10^{-5}
2	0.006 903	0.006 811	9.2×10^{-5}	0.006 811	9.2×10^{-5}
3	−0.006 608	−0.006 442	1.7×10^{-4}	−0.006 442	1.7×10^{-4}
4	−0.000 273	−0.000 312	3.9×10^{-5}	−0.000 312	3.9×10^{-5}
5	0.004 018	0.003 982	3.6×10^{-5}	0.003 982	3.6×10^{-5}
6	0.002 323	0.002 292	3.1×10^{-5}	0.002 292	3.1×10^{-5}
7	0.000 548	0.000 561	1.3×10^{-5}	0.000 561	1.3×10^{-5}
8	−0.000 489	−0.000 480	8.7×10^{-6}	−0.000 480	8.7×10^{-6}
9	0.000 683	0.000 715	3.2×10^{-5}	0.000 715	3.2×10^{-5}
10	0.000 392	0.000 299	9.3×10^{-5}	0.000 300	9.3×10^{-5}
11	0.000 484	0.000 513	2.9×10^{-5}	0.000 513	2.9×10^{-5}
12	−0.005 901	−0.005 904	2.9×10^{-6}	−0.005 904	3.1×10^{-6}
13	0.000 355	0.000 428	7.3×10^{-5}	0.000 428	7.3×10^{-5}
14	0.005 942	0.005 895	4.7×10^{-5}	0.005 895	4.7×10^{-5}
15	−0.002 795	−0.002 840	4.5×10^{-5}	−0.002 840	4.5×10^{-5}
16	0.002 049	0.002 004	4.6×10^{-5}	0.002 004	4.6×10^{-5}
17	−0.003 466	−0.003 372	9.5×10^{-5}	−0.003 372	9.5×10^{-5}
18	0.000 615	0.000 583	3.3×10^{-5}	0.000 583	3.3×10^{-5}
19	−0.000 630	−0.000 606	2.4×10^{-5}	−0.000 606	2.4×10^{-5}
20	0.001 097	0.001 054	4.3×10^{-5}	0.001 054	4.3×10^{-5}
21	0.001 184	0.001 248	6.5×10^{-5}	0.001 248	6.4×10^{-5}
22	−0.000 237	−0.000 214	2.2×10^{-5}	−0.000 214	2.2×10^{-5}
23	−0.000 488	−0.000 522	3.4×10^{-5}	−0.000 522	3.4×10^{-5}
24	−0.001 128	−0.001 090	3.8×10^{-5}	−0.001 090	3.8×10^{-5}
25	−0.045 211	−0.043 643	1.6×10^{-3}	−0.043 643	1.6×10^{-3}
26	0.045 474	0.043 896	1.6×10^{-3}	0.043 897	1.6×10^{-3}
27	−0.006 568	−0.006 538	3.0×10^{-5}	−0.006 538	3.0×10^{-5}

5.2 Thiel Benchmark Set

5.2.1 Ground State

Table S7: Detailed results for the ground state energies of the molecules in the benchmark set from Ref. S6. Errors of the MO- and CDD-based THC-SOS-MP2 methods are reported as deviations from the RI-based reference implementation. All calculations are performed with the cc-pVTZ/cc-pVTZ-RI basis set combination.

molecule	E_{RI} / H	E_{THC} / H	ΔE_{THC} / H	$E_{\text{CDD-THC}}$ / H	$\Delta E_{\text{CDD-THC}}$ / H
ethene	−0.346 126	−0.346 126	1.7×10^{-7}	−0.346 126	1.7×10^{-7}
butadiene	−0.658 266	−0.658 268	1.3×10^{-6}	−0.658 268	1.3×10^{-6}
hexatriene	−0.971 382	−0.971 378	4.0×10^{-6}	−0.971 378	4.0×10^{-6}
octatetraene	−1.284 868	−1.284 862	5.5×10^{-6}	−1.284 862	5.5×10^{-6}
cyclopropene	−0.489 211	−0.489 212	3.3×10^{-7}	−0.489 212	3.3×10^{-7}
cyclopentadiene	−0.801 368	−0.801 368	4.8×10^{-7}	−0.801 368	4.8×10^{-7}
norbornadiene	−1.129 467	−1.129 454	1.3×10^{-5}	−1.129 454	1.3×10^{-5}
benzene	−0.932 969	−0.932 963	6.0×10^{-6}	−0.932 963	6.0×10^{-6}
naphthalene	−1.526 318	−1.526 281	3.8×10^{-5}	−1.526 281	3.8×10^{-5}
furan	−0.849 522	−0.849 526	4.6×10^{-6}	−0.849 526	4.6×10^{-6}
pyrrole	−0.830 485	−0.830 483	1.8×10^{-6}	−0.830 483	1.8×10^{-6}
imidazole	−0.857 685	−0.857 677	7.2×10^{-6}	−0.857 677	7.2×10^{-6}
pyridine	−0.960 355	−0.960 343	1.2×10^{-5}	−0.960 343	1.2×10^{-5}
pyrazine	−0.990 077	−0.990 059	1.9×10^{-5}	−0.990 059	1.9×10^{-5}
pyrimidine	−0.985 217	−0.985 200	1.7×10^{-5}	−0.985 200	1.7×10^{-5}
pyridazine	−0.994 744	−0.994 716	2.8×10^{-5}	−0.994 716	2.8×10^{-5}
triazine	−1.007 924	−1.007 900	2.4×10^{-5}	−1.007 900	2.4×10^{-5}
tetrazine	−1.056 582	−1.056 545	3.8×10^{-5}	−1.056 545	3.8×10^{-5}
formaldehyde	−0.394 740	−0.394 741	6.3×10^{-7}	−0.394 741	6.3×10^{-7}
acetone	−0.742 969	−0.742 970	1.1×10^{-6}	−0.742 970	1.1×10^{-6}
benzoquinone	−1.342 556	−1.342 550	6.0×10^{-6}	−1.342 550	6.0×10^{-6}
formamide	−0.593 511	−0.593 510	6.2×10^{-7}	−0.593 510	6.2×10^{-7}
acetamide	−0.767 468	−0.767 466	1.7×10^{-6}	−0.767 466	1.7×10^{-6}
propanamide	−0.944 016	−0.944 017	4.0×10^{-7}	−0.944 017	4.0×10^{-7}
adenine	−1.698 357	−1.698 320	3.7×10^{-5}	−1.698 320	3.7×10^{-5}
cytosine	−1.404 395	−1.404 366	2.9×10^{-5}	−1.404 366	2.9×10^{-5}
thymine	−1.602 804	−1.602 792	1.2×10^{-5}	−1.602 792	1.2×10^{-5}
uracil	−1.424 320	−1.424 312	8.1×10^{-6}	−1.424 312	8.1×10^{-6}

Table S8: Detailed results for the ground state energies of the molecules in the benchmark set from Ref. S6. Errors of the MO- and CDD-based THC-SOS-CC2 methods are reported as deviations from the RI-based reference implementation. All calculations are performed with the cc-pVTZ/cc-pVTZ-RI basis set combination.

molecule	E_{RI} / H	E_{THC} / H	ΔE_{THC} / H	$E_{\text{CDD-THC}}$ / H	$\Delta E_{\text{CDD-THC}}$ / H
ethene	−0.348 739	−0.348 686	5.3×10^{-5}	−0.348 686	5.3×10^{-5}
butadiene	−0.663 446	−0.663 380	6.6×10^{-5}	−0.663 380	6.6×10^{-5}
hexatriene	−0.979 143	−0.979 049	9.4×10^{-5}	−0.979 049	9.4×10^{-5}
octatetraene	−1.295 213	−1.295 095	1.2×10^{-4}	−1.295 095	1.2×10^{-4}
cyclopropene	−0.492 823	−0.492 790	3.3×10^{-5}	−0.492 790	3.3×10^{-5}
cyclopentadiene	−0.807 157	−0.807 080	7.7×10^{-5}	−0.807 080	7.7×10^{-5}
norbornadiene	−1.137 341	−1.137 264	7.7×10^{-5}	−1.137 264	7.7×10^{-5}
benzene	−0.939 401	−0.939 347	5.4×10^{-5}	−0.939 347	5.4×10^{-5}
naphthalene	−1.537 366	−1.537 288	7.8×10^{-5}	−1.537 288	7.8×10^{-5}
furan	−0.857 677	−0.857 469	2.1×10^{-4}	−0.857 469	2.1×10^{-4}
pyrrole	−0.836 547	−0.836 444	1.0×10^{-4}	−0.836 444	1.0×10^{-4}
imidazole	−0.865 179	−0.865 023	1.6×10^{-4}	−0.865 023	1.6×10^{-4}
pyridine	−0.968 200	−0.968 035	1.7×10^{-4}	−0.968 035	1.7×10^{-4}
pyrazine	−0.998 919	−0.998 795	1.2×10^{-4}	−0.998 795	1.2×10^{-4}
pyrimidine	−0.994 847	−0.994 602	2.5×10^{-4}	−0.994 602	2.5×10^{-4}
pyridazine	−1.003 959	−1.003 804	1.5×10^{-4}	−1.003 804	1.5×10^{-4}
triazine	−1.019 467	−1.019 106	3.6×10^{-4}	−1.019 106	3.6×10^{-4}
tetrazine	−1.068 661	−1.068 384	2.8×10^{-4}	−1.068 384	2.8×10^{-4}
formaldehyde	−0.400 267	−0.400 264	3.6×10^{-6}	−0.400 264	3.6×10^{-6}
acetone	−0.750 418	−0.750 305	1.1×10^{-4}	−0.750 305	1.1×10^{-4}
benzoquinone	−1.360 323	−1.359 716	6.1×10^{-4}	−1.359 716	6.1×10^{-4}
formamide	−0.601 044	−0.601 090	4.5×10^{-5}	−0.601 090	4.5×10^{-5}
acetamide	−0.775 695	−0.775 803	1.1×10^{-4}	−0.775 803	1.1×10^{-4}
propanamide	−0.953 435	−0.953 162	2.7×10^{-4}	−0.953 162	2.7×10^{-4}
adenine	−1.716 516	−1.715 858	6.6×10^{-4}	−1.715 858	6.6×10^{-4}
cytosine	−1.422 304	−1.421 549	7.5×10^{-4}	−1.421 549	7.5×10^{-4}
thymine	−1.623 172	−1.622 436	7.4×10^{-4}	−1.622 436	7.4×10^{-4}
uracil	−1.444 001	−1.443 282	7.2×10^{-4}	−1.443 282	7.2×10^{-4}

5.2.2 Excited State

Table S9: Detailed results for the excitation energies to the first three singlet excited states for the molecules in the benchmark set from Ref. S6. Errors of the MO- and CDD-based THC-SOS-ADC(2) methods are reported as deviations from the RI-based reference implementation. All calculations are performed with the cc-pVTZ/cc-pVTZ-RI basis set combination.

molecule	state	E_{RI} / eV	E_{THC} / eV	ΔE_{THC} / eV	$E_{\text{CDD-THC}}$ / eV	$\Delta E_{\text{CDD-THC}}$ / eV
ethene	S1	8.357	8.351	0.005	8.351	0.005
	S2	8.732	8.738	0.006	8.738	0.006
	S3	8.930	8.928	0.002	8.928	0.002
butadiene	S1	6.549	6.528	0.021	6.528	0.021
	S2	8.011	7.961	0.050	7.961	0.050
	S3	8.098	8.088	0.010	8.088	0.010
hexatriene	S1	5.504	5.512	0.008	5.512	0.008
	S2	7.075	7.081	0.007	7.081	0.007
	S3	7.421	7.427	0.006	7.427	0.006
octatetraene	S1	4.835	4.842	0.007	4.842	0.007
	S2	6.308	6.315	0.007	6.315	0.007
	S3	6.836	6.843	0.007	6.843	0.007
cyclopropene	S1	7.090	7.094	0.004	7.094	0.004
	S2	7.232	7.237	0.004	7.237	0.004
	S3	8.245	8.263	0.019	8.263	0.019
cyclopentadiene	S1	5.699	5.708	0.008	5.708	0.008
	S2	7.360	7.372	0.012	7.372	0.012
	S3	8.253	8.264	0.011	8.264	0.011
norbornadiene	S1	5.826	5.837	0.011	5.837	0.011
	S2	6.817	6.827	0.010	6.827	0.010
	S3	7.608	7.623	0.015	7.623	0.015
benzene	S1	5.030	5.037	0.008	5.037	0.008
	S2	6.393	6.406	0.013	6.406	0.013
	S3	7.499	7.509	0.009	7.509	0.009
naphthalene	S1	4.302	4.311	0.008	4.311	0.008
	S2	4.918	4.930	0.012	4.930	0.012
	S3	6.348	6.357	0.009	6.357	0.009
furan	S1	6.713	6.729	0.016	6.729	0.016
	S2	6.850	6.857	0.008	6.857	0.008
	S3	7.810	7.825	0.015	7.825	0.015
pyrrole	S1	6.496	6.499	0.004	6.499	0.004
	S2	6.632	6.640	0.007	6.640	0.007
	S3	6.795	6.801	0.006	6.801	0.006
imidazole	S1	6.672	6.679	0.007	6.679	0.007
	S2	6.987	6.991	0.004	6.991	0.004
	S3	7.040	7.044	0.004	7.044	0.004
pyridine	S1	5.078	5.086	0.008	5.086	0.008
	S2	5.345	5.353	0.008	5.353	0.008
	S3	5.694	5.702	0.008	5.702	0.008
pyrazine	S1	4.507	4.514	0.008	4.514	0.008
	S2	4.924	4.931	0.007	4.931	0.007
	S3	5.351	5.361	0.010	5.361	0.010
pyrimidine	S1	4.751	4.759	0.008	4.759	0.008
	S2	5.093	5.101	0.009	5.101	0.009

	S3	5.253	5.260	0.007	5.260	0.007
pyridazine	S1	4.183	4.186	0.003	4.186	0.003
	S2	4.801	4.808	0.007	4.808	0.007
	S3	5.119	5.122	0.003	5.122	0.003
triazine	S1	4.864	4.871	0.007	4.871	0.007
	S2	5.034	5.041	0.007	5.041	0.007
	S3	5.117	5.125	0.008	5.125	0.008
tetrazine	S1	2.795	2.797	0.002	2.797	0.002
	S2	4.179	4.186	0.007	4.186	0.007
	S3	4.931	4.933	0.002	4.933	0.002
formaldehyde	S1	4.064	4.077	0.013	4.077	0.013
	S2	8.157	8.169	0.012	8.169	0.012
	S3	9.256	9.272	0.016	9.272	0.016
acetone	S1	4.448	4.462	0.013	4.462	0.013
	S2	7.642	7.642	0.000	7.642	0.000
	S3	9.068	9.077	0.009	9.077	0.009
benzoquinone	S1	3.132	3.153	0.021	3.153	0.021
	S2	3.215	3.235	0.020	3.235	0.020
	S3	5.031	5.064	0.033	5.064	0.033
formamide	S1	5.623	5.631	0.008	5.631	0.008
	S2	7.831	7.837	0.006	7.837	0.006
	S3	7.530	7.542	0.013	7.542	0.013
acetamide	S1	5.657	5.667	0.010	5.667	0.010
	S2	7.542	7.553	0.011	7.553	0.011
	S3	7.616	7.619	0.003	7.619	0.003
propanamide	S1	5.680	5.692	0.011	5.692	0.011
	S2	7.525	7.541	0.015	7.541	0.015
	S3	7.615	7.619	0.003	7.619	0.003
adenine	S1	5.169	5.182	0.013	5.182	0.013
	S2	5.444	5.457	0.013	5.457	0.013
	S3	5.523	5.534	0.011	5.534	0.011
cytosine	S1	4.822	4.831	0.010	4.831	0.010
	S2	5.415	5.424	0.009	5.424	0.009
	S3	5.870	5.886	0.015	5.886	0.015
thymine	S1	5.023	5.030	0.007	5.030	0.007
	S2	5.468	5.482	0.014	5.482	0.014
	S3	6.419	6.435	0.016	6.435	0.016
uracil	S1	5.000	5.011	0.012	5.011	0.012
	S2	5.544	5.561	0.016	5.561	0.016
	S3	6.350	6.363	0.013	6.363	0.013

Table S10: Detailed results for the excitation energies to the first three triplet excited states for the molecules in the benchmark set from Ref. S6. Errors of the MO- and CDD-based THC-SOS-ADC(2) methods are reported as deviations from the RI-based reference implementation. All calculations are performed with the cc-pVTZ/cc-pVTZ-RI basis set combination.

molecule	state	E_{RI} / eV	E_{THC} / eV	ΔE_{THC} / eV	$E_{\text{CDD-THC}}$ / eV	$\Delta E_{\text{CDD-THC}}$ / eV
ethene	T1	4.570	4.568	0.002	4.568	0.002
	T2	8.584	8.590	0.006	8.590	0.006
	T3	8.620	8.618	0.002	8.618	0.002
butadiene	T1	3.511	3.515	0.004	3.515	0.004
	T2	5.216	5.219	0.003	5.219	0.003
	T3	7.987	7.991	0.004	7.991	0.004
hexatriene	T1	2.960	2.964	0.004	2.964	0.004
	T2	4.424	4.398	0.026	4.398	0.026
	T3	5.457	5.431	0.026	5.431	0.026
octatetraene	T1	2.598	2.602	0.004	2.602	0.004
	T2	3.785	3.789	0.003	3.789	0.003
	T3	4.861	4.864	0.003	4.864	0.003
cyclopropene	T1	4.466	4.468	0.002	4.468	0.002
	T2	6.830	6.833	0.003	6.833	0.003
	T3	7.909	7.912	0.003	7.912	0.003
cyclopentadiene	T1	3.414	3.418	0.004	3.418	0.004
	T2	5.085	5.088	0.004	5.088	0.004
	T3	7.308	7.319	0.011	7.319	0.011
norbornadiene	T1	3.873	3.879	0.006	3.879	0.006
	T2	4.207	4.213	0.005	4.213	0.005
	T3	6.870	6.879	0.009	6.879	0.009
benzene	T1	4.298	4.303	0.006	4.303	0.006
	T2	4.991	4.998	0.007	4.998	0.007
	T3	6.010	6.017	0.007	6.017	0.007
naphthalene	T1	3.300	3.306	0.006	3.306	0.006
	T2	4.271	4.278	0.007	4.278	0.007
	T3	4.760	4.766	0.007	4.766	0.007
furan	T1	4.334	4.343	0.010	4.343	0.010
	T2	5.445	5.451	0.006	5.451	0.006
	T3	7.236	7.241	0.005	7.241	0.005
pyrrole	T1	4.639	4.642	0.003	4.642	0.003
	T2	6.603	6.610	0.007	6.610	0.007
	T3	7.057	7.060	0.003	7.060	0.003
imidazole	T1	4.845	4.848	0.003	4.848	0.003
	T2	5.821	5.824	0.003	5.824	0.003
	T3	6.765	6.769	0.004	6.769	0.004
pyridine	T1	4.449	4.454	0.005	4.454	0.005
	T2	4.924	4.930	0.006	4.930	0.006
	T3	5.145	5.151	0.006	5.151	0.006
pyrazine	T1	3.969	3.974	0.005	3.974	0.005
	T2	4.529	4.533	0.004	4.533	0.004
	T3	5.355	5.360	0.006	5.360	0.006
pyrimidine	T1	4.447	4.453	0.006	4.453	0.006
	T2	4.705	4.710	0.004	4.710	0.004
	T3	5.066	5.071	0.005	5.071	0.005
pyridazine	T1	3.562	3.563	0.001	3.563	0.001

	T2	4.419	4.421	0.002	4.421	0.002
	T3	4.877	4.879	0.002	4.879	0.002
triazine	T1	4.743	4.749	0.006	4.749	0.006
	T2	4.820	4.826	0.006	4.826	0.006
	T3	5.093	5.097	0.004	5.097	0.004
tetrazine	T1	2.240	2.240	0.000	2.240	0.000
	T2	3.956	3.961	0.005	3.961	0.005
	T3	4.486	4.487	0.001	4.487	0.001
formaldehyde	T1	3.678	3.688	0.011	3.688	0.011
	T2	5.917	5.924	0.007	5.924	0.007
	T3	7.842	7.851	0.009	7.851	0.009
acetone	T1	4.145	4.157	0.012	4.157	0.012
	T2	6.133	6.139	0.007	6.139	0.007
	T3	7.563	7.563	0.000	7.563	0.000
benzoquinone	T1	2.868	2.888	0.020	2.888	0.020
	T2	2.964	2.984	0.019	2.984	0.019
	T3	3.236	3.255	0.019	3.255	0.019
formamide	T1	5.386	5.394	0.008	5.394	0.008
	T2	5.923	5.933	0.009	5.933	0.009
	T3	7.506	7.511	0.005	7.511	0.005
acetamide	T1	5.443	5.453	0.010	5.453	0.010
	T2	6.057	6.067	0.010	6.067	0.010
	T3	7.309	7.311	0.002	7.311	0.002
propanamide	T1	5.470	5.481	0.010	5.481	0.010
	T2	6.075	6.089	0.013	6.089	0.013
	T3	7.308	7.311	0.003	7.311	0.003
adenine	T1	4.171	4.179	0.008	4.179	0.008
	T2	5.034	5.043	0.010	5.043	0.010
	T3	5.339	5.344	0.005	5.344	0.005
cytosine	T1	3.969	3.975	0.006	3.975	0.006
	T2	4.843	4.850	0.007	4.850	0.007
	T3	5.280	5.287	0.007	5.287	0.007
thymine	T1	3.872	3.878	0.006	3.878	0.006
	T2	4.857	4.863	0.006	4.863	0.006
	T3	5.606	5.618	0.012	5.618	0.012
uracil	T1	3.968	3.977	0.009	3.977	0.009
	T2	4.835	4.846	0.011	4.846	0.011
	T3	5.619	5.632	0.014	5.632	0.014

Table S11: Detailed results for the excitation energies to the first three singlet excited states for the molecules in the benchmark set from Ref. S6. Errors of the MO- and CDD-based THC-SOS-LR-CC2 methods are reported as deviations from the RI-based reference implementation. All calculations are performed with the cc-pVTZ/cc-pVTZ-RI basis set combination.

molecule	state	E_{RI} / eV	E_{THC} / eV	ΔE_{THC} / eV	$E_{\text{CDD-THC}}$ / eV	$\Delta E_{\text{CDD-THC}}$ / eV
ethene	S1	8.409	8.368	0.041	8.368	0.041
	S2	8.702	8.658	0.044	8.658	0.044
	S3	9.484	9.442	0.042	9.442	0.042
butadiene	S1	6.613	6.585	0.028	6.585	0.028
	S2	7.991	7.955	0.036	7.955	0.036
	S3	8.067	8.042	0.025	8.042	0.025
hexatriene	S1	5.567	5.556	0.012	5.556	0.012
	S2	7.059	7.034	0.026	7.034	0.026
	S3	7.384	7.376	0.007	7.376	0.007
octatetraene	S1	4.896	4.884	0.012	4.884	0.012
	S2	6.301	6.279	0.023	6.279	0.023
	S3	6.890	6.879	0.011	6.879	0.011
cyclopropene	S1	7.075	7.071	0.004	7.071	0.004
	S2	7.279	7.256	0.023	7.256	0.023
	S3	8.224	8.217	0.008	8.217	0.008
cyclopentadiene	S1	5.745	5.728	0.017	5.728	0.017
	S2	7.269	7.240	0.029	7.240	0.029
	S3	7.326	7.301	0.025	7.301	0.025
norbornadiene	S1	5.851	5.856	0.005	5.856	0.005
	S2	6.813	6.812	0.000	6.812	0.000
	S3	7.574	7.587	0.012	7.587	0.012
benzene	S1	5.018	5.000	0.018	5.000	0.018
	S2	6.429	6.420	0.009	6.420	0.009
	S3	7.511	7.499	0.013	7.499	0.013
naphthalene	S1	4.299	4.287	0.012	4.287	0.012
	S2	4.953	4.951	0.002	4.951	0.002
	S3	6.368	6.360	0.008	6.360	0.008
furan	S1	6.712	6.702	0.010	6.702	0.010
	S2	6.850	6.822	0.028	6.822	0.028
	S3	7.765	7.746	0.019	7.746	0.019
pyrrole	S1	6.493	6.459	0.034	6.459	0.034
	S2	6.558	6.558	0.000	6.558	0.000
	S3	6.797	6.790	0.006	6.790	0.006
imidazole	S1	6.676	6.648	0.029	6.648	0.029
	S2	6.979	6.956	0.023	6.956	0.023
	S3	7.018	7.005	0.013	7.005	0.013
pyridine	S1	5.070	5.016	0.054	5.016	0.054
	S2	5.345	5.335	0.010	5.335	0.010
	S3	5.703	5.687	0.016	5.687	0.016
pyrazine	S1	4.475	4.471	0.004	4.471	0.004
	S2	4.905	4.875	0.030	4.875	0.030
	S3	5.316	5.314	0.002	5.314	0.002
pyrimidine	S1	4.770	4.755	0.015	4.755	0.015
	S2	5.129	5.109	0.020	5.109	0.020
	S3	5.264	5.212	0.052	5.212	0.052
pyridazine	S1	4.163	4.161	0.001	4.161	0.001

	S2	4.772	4.777	0.005	4.777	0.005
	S3	5.111	5.068	0.043	5.068	0.043
triazine	S1	4.931	4.906	0.026	4.906	0.026
	S2	5.085	5.065	0.020	5.065	0.020
	S3	5.141	5.125	0.016	5.125	0.016
tetrazine	S1	2.748	2.739	0.009	2.739	0.009
	S2	4.123	4.117	0.006	4.117	0.006
	S3	4.931	4.876	0.055	4.876	0.055
formaldehyde	S1	4.218	4.197	0.021	4.197	0.021
	S2	8.243	8.213	0.030	8.213	0.030
	S3	9.409	9.411	0.002	9.411	0.002
acetone	S1	4.633	4.644	0.011	4.644	0.011
	S2	7.712	7.709	0.002	7.709	0.002
	S3	9.250	9.234	0.017	9.234	0.017
benzoquinone	S1	3.269	3.291	0.021	3.291	0.021
	S2	3.382	3.409	0.027	3.409	0.027
	S3	4.970	4.994	0.024	4.994	0.024
formamide	S1	5.853	5.844	0.010	5.844	0.010
	S2	7.673	7.650	0.022	7.650	0.022
	S3	7.808	7.777	0.031	7.777	0.031
acetamide	S1	5.888	5.879	0.009	5.879	0.009
	S2	7.594	7.570	0.024	7.570	0.024
	S3	7.653	7.642	0.011	7.642	0.011
propanamide	S1	5.912	5.937	0.025	5.937	0.025
	S2	7.594	7.574	0.021	7.574	0.021
	S3	7.653	7.663	0.010	7.663	0.010
adenine	S1	5.212	5.190	0.022	5.190	0.022
	S2	5.521	5.504	0.017	5.504	0.017
	S3	5.584	5.579	0.005	5.579	0.005
cytosine	S1	4.977	4.932	0.045	4.932	0.045
	S2	5.556	5.539	0.017	5.539	0.017
	S3	6.015	5.979	0.037	5.979	0.037
thymine	S1	5.264	5.253	0.011	5.253	0.011
	S2	5.552	5.546	0.006	5.546	0.006
	S3	6.660	6.639	0.021	6.639	0.021
uracil	S1	5.247	5.226	0.021	5.226	0.021
	S2	5.650	5.632	0.018	5.632	0.018
	S3	6.591	6.589	0.002	6.589	0.002

Table S12: Detailed results for the excitation energies to the first three triplet excited states for the molecules in the benchmark set from Ref. S6. Errors of the MO- and CDD-based THC-SOS-LR-CC2 methods are reported as deviations from the RI-based reference implementation. All calculations are performed with the cc-pVTZ/cc-pVTZ-RI basis set combination.

molecule	state	E_{RI} / eV	E_{THC} / eV	ΔE_{THC} / eV	$E_{\mathrm{CDD\text{-}THC}}$ / eV	$\Delta E_{\mathrm{CDD\text{-}THC}}$ / eV
ethene	T1	4.570	4.535	0.035	4.535	0.035
	T2	8.552	8.510	0.043	8.510	0.043
	T3	8.622	8.612	0.009	8.612	0.009
butadiene	T1	3.553	3.525	0.027	3.525	0.027
	T2	5.258	5.236	0.022	5.236	0.022
	T3	7.956	7.945	0.011	7.945	0.011
hexatriene	T1	2.967	2.955	0.012	2.955	0.012
	T2	4.432	4.418	0.013	4.418	0.013
	T3	5.464	5.455	0.009	5.455	0.009
octatetraene	T1	2.606	2.594	0.012	2.594	0.012
	T2	3.795	3.780	0.014	3.780	0.014
	T3	4.870	4.858	0.012	4.858	0.012
cyclopropene	T1	7.075	7.071	0.004	7.070	0.005
	T2	7.279	7.256	0.023	7.260	0.019
	T3	8.224	8.217	0.008	8.220	0.004
cyclopentadiene	T1	3.424	3.404	0.020	3.404	0.020
	T2	5.090	5.070	0.020	5.070	0.020
	T3	7.275	7.250	0.025	7.250	0.025
norbornadiene	T1	3.884	3.881	0.003	3.881	0.003
	T2	4.211	4.204	0.006	4.204	0.006
	T3	6.861	6.859	0.002	6.859	0.002
benzene	T1	4.296	4.280	0.016	4.280	0.016
	T2	4.999	4.983	0.016	4.983	0.016
	T3	6.042	6.034	0.008	6.034	0.008
naphthalene	T1	3.308	3.301	0.008	3.301	0.008
	T2	4.284	4.276	0.008	4.276	0.008
	T3	4.768	4.759	0.010	4.759	0.010
furan	T1	4.354	4.338	0.016	4.338	0.016
	T2	5.478	5.452	0.026	5.452	0.026
	T3	7.248	7.229	0.019	7.229	0.019
pyrrole	T1	4.650	4.637	0.013	4.637	0.013
	T2	6.531	6.530	0.001	6.530	0.001
	T3	7.046	7.027	0.019	7.027	0.019
imidazole	T1	4.863	4.842	0.021	4.842	0.021
	T2	5.835	5.810	0.025	5.810	0.025
	T3	6.621	6.607	0.014	6.607	0.014
pyridine	T1	4.447	4.419	0.028	4.419	0.028
	T2	4.935	4.895	0.040	4.895	0.040
	T3	5.157	5.119	0.038	5.119	0.038
pyrazine	T1	3.948	3.941	0.007	3.941	0.007
	T2	4.485	4.465	0.020	4.465	0.020
	T3	4.537	4.519	0.018	4.519	0.018
pyrimidine	T1	4.452	4.439	0.013	4.439	0.013
	T2	4.694	4.663	0.031	4.663	0.031
	T3	5.090	5.051	0.039	5.051	0.039
pyridazine	T1	3.553	3.546	0.007	3.546	0.007

	T2	4.423	4.398	0.024	4.398	0.024
	T3	4.891	4.864	0.027	4.864	0.027
triazine	T1	4.743	4.731	0.012	4.731	0.012
	T2	4.850	4.833	0.018	4.833	0.018
	T3	5.068	5.036	0.032	5.036	0.032
tetrazine	T1	2.204	2.192	0.011	2.192	0.011
	T2	3.909	3.901	0.008	3.901	0.008
	T3	4.492	4.458	0.034	4.458	0.034
formaldehyde	T1	3.806	3.785	0.021	3.785	0.021
	T2	5.982	5.930	0.052	5.930	0.052
	T3	7.911	7.882	0.029	7.882	0.029
acetone	T1	4.301	4.310	0.010	4.310	0.010
	T2	6.216	6.178	0.038	6.178	0.038
	T3	7.629	7.627	0.002	7.627	0.002
benzoquinone	T1	2.987	3.010	0.022	3.010	0.022
	T2	3.107	3.133	0.025	3.133	0.025
	T3	3.264	3.262	0.002	3.262	0.002
formamide	T1	5.586	5.578	0.008	5.578	0.008
	T2	5.992	5.958	0.034	5.958	0.034
	T3	7.473	7.444	0.029	7.444	0.029
acetamide	T1	5.644	5.635	0.009	5.635	0.009
	T2	6.136	6.103	0.033	6.103	0.033
	T3	7.279	7.257	0.023	7.257	0.023
propanamide	T1	5.672	5.693	0.021	5.693	0.021
	T2	6.155	6.134	0.021	6.134	0.021
	T3	7.280	7.261	0.019	7.261	0.019
adenine	T1	4.190	4.174	0.016	4.174	0.016
	T2	5.057	5.051	0.006	5.051	0.006
	T3	5.373	5.354	0.019	5.354	0.019
cytosine	T1	4.032	4.010	0.022	4.010	0.022
	T2	4.909	4.886	0.022	4.886	0.022
	T3	5.396	5.379	0.017	5.379	0.017
thymine	T1	3.915	3.909	0.006	3.909	0.006
	T2	5.071	5.062	0.010	5.062	0.010
	T3	5.669	5.658	0.012	5.658	0.012
uracil	T1	4.013	3.998	0.015	3.998	0.015
	T2	5.055	5.036	0.019	5.036	0.019
	T3	5.686	5.667	0.019	5.667	0.019

5.3 Martínez Benchmark Set

5.3.1 Ground State

Table S13: Detailed results for the ground state energies of the molecules in the benchmark set from Ref. S7. Errors of the MO- and CDD-based THC-SOS-MP2 methods are reported as deviations from the RI-based reference implementation. All calculations are performed with the cc-pVTZ/cc-pVTZ-RI basis set combination.

molecule	E_{RI} / H	E_{THC} / H	ΔE_{THC} / H	$E_{\mathrm{CDD\text{-}THC}}$ / H	$\Delta E_{\mathrm{CDD\text{-}THC}}$ / H
butadiene	−0.658 266	−0.658 268	1.3×10^{-6}	−0.658 268	1.5×10^{-6}
hexatriene	−0.971 382	−0.971 378	4.0×10^{-6}	−0.971 378	4.3×10^{-6}
malonaldehyde	−0.928 473	−0.928 469	4.3×10^{-6}	−0.928 469	4.4×10^{-6}
methylsalicylate	−1.910 410	−1.910 398	1.2×10^{-5}	−1.910 398	1.2×10^{-5}
HBT	−2.543 654	−2.543 520	1.3×10^{-4}	−2.543 520	1.3×10^{-4}
stilbene	−2.151 922	−2.151 908	1.4×10^{-5}	−2.151 908	1.4×10^{-5}
GFP	−2.335 196	−2.335 177	1.9×10^{-5}	−2.335 177	1.9×10^{-5}
PYP	−2.046 621	−2.046 610	1.1×10^{-5}	−2.046 610	1.1×10^{-5}
acridine red	−2.913 065	−2.912 992	7.4×10^{-5}	−2.912 992	7.3×10^{-5}
anthanthrene	−3.281 459	−3.281 362	9.7×10^{-5}	−3.281 362	9.7×10^{-5}
N-methyl-benzcarbaxole	−2.783 274	−2.783 203	7.0×10^{-5}	−2.783 203	7.1×10^{-5}
coumarin 153	−3.849 149	−3.849 068	8.1×10^{-5}	−3.849 068	8.1×10^{-5}
difluoro-indigo	−3.626 637	−3.626 523	1.1×10^{-4}	−3.626 523	1.1×10^{-4}
nile red	−3.927 931	−3.927 846	8.5×10^{-5}	−3.927 846	8.5×10^{-5}
oxazine 9	−3.187 605	−3.187 530	7.5×10^{-5}	−3.187 530	7.5×10^{-5}
rubicene	−3.878 599	−3.878 435	1.6×10^{-4}	−3.878 435	1.6×10^{-4}
ester	−3.384 816	−3.384 736	7.9×10^{-5}	−3.384 736	8.0×10^{-5}

Table S14: Detailed results for the ground state energies of the molecules in the benchmark set from Ref. S7. Errors of the MO- and CDD-based THC-SOS-CC2 methods are reported as deviations from the RI-based reference implementation. All calculations are performed with the cc-pVTZ/cc-pVTZ-RI basis set combination.

molecule	E_{RI} / H	E_{THC} / H	ΔE_{THC} / H	$E_{\text{CDD-THC}}$ / H	$\Delta E_{\text{CDD-THC}}$ / H
butadiene	0.663 446	−0.663 380	6.6×10^{-5}	−0.663 380	6.6×10^{-5}
hexatriene	0.979 143	−0.979 049	9.4×10^{-5}	−0.979 049	9.4×10^{-5}
malonaldehyde	0.941 556	−0.940 910	6.5×10^{-4}	−0.940 910	6.5×10^{-4}
methylsalicylate	1.931 444	−1.930 642	8.0×10^{-4}	−1.930 642	8.0×10^{-4}
HBT	2.567 426	−2.566 600	8.3×10^{-4}	−2.566 600	8.3×10^{-4}
stilbene	2.167 465	−2.167 414	5.1×10^{-5}	−2.167 414	5.1×10^{-5}
GFP	2.360 494	−2.359 745	7.5×10^{-4}	−2.359 745	7.5×10^{-4}
PYP	2.068 245	−2.067 535	7.1×10^{-4}	−2.067 535	7.1×10^{-4}
acridine red	2.943 776	−2.941 744	2.0×10^{-3}	−2.941 744	2.0×10^{-3}
anthanthrene	3.307 075	−3.306 840	2.4×10^{-4}	−3.306 840	2.4×10^{-4}
N-methyl-benzcarbaxole	2.805 027	−2.804 581	4.5×10^{-4}	−2.804 581	4.5×10^{-4}
coumarin 153	3.887 952	−3.886 496	1.5×10^{-3}	−3.886 496	1.5×10^{-3}
difluoro-indigo	3.668 254	−3.667 169	1.1×10^{-3}	−3.667 169	1.1×10^{-3}
nile red	3.966 831	−3.965 170	1.7×10^{-3}	−3.965 170	1.7×10^{-3}
oxazine 9	3.221 792	−3.219 770	2.0×10^{-3}	−3.219 770	2.0×10^{-3}
rubicene	3.908 834	−3.908 510	3.2×10^{-4}	−3.908 510	3.2×10^{-4}
ester	3.426 647	−3.425 046	1.6×10^{-3}	−3.425 046	1.6×10^{-3}

5.3.2 Excited State

Table S15: Detailed results for the excitation energies to the first three singlet excited states for the molecules in the benchmark set from Ref. S7. Errors of the MO- and CDD-based THC-SOS-ADC(2) methods are reported as deviations from the RI-based reference implementation. All calculations are performed with the cc-pVTZ/cc-pVTZ-RI basis set combination.

molecule	state	E_{RI} / eV	E_{THC} / eV	ΔE_{THC} / eV	$E_{\text{CDD-THC}}$ / eV	$\Delta E_{\text{CDD-THC}}$ / eV
butadiene	S1	6.549	6.557	0.008	6.557	0.008
	S2	8.011	8.016	0.005	8.016	0.005
	S3	8.098	8.103	0.005	8.103	0.005
hexatriene	S1	5.504	5.512	0.008	5.512	0.008
	S2	7.075	7.081	0.007	7.081	0.007
	S3	7.421	7.427	0.006	7.427	0.006
malonaldehyde	S1	4.179	4.189	0.009	4.189	0.009
	S2	4.935	4.949	0.014	4.949	0.014
	S3	7.816	7.826	0.010	7.826	0.010
methylsalicylate	S1	4.289	4.301	0.012	4.301	0.012
	S2	5.517	5.530	0.013	5.530	0.013
	S3	5.565	5.581	0.016	5.581	0.016
HBT	S1	3.961	3.973	0.012	3.973	0.012
	S2	4.426	4.439	0.013	4.439	0.013
	S3	4.784	4.800	0.016	4.800	0.016
stilbene	S1	4.484	4.492	0.008	4.492	0.008
	S2	4.647	4.654	0.007	4.654	0.007
	S3	4.689	4.697	0.007	4.697	0.007
GFP	S1	3.919	3.934	0.015	3.934	0.015
	S2	4.195	4.208	0.013	4.208	0.013
	S3	4.494	4.503	0.009	4.503	0.009
PYP	S1	4.486	4.495	0.009	4.495	0.009
	S2	4.685	4.695	0.009	4.695	0.009
	S3	5.076	5.089	0.012	5.089	0.012
acridine red	S1	2.655	2.670	0.015	2.670	0.015
	S2	3.538	3.552	0.014	3.552	0.014
	S3	4.475	4.489	0.014	4.489	0.014
anthanthrene	S1	3.211	3.222	0.010	3.222	0.010
	S2	3.297	3.318	0.021	3.318	0.021
	S3	3.944	3.973	0.029	3.973	0.029
N-methyl-benzcarbaxole	S1	3.537	3.550	0.013	3.550	0.013
	S2	4.137	4.152	0.015	4.152	0.015
	S3	4.582	4.594	0.011	4.594	0.011
coumarin 153	S1	3.474	3.497	0.023	3.497	0.023
	S2	4.095	4.114	0.019	4.114	0.019
	S3	4.793	4.815	0.022	4.815	0.022
difluoro-indigo	S1	2.553	2.566	0.013	2.566	0.013
	S2	2.950	2.966	0.016	2.966	0.016
	S3	3.361	3.377	0.017	3.377	0.017
nile red	S1	2.905	2.921	0.017	2.921	0.017
	S2	3.402	3.410	0.007	3.410	0.007
	S3	3.820	3.861	0.041	3.861	0.041
oxazine 9	S1	2.349	2.360	0.010	2.360	0.011
	S2	3.239	3.249	0.011	3.249	0.011

	S3	3.971	3.975	0.004	3.975	0.004
rubicene	S1	2.838	2.857	0.019	2.860	0.022
	S2	3.209	3.229	0.019	3.229	0.019
	S3	3.412	3.431	0.019	3.431	0.019
ester	S1	4.025	4.047	0.022	4.047	0.022
	S2	4.702	4.726	0.024	4.726	0.024
	S3	5.098	5.118	0.020	5.118	0.020

Table S16: Detailed results for the excitation energies to the first three triplet excited states for the molecules in the benchmark set from Ref. S7. Errors of the MO- and CDD-based THC-SOS-ADC(2) methods are reported as deviations from the RI-based reference implementation. All calculations are performed with the cc-pVTZ/cc-pVTZ-RI basis set combination.

molecule	state	E_{RI} / eV	E_{THC} / eV	ΔE_{THC} / eV	$E_{\mathrm{CDD\text{-}THC}}$ / eV	$\Delta E_{\mathrm{CDD\text{-}THC}}$ / eV
butadiene	T1	3.548	3.552	0.004	3.552	0.004
	T2	5.253	5.256	0.003	5.256	0.003
	T3	7.979	7.983	0.004	7.983	0.004
hexatriene	T1	2.960	2.964	0.004	2.964	0.004
	T2	4.424	4.428	0.003	4.428	0.003
	T3	5.457	5.461	0.003	5.461	0.003
malonaldehyde	T1	3.673	3.682	0.009	3.682	0.009
	T2	3.987	3.995	0.009	3.995	0.009
	T3	6.325	6.332	0.007	6.332	0.007
methylsalicylate	T1	3.922	3.982	0.059	3.982	0.059
	T2	4.163	4.171	0.008	4.171	0.008
	T3	4.689	4.697	0.009	4.697	0.009
HBT	T1	3.359	3.370	0.011	3.370	0.011
	T2	4.149	4.157	0.007	4.157	0.007
	T3	4.389	4.401	0.012	4.401	0.012
stilbene	T1	2.966	2.970	0.004	2.970	0.004
	T2	4.100	4.105	0.005	4.105	0.005
	T3	4.578	4.588	0.010	4.588	0.010
GFP	T1	2.544	2.554	0.010	2.554	0.010
	T2	4.054	4.060	0.007	4.060	0.007
	T3	4.481	4.490	0.009	4.490	0.009
PYP	T1	3.181	3.187	0.006	3.187	0.006
	T2	4.572	4.576	0.004	4.576	0.004
	T3	4.823	4.830	0.006	4.830	0.006
acridine red	T1	2.303	2.316	0.013	2.316	0.013
	T2	3.302	3.315	0.013	3.315	0.013
	T3	3.613	3.624	0.011	3.624	0.011
anthanthrene	T1	1.969	1.983	0.014	1.983	0.014
	T2	3.158	3.170	0.012	3.170	0.012
	T3	3.364	3.375	0.011	3.375	0.011
N-methyl-	T1	2.863	2.873	0.011	2.873	0.011
benzcarbaxole	T2	3.624	3.634	0.010	3.634	0.010
	T3	3.899	3.909	0.010	3.909	0.010
coumarin 153	T1	2.779	2.796	0.018	2.796	0.018
	T2	3.942	3.960	0.018	3.960	0.018
	T3	4.281	4.295	0.014	4.295	0.014
difluoro-indigo	T1	2.304	2.364	0.060	2.364	0.060

	T2	3.039	3.052	0.013	3.052	0.013
	T3	3.550	3.563	0.013	3.563	0.013
nile red	T1	2.079	2.092	0.014	2.092	0.014
	T2	3.249	3.257	0.008	3.257	0.008
	T3	3.742	3.754	0.012	3.754	0.012
oxazine 9	T1	1.821	1.830	0.009	1.830	0.009
	T2	2.999	3.008	0.009	3.008	0.009
	T3	3.558	3.569	0.011	3.569	0.011
rubicene	T1	1.885	1.900	0.015	1.900	0.015
	T2	2.796	2.809	0.014	2.809	0.014
	T3	2.838	2.852	0.014	2.852	0.014
ester	T1	2.797	2.812	0.015	2.812	0.015
	T2	3.848	3.862	0.014	3.862	0.014
	T3	4.149	4.165	0.016	4.165	0.016

Table S17: Detailed results for the excitation energies to the first three singlet excited states for the molecules in the benchmark set from Ref. S7. Errors of the MO- and CDD-based THC-SOS-LR-CC2 methods are reported as deviations from the RI-based reference implementation. All calculations are performed with the cc-pVTZ/cc-pVTZ-RI basis set combination.

molecule	state	E_{RI} / eV	E_{THC} / eV	ΔE_{THC} / eV	$E_{\text{CDD-THC}}$ / eV	$\Delta E_{\text{CDD-THC}}$ / eV
butadiene	S1	6.613	6.585	0.028	6.585	0.028
	S2	7.991	7.955	0.036	7.955	0.036
	S3	8.067	8.042	0.025	8.042	0.025
hexatriene	S1	5.567	5.556	0.012	5.556	0.012
	S2	7.059	7.034	0.026	7.034	0.026
	S3	7.384	7.376	0.007	7.376	0.007
malonaldehyde	S1	4.397	4.390	0.007	4.390	0.007
	S2	5.103	5.064	0.039	5.064	0.039
	S3	7.865	7.846	0.020	7.846	0.020
methylsalicylate	S1	4.338	4.319	0.020	4.319	0.020
	S2	5.643	5.634	0.009	5.634	0.009
	S3	5.732	5.726	0.006	5.726	0.006
HBT	S1	4.010	3.997	0.012	3.997	0.012
	S2	4.454	4.440	0.014	4.440	0.014
	S3	4.850	4.837	0.013	4.837	0.013
stilbene	S1	4.502	4.491	0.010	4.491	0.010
	S2	4.640	4.623	0.017	4.623	0.017
	S3	4.697	4.683	0.014	4.683	0.014
GFP	S1	4.030	4.014	0.016	4.014	0.016
	S2	4.342	4.338	0.004	4.338	0.004
	S3	4.506	4.488	0.018	4.488	0.018
PYP	S1	4.517	4.498	0.019	4.498	0.019
	S2	4.769	4.749	0.019	4.749	0.019
	S3	5.314	5.295	0.019	5.295	0.019
acridine red	S1	2.861	2.794	0.067	2.790	0.071
	S2	3.683	3.652	0.031	3.650	0.033
	S3	4.580	4.556	0.024	4.560	0.020
anthanthrene	S1	3.208	3.201	0.007	3.201	0.007
	S2	3.330	3.331	0.000	3.331	0.000
	S3	3.949	3.964	0.015	3.964	0.015

N-methyl-	S1	3.556	3.553	0.004	3.550	0.006
benzcarbaxole	S2	4.147	4.149	0.001	4.150	0.003
	S3	4.605	4.597	0.008	4.600	0.005
coumarin 153	S1	3.577	3.580	0.003	3.580	0.003
	S2	4.147	4.157	0.011	4.160	0.013
	S3	4.850	4.864	0.014	4.864	0.014
difluoro-indigo	S1	2.629	2.639	0.010	2.639	0.010
	S2	3.051	3.067	0.015	3.067	0.015
	S3	3.397	3.396	0.001	3.396	0.001
nile red	S1	2.997	2.981	0.016	2.981	0.016
	S2	3.629	3.616	0.014	3.616	0.014
	S3	3.865	3.869	0.004	3.869	0.004
oxazine 9	S1	2.563	2.562	0.001	2.562	0.001
	S2	3.355	3.356	0.007	3.356	0.007
	S3	4.046	4.045	0.017	4.045	0.017
rubicene	S1	2.852	2.860	0.008	2.860	0.008
	S2	3.215	3.225	0.010	3.225	0.010
	S3	3.413	3.425	0.012	3.425	0.012
ester	S1	4.080	4.078	0.003	4.078	0.003
	S2	4.770	4.766	0.004	4.766	0.004
	S3	5.144	5.137	0.007	5.137	0.007

Table S18: Detailed results for the excitation energies to the first three triplet excited states for the molecules in the benchmark set from Ref. S7. Errors of the MO- and CDD-based THC-SOS-LR-CC2 methods are reported as deviations from the RI-based reference implementation. All calculations are performed with the cc-pVTZ/cc-pVTZ-RI basis set combination.

molecule	state	E_{RI} / eV	E_{THC} / eV	ΔE_{THC} / eV	$E_{\mathrm{CDD\text{-}THC}}$ / eV	$\Delta E_{\mathrm{CDD\text{-}THC}}$ / eV
butadiene	T1	3.553	3.525	0.027	3.525	0.027
	T2	5.258	5.236	0.022	5.236	0.022
	T3	7.956	7.945	0.011	7.945	0.011
hexatriene	T1	2.967	2.955	0.012	2.955	0.012
	T2	4.432	4.418	0.013	4.418	0.013
	T3	5.464	5.455	0.009	5.455	0.009
malonaldehyde	T1	3.738	3.711	0.027	3.711	0.027
	T2	4.173	4.169	0.004	4.169	0.004
	T3	6.436	6.401	0.035	6.401	0.035
methylsalicylate	T1	3.971	3.958	0.013	3.958	0.013
	T2	4.175	4.167	0.008	4.167	0.008
	T3	4.724	4.711	0.013	4.711	0.013
HBT	T1	3.392	3.387	0.005	3.387	0.005
	T2	4.154	4.147	0.007	4.147	0.007
	T3	4.419	4.413	0.006	4.413	0.006
stilbene	T1	2.973	2.962	0.010	2.962	0.010
	T2	4.102	4.092	0.010	4.092	0.010
	T3	4.593	4.583	0.010	4.583	0.010
GFP	T1	2.579	2.569	0.010	2.569	0.010
	T2	4.082	4.071	0.011	4.071	0.011
	T3	4.495	4.485	0.010	4.485	0.010
PYP	T1	3.214	3.198	0.016	3.198	0.016
	T2	4.589	4.576	0.013	4.576	0.013

	T3	4.846	4.833	0.013	4.833	0.013
acridine red	T1	2.381	2.361	0.020	2.361	0.020
	T2	3.411	3.389	0.022	3.389	0.022
	T3	3.712	3.678	0.033	3.678	0.033
anthanthrene	T1	1.968	1.969	0.001	1.969	0.001
	T2	3.168	3.165	0.003	3.165	0.003
	T3	3.706	3.711	0.005	3.711	0.005
N-methyl-	T1	2.867	2.866	0.001	2.866	0.001
benzcarbaxole	T2	3.638	3.633	0.004	3.633	0.004
	T3	3.919	3.916	0.003	3.916	0.003
coumarin 153	T1	2.831	2.837	0.006	2.837	0.006
	T2	4.003	4.008	0.004	4.008	0.004
	T3	4.362	4.342	0.019	4.342	0.019
difluoro-indigo	T1	2.369	2.364	0.005	2.364	0.005
	T2	3.061	3.058	0.003	3.058	0.003
	T3	3.590	3.586	0.003	3.586	0.003
nile red	T1	2.120	2.118	0.002	2.118	0.002
	T2	3.451	3.439	0.012	3.311	0.140
	T3	3.786	3.775	0.011	3.775	0.011
oxazine 9	T1	1.905	1.872	0.032	1.872	0.032
	T2	3.090	3.057	0.033	3.057	0.033
	T3	3.620	3.601	0.019	3.601	0.019
rubicene	T1	1.888	1.892	0.004	1.892	0.004
	T2	2.796	2.802	0.007	2.802	0.007
	T3	2.844	2.850	0.006	2.850	0.006
ester	T1	2.824	2.823	0.001	2.823	0.001
	T2	3.874	3.871	0.004	3.871	0.004
	T3	4.214	4.210	0.004	4.210	0.004

6 Detailed Results – Computational Efficiency

6.1 Time Complexity

In the following, supplementary results for the computational complexity of the proposed THC-based reformulations of the methods presented in the main part of this work are given.

The computational complexity of the MO/CDD-THC-SOS-MP2 methods is assessed by taking into account the number of FLOP required to evaluate the SOS-MP2 singles amplitude for the LCA_n (Figure S3) and the AT_n series (Figure S4).

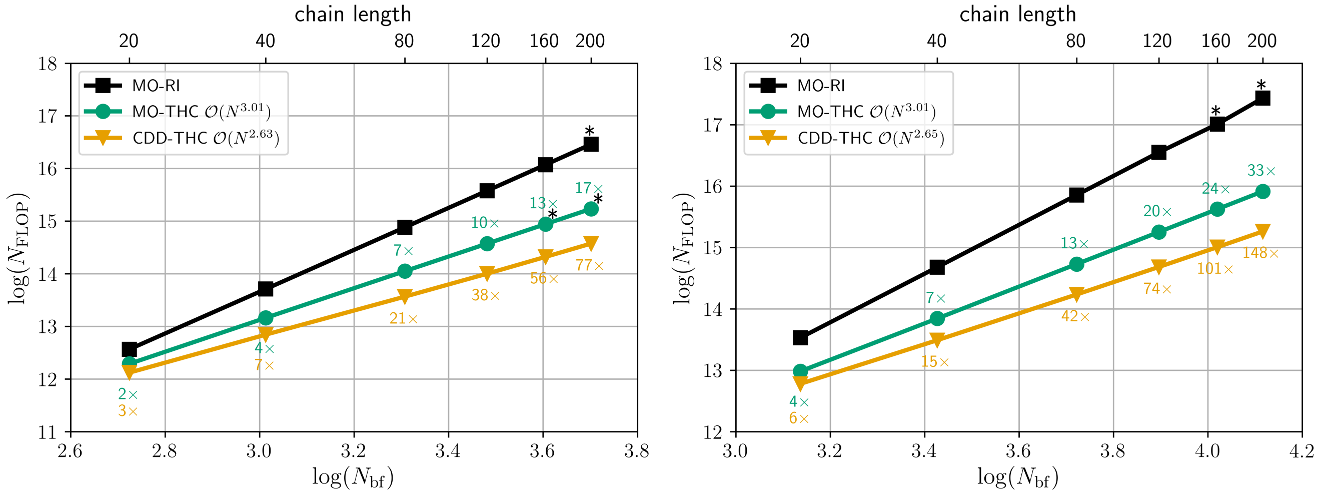

Figure S3: Number of FLOP required to evaluate the SOS-MP2 singles amplitudes for a series of LCA_n ($n \in \{20, 40, 80, 120, 160, 200\}$) molecules. Labels represent the FLOP reduction compared to the MO-RI-SOS-MP2 implementation and all calculations are performed with the cc-pVDZ/cc-pVDZ-RI (left) or cc-pVTZ/cc-pVTZ-RI basis set combination (right). Extrapolated values are marked with an asterisk (*).

For the LCA_n series, MO-THC-SOS-MP2 exhibits exactly cubic scaling with the system size for both basis set sizes considered. Overall, in comparison to the RI-based reference implementation, the application of MO-THC yields a 17-fold diminution of the effort for the double-ζ basis set and a 33-fold diminution of the effort for the larger triple-ζ basis set. Furthermore, a reformulation in the local Cholesky MO basis results in improvements of up to 148-fold for the largest LCA molecule considered, while reducing the apparent scaling exponent to $\sim$2.5. In the limit of the largest fragment sizes considered, the observed scaling behavior is similar for the less-sparse AT_n

series, as shown in Figure S4.

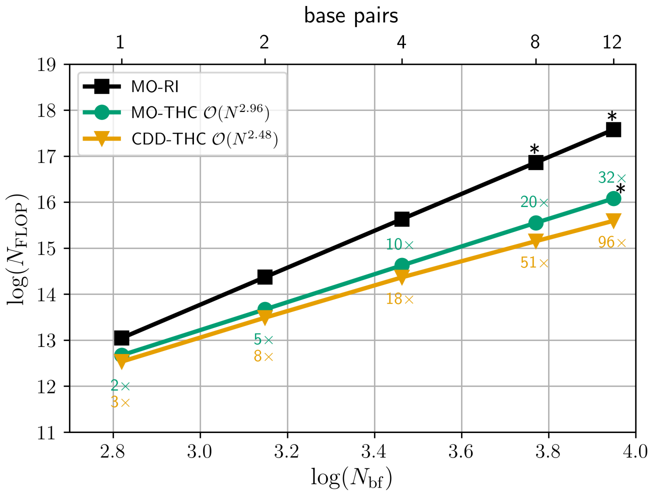

Figure S4: Number of FLOP required to evaluate the SOS-MP2 singles amplitudes for a series of AT_n ($n \in \{1, 2, 4, 8, 12\}$) molecules. Labels represent the FLOP reduction compared to the MO-RI-SOS-MP2 implementation and all calculations are performed with the cc-pVDZ/cc-pVDZ-RI basis set combination. Extrapolated values are marked with an asterisk (*).

In addition to the results presented in the main part of this work for the larger triple-ζ basis set, Figure S5 shows the scaling behavior of the THC-SOS-LR-CC2/SOS-ADC(2) methods for the LCA_n series using the cc-pVDZ/cc-pVDZ-RI basis set combination.

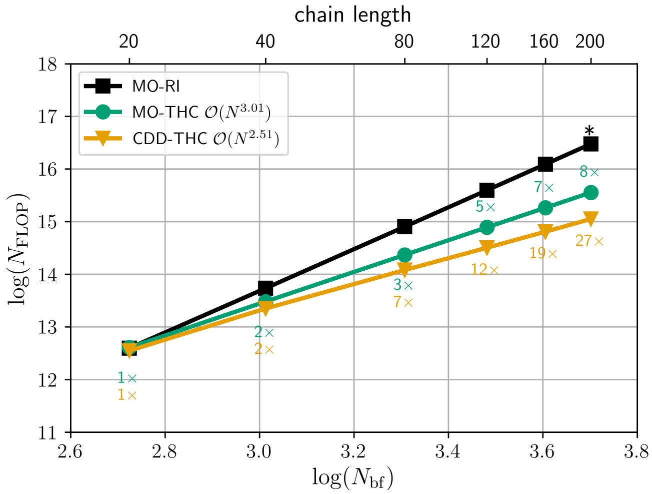

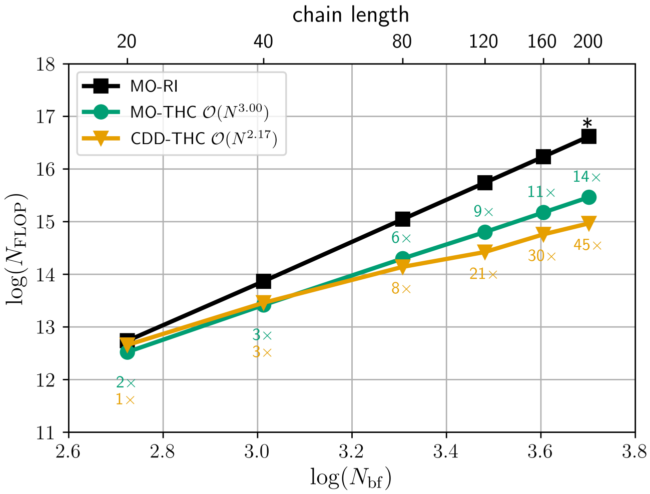

Figure S5: Number of FLOP required to evaluate the SOS-CC2 singles amplitudes (left) and the SOS-LR-CC2/SOS-ADC(2) matrix-vector product (right) for a series of LCA_n ($n \in \{20, 40, 80, 120, 160, 200\}$) molecules. Labels represent the FLOP reduction compared to the MO-RI-SOS-LR-CC2/ADC(2) implementation and all calculations are performed with the cc-pVDZ/cc-pVDZ-RI basis set combination. Extrapolated values are marked with an asterisk (*).

6.1.1 THC Grid Comparison

Table S19: Average number of PFLOP (10^{15} FLOP) required per DIIS iteration to form the matrix-vector product in MO-THC-SOS-ADC(2) and CDD-THC-SOS-ADC(2). Additionally, the ×-fold reduction in the number of FLOP is shown with respect to MO-RI-SOS-ADC(2). All calculations are performed using the cc-pVDZ/cc-pVDZ-RI basis set combination and the grids generated from both the hand-optimized cc-pVDZ- (*grid 1*) and cc-pVTZ-based parent grids (*grid 2*) from Martínez and coworkers.[S2] Extrapolated values are marked with an asterisk (*).

			MO-RI	MO-THC			CDD-THC		
	System	N_{bf}	PFLOP	PFLOP	Scaling	Reduction	PFLOP	Scaling	Reduction
grid 1									
	AT_{04}	2904	6.2	0.3	—	20.3×	0.5	—	12.4×
	AT_{08}	5896	110.0*	2.6	3.0	41.0×	2.6	2.3	40.0×
	AT_{12}	5896	537.0*	8.8	3.0	61.0×	2.6	2.3	76.4×
	AT_{16}	11880	1800.0*	21.0	3.0	83.5×	15.0	2.5	119.3×
grid 2									
	AT_{04}	2904	6.2	0.7	—	8.9×	0.9	—	6.9×
	AT_{08}	5896	110.0*	5.9	3.0	17.8×	6.8	2.8	15.4×
	AT_{12}	5896	537.0*	20.0	3.0	41.0×	2.6	2.3	40.0×
	AT_{16}	11880	1800.0*	49.1*	3.0	35.7×	38.0*	2.5	46.1×

Table S20: Time required to perform one iteration of the excited state calculation. Scaling and speedups are reported for the THC-based methods relative to MO-RI-SOS-ADC(2) using the cc-pVDZ/cc-pVDZ-RI basis set combination together with the grids generated from both the hand-optimized cc-pVDZ- (*grid 1*) and cc-pVTZ-based parent grids (*grid 2*). Extrapolated values are marked with an asterisk (*).

			MO-RI	MO-THC		CDD-THC	
	System	N_{bf}	Time / h	Time / h	Speedup	Time / h	Speedup
grid 1							
	AT_{4}	2904	1.1	0.1	8.9×	0.3	4.1×
	AT_{8}	5896	19.0*	0.8	23.4×	1.0	18.8×
	AT_{12}	8888	98.7*				
	AT_{16}	11 880	312.2*	6.6*	47.0×	4.6	68.1×
grid 2							
	AT_{4}	2904	1.1	0.4	2.6×	0.5	2.2×
	AT_{8}	5896	19.0*	2.3	8.3×	2.5	7.7×
	AT_{12}	8888	98.7*	8.0*	8.3×	2.1	8.9×
	AT_{16}	11 880	312.2*	19.0*	16.4×	11.1*	28.1×

6.1.2 THC Z vs. Γ Tensor

In the following, the scaling of the computational effort required to evaluate the THC-SOS-LR-CC2/ADC(2) matrix-vector product using either a formulation of the intermediates in terms of the THC **Z** or the **Γ** tensor is exemplarily demonstrated for a series of LCA$_n$ molecules in Figure S6. Here, the label CDD-THC (**Γ**) indicates the results obtained by using eqs. (d)–(f) in Table 2 in the main part of this work, whereas the CDD-THC (**Z**) label indicates the results obtained using eqs. (e')–(f') in the same table.

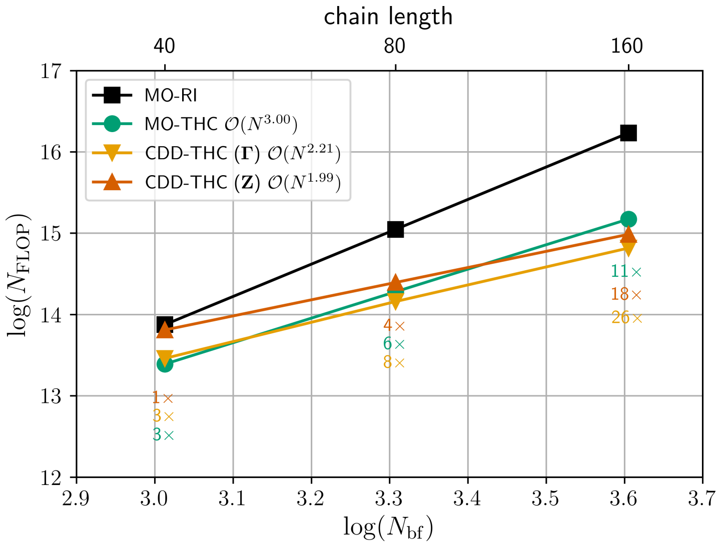

Figure S6: Number of FLOP required to evaluate the SOS-LR-CC2/SOS-ADC(2) matrix-vector product for a series of LCA$_n$ ($n \in \{40, 80, 160\}$) molecules. Labels represent the FLOP reduction compared to the MO-RI-SOS-LR-CC2/ADC(2) implementation. All calculations are performed with the cc-pVDZ/cc-pVDZ-RI basis set combination and the hand-optimized cc-pVDZ-based THC parent grid.

As expected, the MO-THC-based implementation exhibits cubic time complexity, whereas the use of the THC **Γ** or the THC **Z** tensor in conjunction with the CDD approach reduces the scaling exponent to ∼2.2 or ∼2.0, respectively. Despite the scaling advantage, the use of the THC **Γ** tensor is favorable in terms of the number of FLOP required to evaluate the CDD-THC-SOS-LR-CC2/ADC(2) matrix-vector due to the reduced dimension of the **Γ** vs. the **Z** tensor, i.e., $(N_{\text{grid}} \times N_{\text{aux}})$ vs. $(N_{\text{grid}} \times N_{\text{grid}})$.

6.2 Space Complexity

The memory demand of relevant intermediates in both ground and excited state calculations in the block sparse format is directly proportional to the number of significant and therefore allocated blocks within a given intermediate. The observed space complexity of the intermediates computed within the CDD-THC-SOS-CC2 and CDD-THC-SOS-LR-CC2/ADC(2) algorithms scale at most as $\mathcal{O}(N^{1.5})$ with the system size for both LCAs – Figures S7–S10 in the cc-pVTZ basis – and the AT pairs – Figures S11–S12 in the cc-pVDZ basis. Notice that the memory demands of the THC fitting matrices scale as $\mathcal{O}(N^2)$ since they are always dense. Thus, in the case of CDD-based reformulations and electronically sparse systems, the memory demands are dominated by either the $\mathbf{\Gamma}$ or $\mathbf{Z}$ THC fitting matrices and formally scale as $\mathcal{O}(N^2)$. Nonetheless, the reduction of the space complexity and the significant memory savings for the intermediates are crucial to extend the application of the proposed methods to systems with hundreds of atoms without the need to batch the work load.

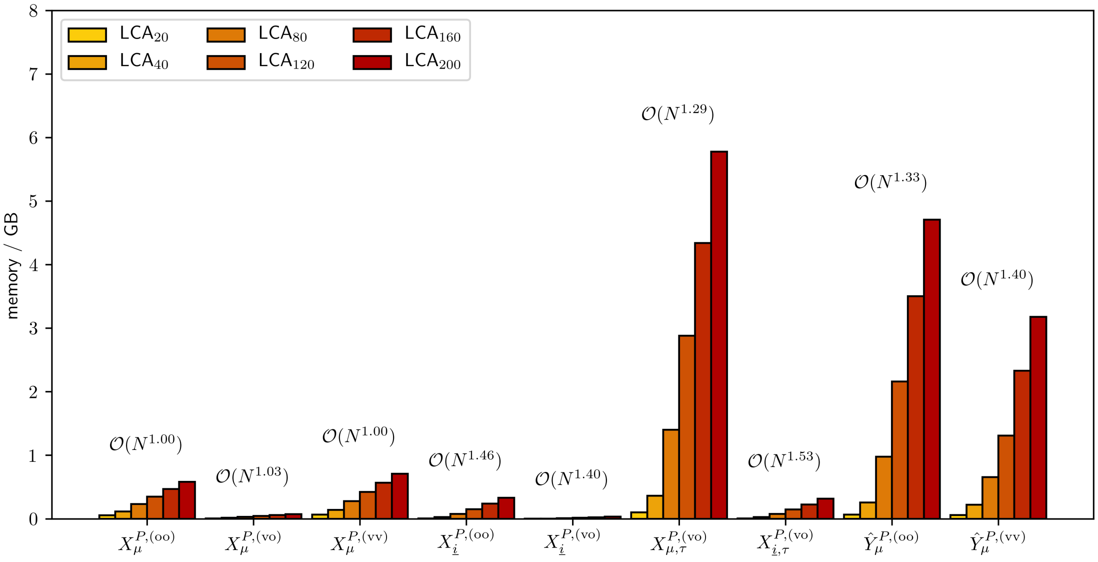

Figure S7: Scaling of the memory requirements of various intermediates of the CDD-THC-SOS-CC2 method. Here relevant intermediates of dimension $N_{\text{grid}} \times N_{\text{bf}}$ or $N_{\text{grid}} \times N_{\text{occ}}$ are included. For Laplace point-dependent intermediates the values are averaged over the number of quadrature points. All calculations are performed for a series of LCA$_n$ molecules with the cc-pVTZ/cc-pVTZ-RI basis set combination.

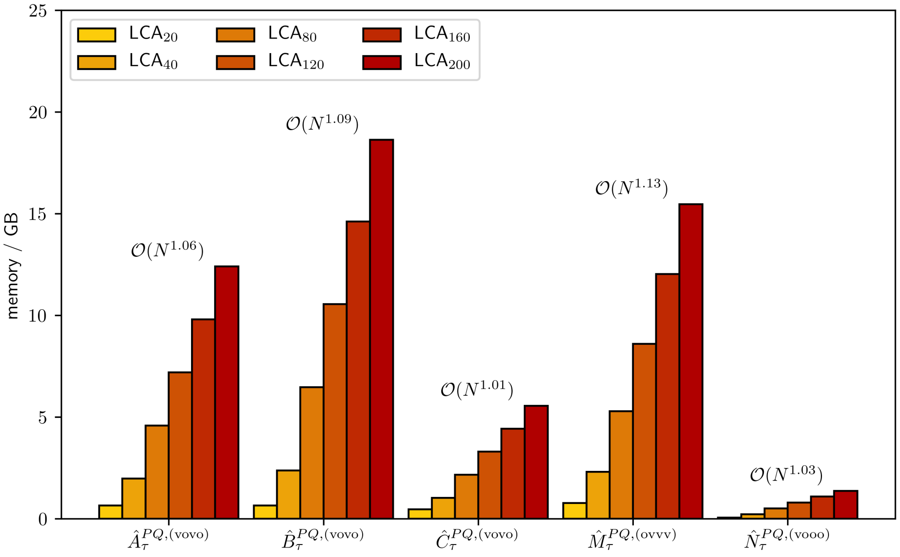

Figure S8: Scaling of the memory requirements of various intermediates of the CDD-THC-SOS-CC2 method. Here relevant intermediates of dimension $N_{\text{grid}} \times N_{\text{grid}}$ are included. For Laplace point-dependent intermediates the values are averaged over the number of quadrature points. All calculations are performed for a series of LCA$_n$ molecules with the cc-pVTZ/cc-pVTZ-RI basis set combination.

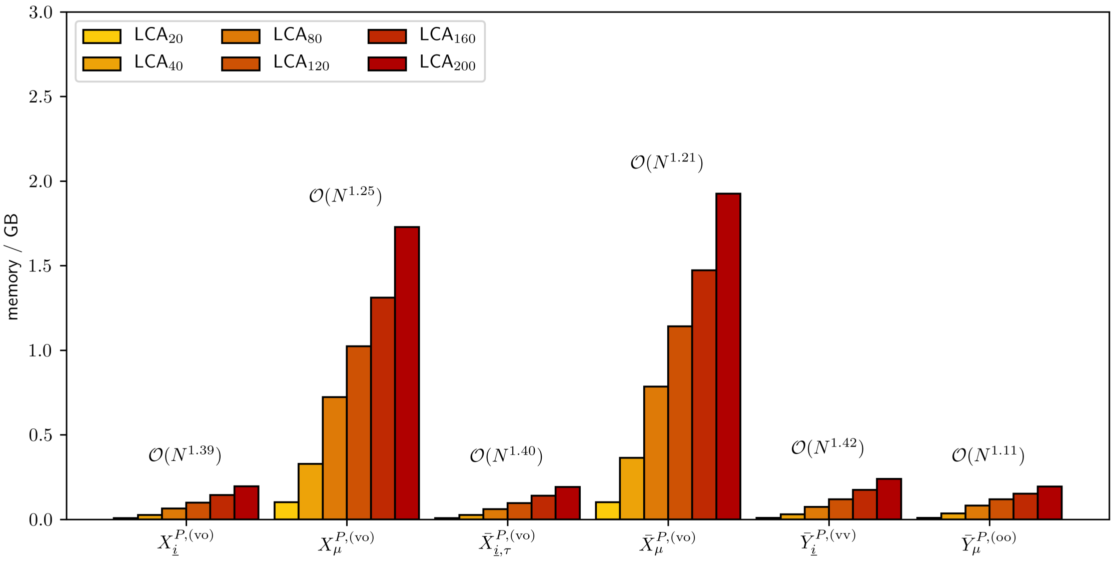

Figure S9: Scaling of the memory requirements of various intermediates of the CDD-THC-SOS-ADC(2) method. Here relevant intermediates of dimension $N_{\text{grid}} \times N_{\text{bf}}$ or $N_{\text{grid}} \times N_{\text{occ}}$ are included. For Laplace point-dependent intermediates the values are averaged over the number of quadrature points. All calculations are performed for a series of LCA$_n$ molecules with the cc-pVTZ/cc-pVTZ-RI basis set combination.

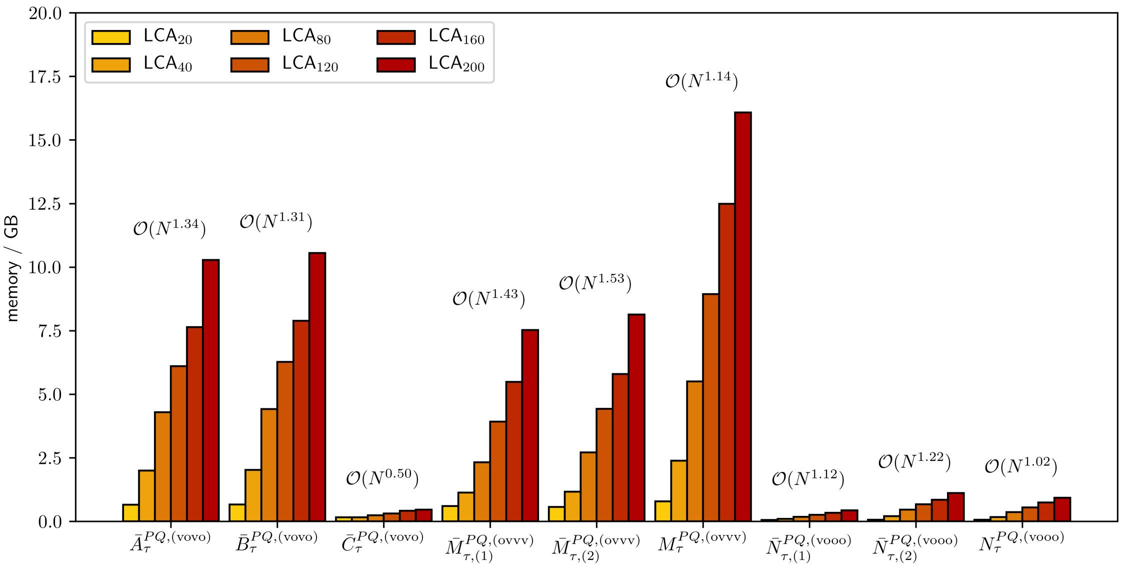

Figure S10: Scaling of the memory requirements of various intermediates of the CDD-THC-SOS-ADC(2) method. Here relevant intermediates of dimension $N_{\text{grid}} \times N_{\text{grid}}$ are included. For Laplace point-dependent intermediates the values are averaged over the number of quadrature points. All calculations are performed for a series of LCA$_n$ molecules with the cc-pVTZ/cc-pVTZ-RI basis set combination.

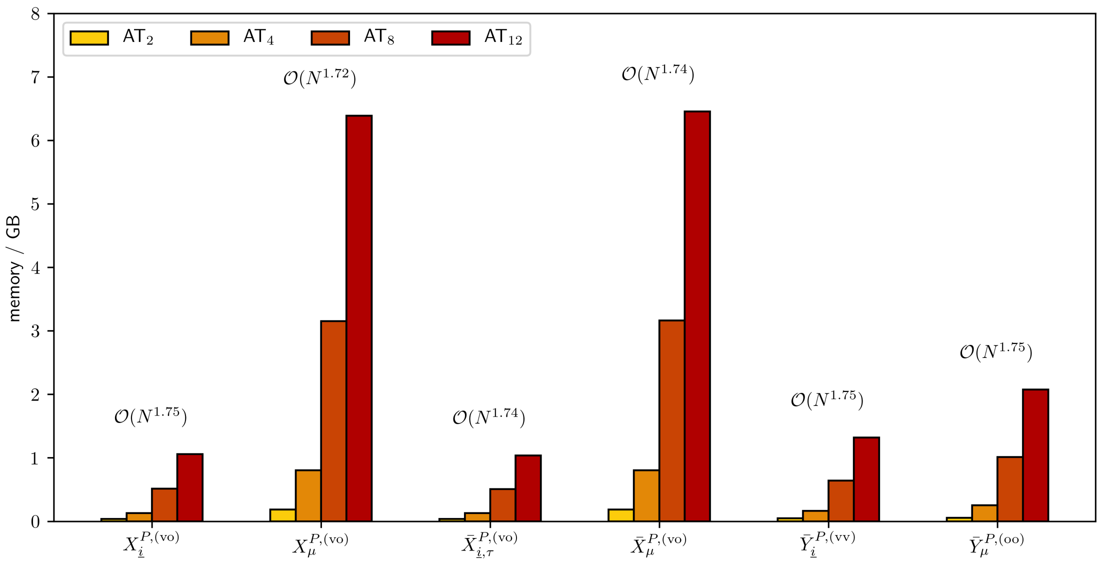

Figure S11: Scaling of the memory requirements of various intermediates of the CDD-THC-SOS-ADC(2) method. Here relevant intermediates of dimension $N_{\text{grid}} \times N_{\text{bf}}$ or $N_{\text{grid}} \times N_{\text{occ}}$ are included. For Laplace point-dependent intermediates the values are averaged over the number of quadrature points. All calculations are performed for a series of AT$_n$ molecules with the cc-pVDZ/cc-pVDZ-RI basis set combination.

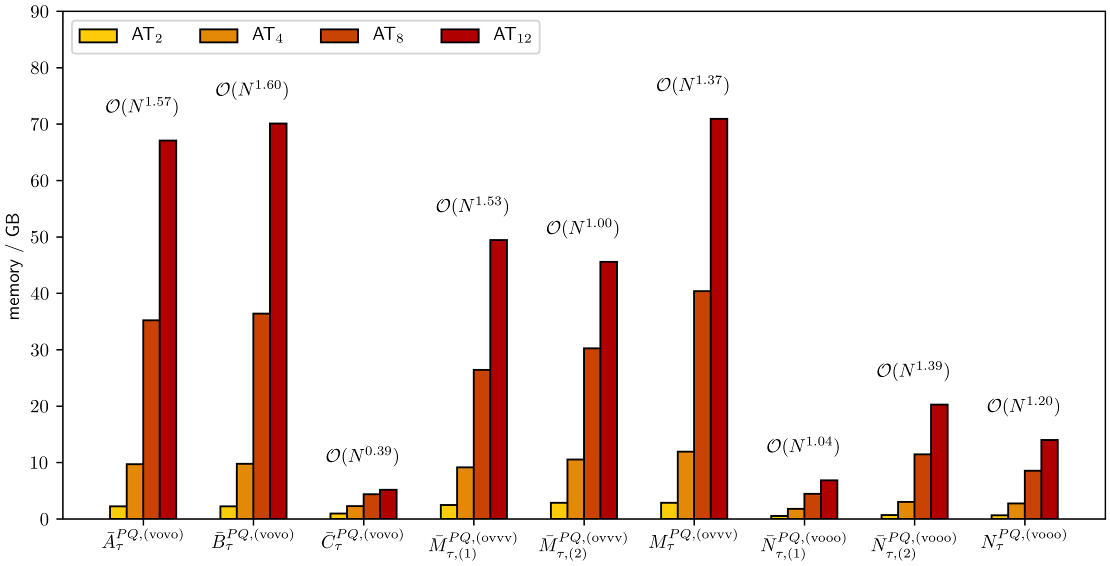

Figure S12: Scaling of the memory requirements of various intermediates of the CDD-THC-SOS-ADC(2) method. Here relevant intermediates of dimension $N_{\text{grid}} \times N_{\text{grid}}$ are included. For Laplace point-dependent intermediates the values are averaged over the number of quadrature points. All calculations are performed for a series of AT$_n$ molecules with the cc-pVDZ/cc-pVDZ-RI basis set combination.

Taking AT$_{12}$ as an illustrative example, one set of MO-based RI integrals using the cc-pVDZ/cc-pVDZ-RI basis set combination would require ~6 TB of memory/disk space, with a $<$ 20% saving if Cholesky pseudo-MOs and sparse algebra are put to use. Hence, considering that at least one other set of integrals must be stored in the CDD-RI-SOS-ADC(2) implementation, both the number of input/output (I/O) operations (IOP) as well as storage demands easily become a bottleneck for common high-performance compute nodes and would render batching – with its intrinsic overhead – mandatory. In contrast, for the same basis set the evaluation of the SOS-ADC(2) and SOS-LR-CC2 excitation energy of AT$_{12}$ using THC only requires ~500 GB of memory space. In addition, the presented implementations of MO- and CDD-THC-SOS-ADC(2)/CC2 only require disk I/O for the $\hat{\mathbf{D}}$ intermediates (~72 GB for AT$_{12}$/cc-pVDZ/cc-pVDZ-RI) in the Laplace integration, while all other intermediates can be kept in memory.

References

(S1) Matthews, D. A. Improved Grid Optimization and Fitting in Least Squares Tensor Hypercontraction. *J. Chem. Theory Comput.* **2020**, *16*, 1382–1385.

(S2) Kokkila Schumacher, S. I. L.; Hohenstein, E. G.; Parrish, R. M.; Wang, L. P.; Martínez, T. J. Tensor Hypercontraction Second-Order Møller-Plesset Perturbation Theory: Grid Optimization and Reaction Energies. *J. Chem. Theory Comput.* **2015**, *11*, 3042–3052.

(S3) Parrish, R. M.; Hohenstein, E. G.; Martínez, T. J.; Sherrill, C. D. Tensor Hypercontraction. II. Least-squares Renormalization. *J. Chem. Phys.* **2012**, *137*, 224106.

(S4) Cuthill, E.; McKee, J. Reducing the Bandwidth of Sparse Symmetric Matrices. *Proceedings of the 1969 24th National Conference, ACM 1969* **1969**, 157–172.

(S5) DiStasio, R. A.; Jung, Y.; Head-Gordon, M. A resolution-of-the-identity implementation of the local triatomics-in-molecules model for second-order Møller- Plesset perturbation theory with application to alanine tetrapeptide conformational energies. *J. Chem. Theory Comput.* **2005**, *1*, 862–876.

(S6) Schreiber, M.; Silva-Junior, M. R.; Sauer, S.; Thiel, W. Benchmarks for electronically excited states: CASPT2, CC2, CCSD, and CC3. *J. Chem. Phys.* **2008**, *128*.

(S7) Hohenstein, E. G.; Kokkila, S. I.; Parrish, R. M.; Martínez, T. J. Tensor Hypercontraction Equation-of-Motion Second-Order Approximate Coupled Cluster: Electronic Excitation Energies in O(N4) Time. *J. Phys. Chem. B* **2013**, *117*, 12972–12978.
